# Alcohol-Decorated Lignins for Nanoparticle Formation through Reactive Fractionation in Ternary Deep Eutectic Solvent Systems

**DOI:** 10.1021/acssuschemeng.5c07102

**Published:** 2026-02-19

**Authors:** Zhiwen Wang, Umberto Danelon, Roberto Sole, Claudia Crestini, Katalin Barta

**Affiliations:** † College of Forestry, Northwest Agriculture & Forestry University, Yangling 712100, China; ‡ Department of Chemistry, Organic and Bioorganic Chemistry, 27267 University of Graz, Heinrichstrasse 28/II, 8010 Graz, Austria; § Department of Molecular Sciences and Nanosystems, 19047 Ca’ Foscari University of Venice, Via Torino, 155, 30172 Mestre, VE, Italy

**Keywords:** deep eutectic solvent, lignocellulosic biomass, lignin isolation, high β-O-4, structure, nanoparticles

## Abstract

This study explores the use of ternary deep eutectic solvent (DES) systems composed of choline chloride (ChCl), oxalic acid (OA), and ethylene glycol (EG) for the efficient fractionation of lignocellulosic biomass and isolation of lignins with diverse structural features and EG incorporation levels. Reactive fractionation of birchwood under optimized conditions resulted in a high lignin yield (66%), with up to 75% retention of β-O-4 aryl ether linkages. Systematic variation of temperature (80–200 °C), reaction time, and DES composition showed that EG incorporation is both temperature- and time-dependent, with optimal structural preservation observed at 140–160 °C and short reaction times. Furthermore, 2D HSQC NMR and GPC analyses revealed that increasing temperatures promote the cleavage of aryl ether linkages, while a higher EG content in the DES mitigates structural degradation. The resulting EG-decorated lignins were successfully applied to the controlled synthesis of lignin nanoparticles (LNPs) via hydrotropic and pH-induced flash precipitation methods. The latter allowed one to obtain LNPs with size tunability, colloidal stability, and favorable surface charge, highlighting their potential for material applications. Overall, this study provides critical insights into the structure-processing relationships of DES-isolated lignins and establishes a promising approach to their valorization into functional nanomaterials.

## Graphical abstract

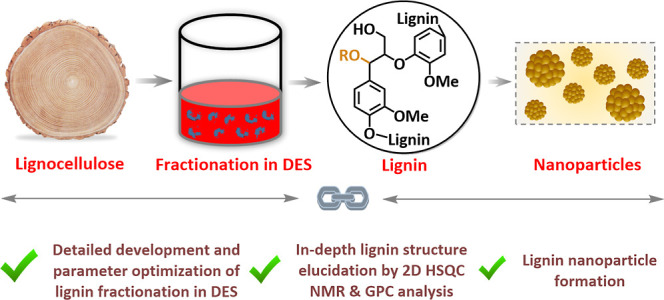

## 1 Introduction

Lignin is an intriguing, naturally abundant biopolymer, with true potential to serve as sustainable, renewable resource for the manufacturing of diverse chemicals and materials (Figure 1a).^1–4^ Specifically, taking advantage of its inherently rigid polymeric nature and modular structure, lignin shows great promise for the preparation of valuable materials with tailored properties and function.^5–7^ Prominent examples are lignin nanoparticles (LNPs) that serve as a versatile tool for harnessing lignin’s potential across diverse fields, including environmental and biomedical applications.^8,9^ Examples span from drug delivery systems, biocompatible adhesives, hydrogels, and wound-healing materials to UV protection in cosmetics and antimicrobial agents,^10–12^ whereby performance in these applications is closely related and strongly dependent on accessibility and tunability of different lignin functionalities. To this end, the development of novel fractionation strategies to reach high lignin recovery, purity, and suitable structural characteristics is crucially important.^13^

**Figure 1:**
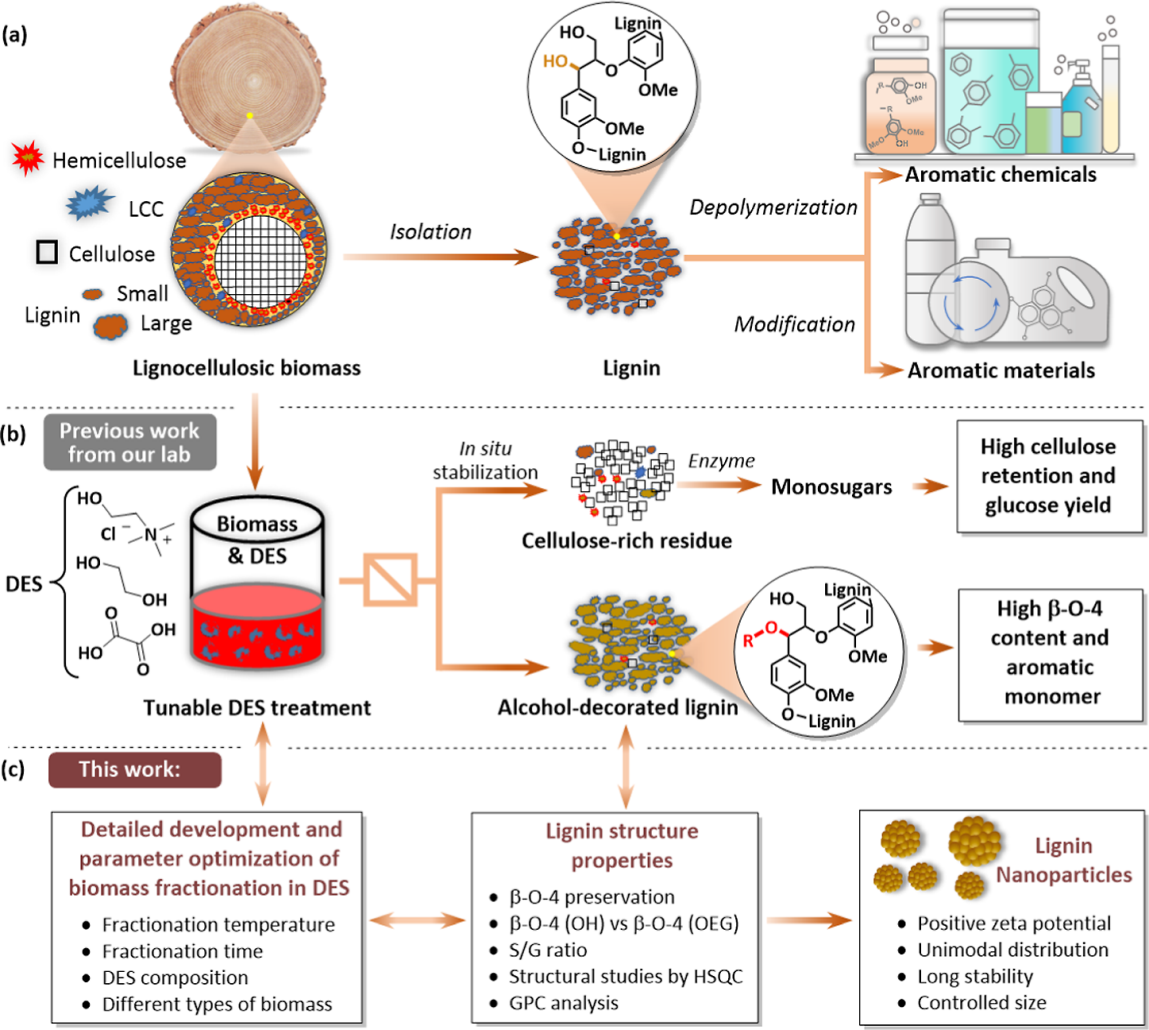
Lignocellulose fractionation in the ternary DES system. (a) Biomass fractionation and possible lignin valorization routes. (b) Previous work on using the choline chloride (ChCl)/ethylene glycol (EG)/oxalic acid (OA) ternary DES system for isolation of alcohol-decorated lignin via EG incorporation into the benzylic position. (c) This work provides an evaluation of the parameters influencing lignocellulose fractionation using the optimized ChCl/EG/OA DES approach and its impact on lignin structure. Additionally, it explores the preparation of LNPs from selected DES-derived lignin fractions.

It is also important to note that the lignin composition is highly dependent on the biomass source. The diverse monolignol units and their combinatorial couplings give rise to lignins with variable molecular weights, substitution patterns, and linkage distributions. These differences strongly influence how lignin is released from the plant cell wall as well as its dissolution and separation efficiency during fractionation. In particular, aromatic units with different degrees of methoxy substitution show varying recalcitrance, which directly impacts lignin recovery and downstream applications.^14,15^

Deep eutectic solvents (DESs) have emerged as powerful alternative for biomass fractionation strategies.^16^ Due to their favorable physical chemical properties, easy preparation, and tunable composition, DESs present a promising alternative to classical organic solvents typically used in biorefining.^17–22^ Common DES-based strategies include selective lignin solubilization, alkaline DES pretreatment to disrupt lignin–carbohydrate complexes and increase biomass porosity, and careful tuning of DES composition and processing conditions (e.g., temperature and water content) to optimize fractionation efficiency.^23,24^ For instance, recent studies showed that functional DES incorporating γ-valerolactone have enabled efficient lignin extraction and molecular structure modulation, facilitating the formation of homogeneous lignin nanoparticles.^25,26^ Integrated DES-based biorefinery strategies have also been reported for the coproduction of lignin together with glucose and furfural.^27^ In addition, phenolic aldehyde-based DES systems have demonstrated that DES acidity and hydrogen-bonding characteristics influence delignification efficiency and lignin structural preservation.^28^

Previously, we have pioneered the use of a compositionally tunable ternary DES system comprised of choline chloride (ChCl), oxalic acid (OA), and ethylene glycol (EG) for the mild fractionation of lignocellulose, simultaneously resulting in high-quality lignin and cellulose (Figure 1b).^20^ We have found that the addition of EG as a DES component suppresses undesired recondensation phenomena. Specifically, inclusion of EG as a hydrogen bond donor (HBD) component to the ternary DES suppressed the deposition of recondensed lignin on the cellulose residues, leading to excellent glucose yields upon biocatalytic processing.^20,22,29^ Moreover, the lignins so obtained exhibited ethylene glycol incorporation into the benzylic position of the β-O-4 moieties, leading to a high retention of these linkages, and markedly lower recondensation.^19,20,22,30^ Notably, the incorporation of ethylene glycol preserves hydroxyl functionalities, providing accessible reactive sites for further chemical modification, cross-linking, or grafting reactions. Finally, it was demonstrated that the DES system could be recycled multiple times with only minimal impact on lignin yield and quality,^19,20,31,32^ highlighting its potential for cost-effective and scalable applications.

Given the lack of in-depth studies with our previously studied ternary DES system (comprising ChCl/OA/EG), in this work, we set out to understand how the systematic variation of fractionation conditions affects the structure of the isolated lignins, with particular attention to the retention of β-O-4 linkages and EG incorporation. Upon variation of key parameters (DES composition, temperature and time, lignocellulose particle size), we have assessed the β-O-4 content and degree of EG incorporation, the extent of condensation, molecular weight, and polydispersity of the lignins isolated, as determined by multitechnique analysis including 2D HSQC NMR and gel permeation chromatography (GPC) (Figure 1c). Gratifyingly, under optimized conditions, a high yield (66%) of alcohol-decorated lignin was achieved, with up to 75% retention of β-O-4 aryl ether linkages. The optimized fractionation–isolation conditions were successfully extended to a wider range of biomass sources.

Overall, our results provide critical insight into process parameters and the resulting lignin structural variations, underscoring the great potential of this tunable ternary DES for isolating EG decorated lignin with tailored properties. Notably, we have shown that such lignins are particularly suitable for the controlled synthesis of LNPs via hydrotropic and pH-induced flash precipitation methods. The latter made it possible to obtain LNPs with size tunability, colloidal stability, and favorable surface charge, highlighting their potential for valorization into functional nanomaterials.

## 2 Methods

### 2.1 Biomass Preparation

Typically, birch, pine, walnut shell, and wheat straw were ground and then passed through sieves to collect biomass particles at a size range of 355–500 μm. Bagasse was solely smashed without passing through sieves. All of the obtained biomass was dewaxed (toluene/ethanol, 2/1, v/v) by a Soxhlet extractor for 8 h. After solvent removal, the processed biomass was further dried at 70 °C for 16 h and kept in sealed bags before further usage. Chemical composition of all the starting biomasses was determined according the method from NREL^33^ and is presented in Table S1.

### 2.2 DES Preparation

The DES was prepared following our previous publications.^20^ Normally, predesignated amounts of ChCl and EG were mixed together and vigorously stirred for 30 min in a 250 mL round-bottom flask. A predesignated amount of OA was added next, and the mixture was heated at the temperature used for lignin fractionation until a transparent liquid appeared.

### 2.3 Exemplified Procedure of Lignocellulose Fractionation via DES

The process for lignocellulose fractionation via DES is represented in Scheme 1. Lignocellulose fractionation was carried out in a 250 mL round-bottom flask. Typically, 3.12 g of OA was added to a premixed liquid mixture consisting of 16.8 g ChCl and 14.4 g EG at the specific temperature and heated under stirring until a transparent liquid formed. Afterward, 4 g of biomass was added to the mixture through a glass funnel. The mixture was heated and stirred at the predesignated temperature (step 1). After the predesignated retention time was reached, the flask was cooled using an ice bath. Upon cooling, 200 mL of 90% (v/v) ethanol was added to the mixture and stirred for 2 h (step 2). The resulting mixture was filtered by a glass crucible equipped with one layer of filter paper. The obtained solid was further washed with 90% ethanol, dried at 70 °C for 16 h, and finally weighed. The liquid phases were collected and concentrated by a rotary evaporator at 40 °C and 50 mbar until dryness. The obtained oil was added dropwise in 500 mL of Milli-Q water to promote lignin precipitation (step 3). The precipitated lignin was recovered by centrifugation at 6000 rpm (ROTOFIX 32A, Hettich) for 10 min and washed two times with Milli-Q water. The obtained lignin was dried by lyophilization (ALPHA 2-4 LD, Appropriate Technical Resources) for 24 h to yield a powder that was designated as the recovered solid and further analyzed. The recovered solids yield and mass loss of starting material were determined based on gravimetric analysis by following eq 1 below.

**Scheme 1:**
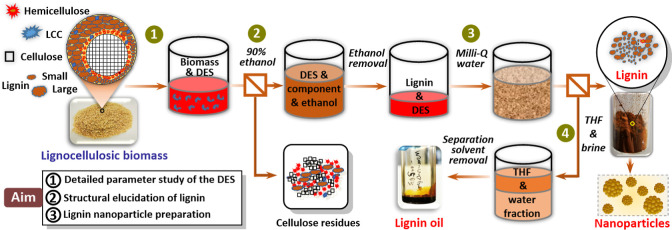
Overview of the Biomass Fractionation Process in Ternary Choline Chloride (ChCl)/Ethylene Glycol (EG)/Oxalic Acid (OA) Ternary DES System^a^

a Lignocellulose fractionation in DES at the specified conditions (step 1); 90% (v/v) aqueous ethanol addition to the cooled fractionated biomass mixture to separate the cellulose-rich residue (step 2); addition of Milli-Q water to the resulting DES–lignin mixture (after ethanol removal) to induce precipitation of the released lignin (step 3), later used for LNPs synthesis, and THF-extraction of the aqueous phase collected from the previous step to recover residual lignin fragments (step 4).

Mass loss (ML):

1 $$ML\;\left(\%\right)=\frac{starting\;biomass\;\left(g\right)-weight\;of\;residue\;\left(g\right)}{starting\;biomass\;\left(g\right)}\times 100$$

Lignin yield:

2 $$yield\;\left(\%\right)=\frac{weight\;of\;lignin\;\left(g\right)}{klason\;lignin\;\left(g\right)}$$

Next, the water phase was further extracted with THF (step 4). First, the water phase was saturated by sodium chloride, and 3 × 100 mL of THF was used to extract the phase. The THF phase was combined and dried with anhydrous magnesium sulfate. THF was removed by a rotary evaporator under vacuum to afford dark oils as the final product. The molecular weight distribution of the oils was analyzed by gel permeation chromatography (GPC). The chemical structures of the oils were analyzed by 2D Heteronuclear single quantum coherence (2D HSQC NMR) and GC–MS (see detailed description in Section 2.6.3).

### 2.4 Synthesis of LNPs via the Hydrotropic Method (DES_LNPs)

The synthesis of lignin nanoparticles via the hydrotropic method (DES_LNPs) involved the dissolution of lignin in a *p*-toluenesulfonate hydrotrope solution, followed by nanoprecipitation by water dilution. Initially, 0.21 g of lignin was added to 10 mL of a 2 M aqueous *p*TsONa solution and stirred for 12 h, resulting in a final concentration of 21 g/L. The solution was subsequently filtered using a 0.45 μm syringe filter to eliminate impurities. It was then diluted with water in a 1:3 ratio to achieve a final *p*TsONa concentration of 0.5 M. The suspension was centrifuged at 6000 rpm for 15 min, and after each centrifugation step, the solid was thoroughly washed with water to completely remove any residual *p*TsONa. The supernatant phase was discarded during this process.

This synthetic protocol was applied to DES extracted lignin obtained through extraction at various conditions: 120 °C for 2 and 4 h and 80 °C for 24 h. The same protocol was applied to a milled wood lignin (MWL) extracted via acidolysis with dioxane/HCl referred to as a benchmark.

### 2.5 Synthesis of LNPs via pH Drop-Induced Flash Precipitation (DES_FP_LNPs)

In a standard synthesis procedure, 125 mg of lignin was dissolved in 25 mL of ethylene glycol. The solution was vortexed for 30 min, filtered through a 0.45 μm syringe filter, and placed into a scintillation vial. The formation of pH drop-induced flash precipitation lignin nanoparticles (DES_FP_LNPs) was achieved by swiftly introducing 2 mL of a 0.025 M nitric acid solution into a 5 mL ethylene glycol–lignin solution that was continuously stirred. The solution was then diluted with water to stop particle growth until a final concentration of 10% ethylene glycol was reached. All the resulting particles were characterized using dynamic light scattering (DLS) and ζ-potential measurement.

### 2.6 Lignin Characterization

#### 2.6.1 Molecular Weight Measurement

Before the measurement, all the lignin samples were acetylated by slightly modifying a published method.^34^ For the acetylation, 2 mg of lignin was weighed in a 4 mL vial, followed by adding 100 μL of pyridine and 100 μL of acetic anhydride. The mixture was stirred at room temperature for 16 h in the dark, and the solvents were removed by air blowing. The dried lignin was dissolved in 1 mL of tetrahydrofuran (THF) and filtrated through a 0.22 μm PTFE filter before measurement of molecular weight (MW). MW and distribution were measured by gel permeation chromatography (GPC). A high-performance liquid chromatograph (HPLC, Shimadzu, LC-40) was equipped with a RID-20A detector and a series of two PSS SDV nonpolar columns (5 μm, 1000 Å, 8 × 300 mm^2^). THF with a flow rate of 1 mL/min was used for the eluent. Data acquisition and calculation were performed using LabSolution (GPC Postrun, 15.06.2020). Molecular weight was determined by a conventional calibration curve generated from narrow dispersity polystyrene standard from 162 to 696,000 g/mol. Samples were filtrated through a 0.2 μm PTFE filter prior to injection, and a 10 μL sample of 2 mg/mL was injected.

#### 2.6.2 2D Heteronuclear Single Quantum Coherence (2D HSQC NMR)

The 2D HSQC NMR spectra were collected from a 300 MHz instrument (Bruker BioSpin GmbH) equipped with direct probe, and a pulse sequence of “hsqcetgpsi2” was used for the ^13^C–^1^H correlation experiment. Reported parameters with minor modification were used for the analysis: spectra use 2050 data points from 8 to 0 ppm in F2 (^1^H) (acquisition time 199 ms), 160 to 0 ppm in F1 (^13^C) of 32 scans with 1 s internal delay. The total acquiring time was 2.3 h.^35^ The signal of DMSO solvent was used as an internal reference (Δ*c* 39.5, Δ*h* 2.49 ppm). The data was managed by MestReNova x64-14.2.1-27684. Volume integration of the contours in HSQC spectra was performed by MestReNova (12.0.4), and the integration was used for relative semiquantification based on the total integration of the S, G, and H signals according to the reported formula.^36^ The following formula was used for the calculation.

3 $$no.\;of\;linkages=\frac{I\alpha }{\frac{S2,6+S^{\prime }2,6+H2,6}{2}+\frac{G2+G5+G6}{2}+Sc+G^{\prime }2}$$

In the formula, *I*α is the integration of α ^13^C/^1^H of the linkages (for resinol, half of the *I*α is used); *S*2,6 is the integration of ^13^C/^1^H at position of 2 and 6 on the aromatic ring of syringyl units; *S*′2,6 is the integration of ^13^C/^1^H at position of 2 and 6 on the aromatic ring of syringyl units with an α-ketone structure; *H*2,6 is the integration of ^13^C/^1^H at position of 2 and 6 on the aromatic ring of *p*-hydroxyphenyl units; *G*2, *G*5, and *G*6 are the integration of ^13^C/^1^H at position of 2, 5, and 6 on the aromatic ring of guaiacyl units; *G*2 is the integration of ^13^C/^1^H at position of 2 on the aromatic ring of guaiacyl units with an α-ketone structure. Sc was calculated by using the integration of the total condensation area of *S*2,6. Percentage is based on 100 aromatic units with propyl (100 C9). For lignin of wheat, walnut, and bagasse, only G2 was used to represent the total G unit to avoid the signal overlapping among the H unit and *p*-coumarate and ferulate. The unit of the calculation is designated 100 C9.

#### 2.6.3 Analysis of Monomer Products via GC–MS

The crude bio-oil, obtained from THF extraction of the combined aqueous phase, was dissolved in methanol to result in a concentration of 5 mg/mL, which is suitable for GC measurements. After passing through a 0.45 μm PTFE filter, the sample was injected into a GC–MS (GCMS-QP2010, Shimadzu) equipped with an HP1-MS capillary column. The following method from published work was used for the determination: injection temperature of 280 °C, column temperature program: 2 min at 60 °C, increase to 310 °C with a heating rate of 10 K/min, detection temperature at 320 °C.^37^

#### 2.6.4 Physicochemical Characterization of LNPs

Data for DLS and ζ-potential measurements were obtained using a Malvern Z-sizer Ultra instrument with a 633 nm laser as the light source. To ensure accurate measurements, samples needed to be diluted to a minimum concentration of 23 mg/L. All samples were initially dispersed in a concentrated water solution, following the formation of nanoparticles. For DLS analysis, the water solution used was further diluted to 1 mg/mL.

## 3 Results and Discussion

We have previously demonstrated the use of ternary DES comprising choline chloride (ChCl), oxalic acid (OA), and EG for the reactive fractionation of biomass.^19,20^ While this provided proof of principle for the role of EG in suppressing recondensation phenomena, a comprehensive understanding of the fractionation parameters and their correlation with lignin structural changes was not previously studied.

Our first objective was to establish an improved lignin recovery procedure, the details of which are depicted in Scheme 1 and discussed in the Supporting Information (Section S2.1). This optimized approach allowed for lignin recovery in up to 65.5% yield from the water-precipitation step (step 3) while halving the usage of DES compared with our previous work (8:1 vs 16:1 g of DES/biomass). Next, a further THF extraction (step 4) ensured the recovery of lignin oligomers and those species with high solubility or easy suspension in water alongside with smaller organic molecules (see Section 3.4).^20^

### 3.1 Influence of Process Parameters on the Fractionation of Birch Lignocellulose in Ternary DES

Using the newly developed biomass fractionation and lignin recovery procedure, we next attempted to investigate the influence of process parameters on the lignin yield and structure. First, as profiled in Figure 2a and Tables S4 and S5, we systematically evaluated lignin fractionation at low temperature (80 °C) using the DES composition of ChCl/EG/OA over different time intervals (2, 6, 12, 24, and 72 h, Table S4). While biomass fractionation under such mild conditions is typically highly inefficient (see Section S2.2 for further discussion), interestingly in our hands, fractionation occurred at a temperature as low as 80 °C for 24 h (E8), albeit with a lower lignin recovery yield (<10%), yet with a high molecular weight of lignin (4600 Da). In addition, a high content of aryl ether moieties (combination of regular β-O-4­(OH) and modified β-O-4­(OEG)) was seen (i.e., 48.0/100 C9 vs 64.7/100 C9 for EMAL lignin, Table S5). More details on fractionation at 80 °C can be found in the Supporting Information, Tables S4 and S5.

**Figure 2:**
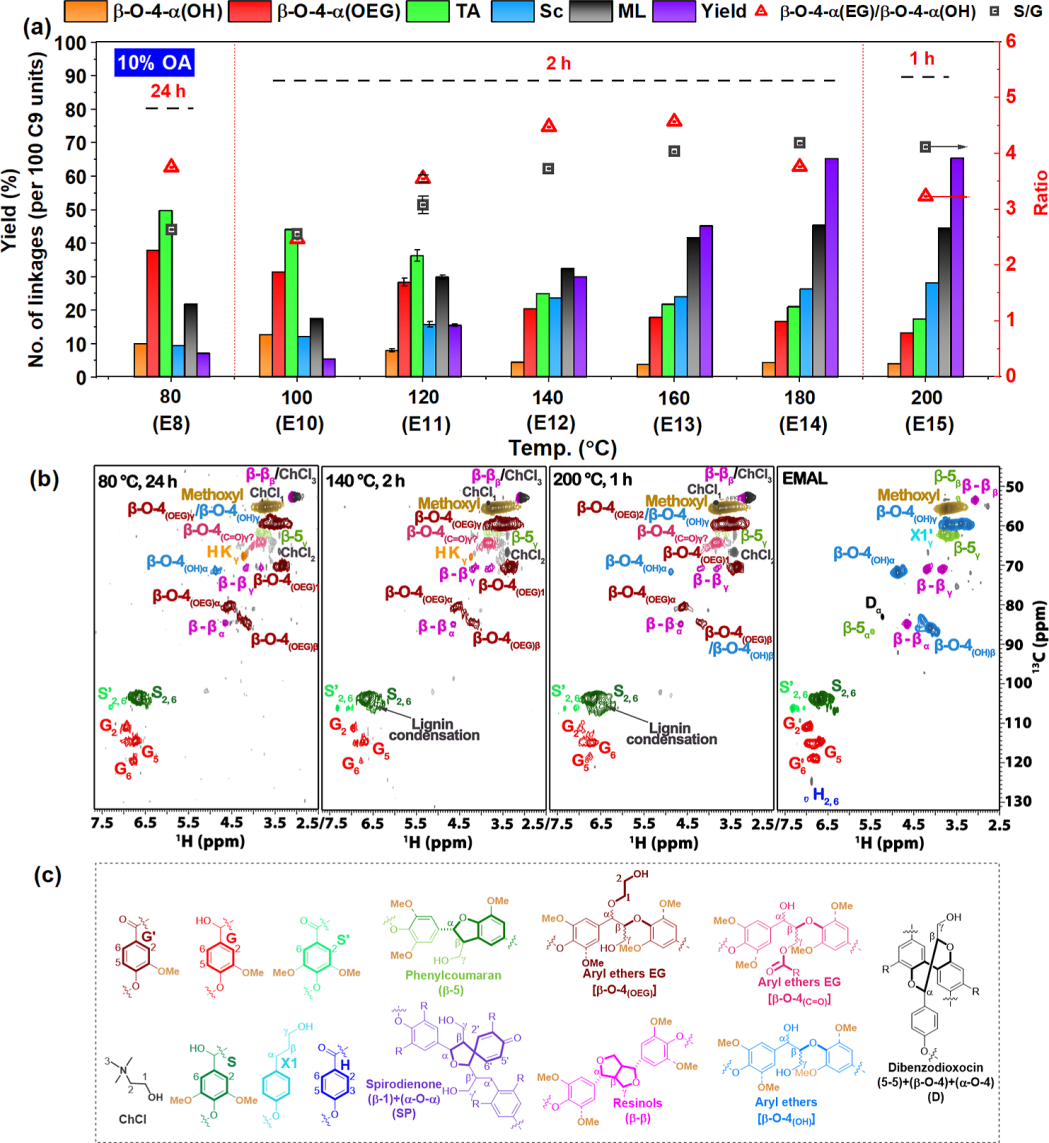
Main linkages (semiquantification from 2D HSQC NMR based on eq 3), S/G ratios, β-O-4­(OH)/β-O-4­(EG), lignin yields, obtained from treating birch biomass with ChCl/EG/OA at different times and temperatures (see detailed data and conditions in Table S6); β-O-4­(OH), aryl-alkyl ether, β-O-4­(OEG), aryl-alkyl ether with EG incorporation at the α position, β-5, phenylcoumaran, β–β, resinols, TA, total aryl ether linkage [β-O-4­(OH) + β-O-4­(OEG)]; Sc, the condensation calculated by 100 C9; ML, mass loss of starting material. (a) Reaction conditions: 16.8 g of ChCl, 14.4 g of EG, 3.12 g of OA; 4 g of birch; (b) assigned 2D HSQC NMR spectra (300 Hz, DMSO-*d*_6_) of the recovered lignin material after treatment of E8, E12, E15 and enzyme mild acidolysis (EMAL) as comparison pattern (see the original spectra in Figures S16, S21, S24, and S18, respectively). A decreasing trend in β-O-4­(OH) (light blues spots) and β-O-4­(OEG) (brown spots) signal intensities is observed with increasing temperatures, while S units signals associated with lignin degradation and condensation structures (green spots) intensify; (c) units and linkages of lignin from the EMAL and DES isolated lignin.

Next, in order to boost fractionation efficiency and lignin recovery yield, a series of systematic experiments were conducted by gradually increasing the processing temperature from 100 to 200 °C in 20 °C temperature intervals while maintaining the same ChCl/EG/OA DES composition for typically 2 h (Figure 2a, Tables S6 and S7). Interestingly, an almost linear increase (*R* = 0.99, Figure S4a) of the lignin yield from 5.5% to 65.4% (based on Klason lignin, see purple bars, Figure 2a) was observed from 100 °C (E10) to 180 °C (E14), accompanied by gradual increase of mass loss of starting birch from 17.5% to 45.5% (see black bars, Figure 2a), concluding that temperature plays a significant role in increasing lignin yield. The mass loss values indicate that at lower temperatures, delignification was not complete, while at higher temperatures, hemicellulose degradation also took place, due to the acidic nature of this DES system. Although high lignin yield was achieved at 200 °C (E15), significant structure alteration was observed even after 1 h reaction time.

The recovered lignins (E8–E15) were analyzed by semiquantitative 2D HSQC NMR spectroscopy^36^ in order to understand structural modifications, including the distribution of various linking motives, as well as the extent of EG incorporation as a function of temperature. Representative regions of the relevant spectra and linkages are depicted in Figure 2b,c while linkage analysis is summarized numerically in Figure 2a and Table S7.

The total content of β-O-4 linkages (TA, green bars, Figure 2a,b) gradually decreased from 49.8/100 C9 at 80 °C (E8) to 44.2/100 C9 at 100 °C (E10) and then to 17.5/100 C9 at 200 °C (E15); it is however remarkable that even at 200 °C (E15), a relatively good retention of β-O-4 moieties was seen. To express the extent of EG incorporation, the ratio between the β-O-4 linking motif with and without benzylic substitution is depicted (β-O-4-α­(EG)/β-O-4-α­(OH), red triangle, Figure 2a). This value appears to increase with fractionation temperature to 4.5 (E12) at 140 °C and 4.6 (E13) at 160 °C while dropping to 3.8 (E14) at 180 °C and 3.2 (E15) at 200 °C. These experiments point to an optimum temperature range 140–160 °C for EG incorporation. Furthermore, gradually increasing condensation of aromatic units was seen with increasing temperature, reaching 28.2/100 C9 at 200 °C (E15). Accordingly, the ratio of S/G increased from 2.6 (E8) to 4.2 (E15).

Next, the effect of fractionation time was systematically investigated at 120 °C for 2, 4, 6, and 12 h as well as at 140 °C for 2, 6, and 12 h. Results are summarized in Figure 3 and Tables S8 and S9. Overall, surprisingly, prolonging fractionation time did not significantly affect the recovered lignin yield (compare 15.5% at 2 h versus 23.4% at 12 h). However, prolonged DES treatment led to gradual and significant increase of EG incorporation (red bars) and slight structural alteration, as evidenced by a changed S/G ratio (square symbols) and decrease of MW (Figure 3b). Gratifyingly, there was no significant condensation even at prolonged fractionation times at these reaction temperatures (Figure 3a), as a result of the protective effect of the EG incorporation, in accordance with previously reported data.^20^

**Figure 3:**
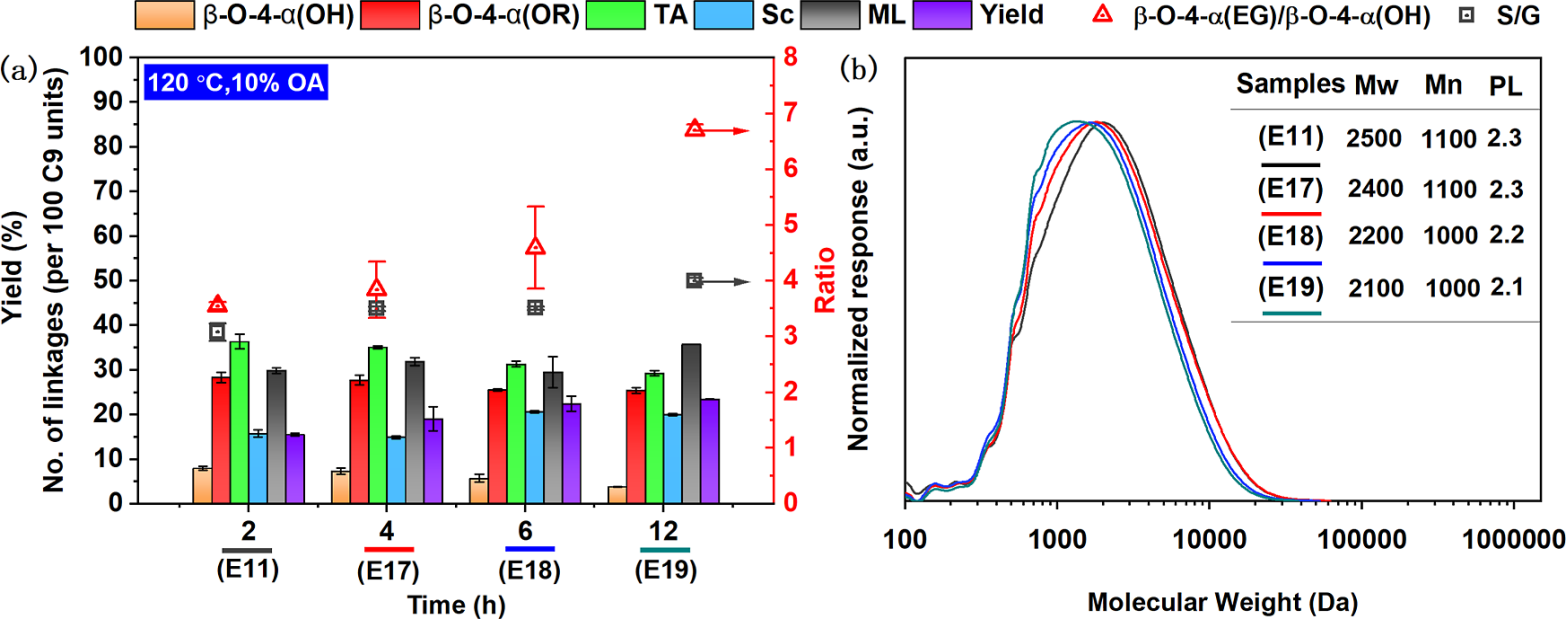
Main linkages (2D HSQC NMR based on eq 3), S/G ratios, β-O-4­(OH)/β-O-4­(EG), and lignin yields, obtained from treating birch biomass with ChCl/EG/OA at 120 °C (see detailed data and conditions in Table S8); β-O-4­(OH), aryl-alkyl ether, β-O-4­(OEG), aryl-alkyl ether with EG incorporation at the α position, β-5, phenylcoumaran, β–β, resinols, TA, total aryl ether linkage [β-O-4­(OH) + β-O-4­(OEG)]; Sc, the condensation calculated by 100 C9; ML, mass loss of starting material. (a) Reaction conditions: 16.8 g of ChCl, 14.4 g of EG, 3.12 g of OA; 4 g of birch; (b) molecular weight mass of lignin correlated with (a).

### 3.2 Influence of DES Constituents

Our investigated ternary DES combines ChCl as HBA with oxalic acid (OA) and ethylene glycol (EG) as HBD, playing diverse key roles in the fractionation process. Oxalic acid has been previously identified to be efficient in promoting the release of lignin from the cell wall, thus facilitating lignocellulose fractionation.^38,39^ However, pulping under acidic conditions affects the β-O-4 moieties via acid-mediated dehydration of the secondary alcohol, followed by the formation of relatively stable benzylic carbocations that are prone to recondensation reactions and/or further cleavage.^40,41^ As we have previously noted, the beneficial role of EG is to suppress these undesired recondensation reactions, maintaining the β-O-4 moieties and ultimately resulting in EG decorated lignin with increased stability against degradation. Lignins so obtained display altered properties compared to typical organosolv lignin.^19,20,42^

In order to study the influence of DES components, four different DES compositions and 2 OA/EG mixtures were prepared and tested. Results obtained are summarized in Figure 4a and b. When DES composition prepared only from ChCl and OA was used at 120 °C for 4 h (E22), the lignin obtained displayed a low number of β-O-4 linkages and lower molecular weight (1200 Da), indicating the key role of EG in DES for protecting the structural integrity of lignin. Without EG stabilization, the lignin underwent condensation and partial depolymerization, consistent with our previous findings and literature.^19,43^

**Figure 4:**
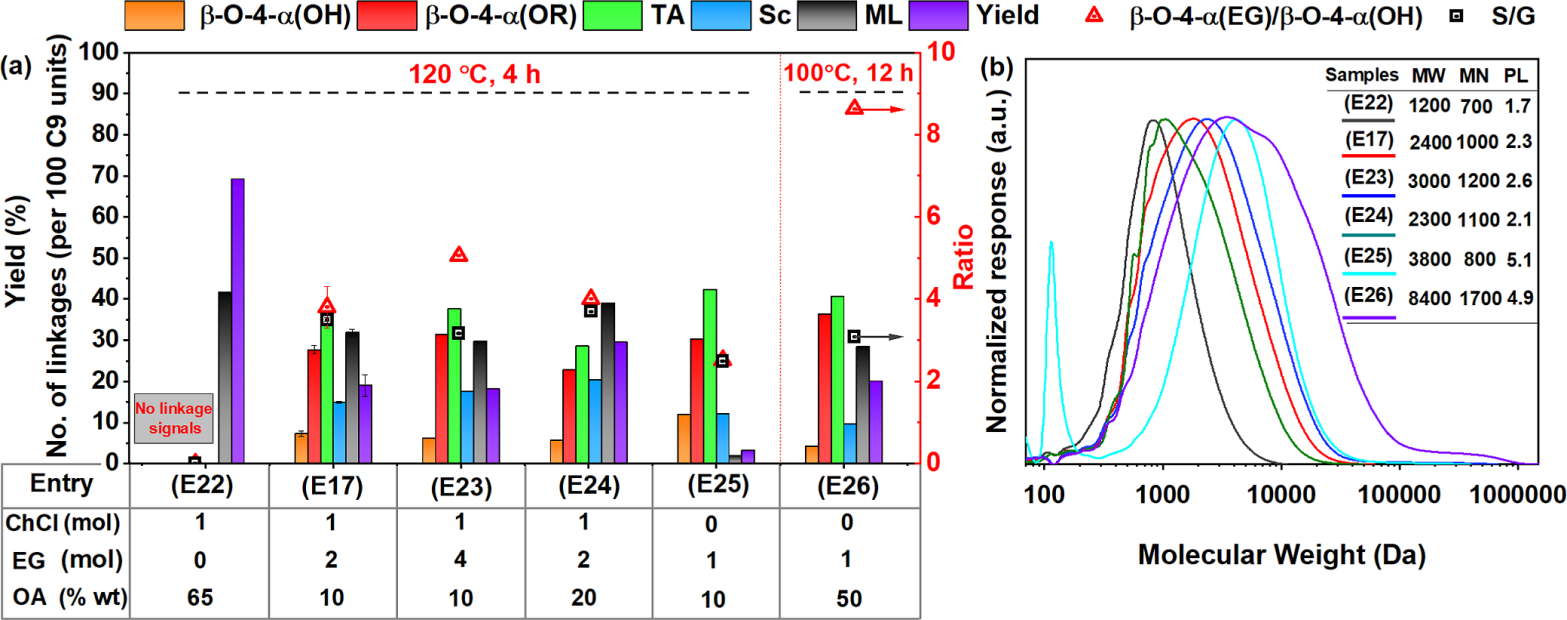
Main linkages (semiquantification per 2D HSQC NMR based on eq 3), S/G ratios, β-O-4­(OH)/β-O-4­(EG), and lignin yields, obtained from treating birch biomass with different DES compositions. (a) Reaction conditions: 4 g of birch at 120 °C for 4 h (E17, E22–E25) and 100 °C for 12 h (E26, see detailed data and conditions in SI); β-O-4­(OH), aryl-alkyl ether, β-O-4­(OEG), aryl-alkyl ether with EG incorporation at the α position, β-5, phenylcoumaran, β–β, resinols, TA, total aryl ether linkage [β-O-4­(OH) + β-O-4­(OEG)]; Sc, the condensation calculated by 100 C9; ML, main mass loss of starting material, yield and detailed structural information calculated from 2D HSQC NMR (the error is less than 5%). (b) MW distribution of the lignins for (a).

Next, the variation of EG loading was studied by conducting experiments using ChCl/EG/OA (1:2 mol ratio, 10 wt %) versus ChCl/EG/OA (1:4 mol ratio, 10 wt %) (E17 versus E23 respectively; see detailed data in Tables S8–S11). Increasing the EG loading enhanced the ratio of EG incorporation from 3.8 to 5.1. This was also clearly revealed by the molecular weight of the obtained lignin (2400 vs 3000 Da, MW, Figure 3b). Increasing the OA loading from 10% (E17) to 20% (E24) significantly increased the lignin yield from 19.0% to 29.6%. However, loss of aryl ether linkages (green bar in Figure 4) and increased condensation were observed (blue bar in Figure 4), consistent with the higher acidity of the reaction medium. The β-O-4 aryl ether linkage in lignin is known to be acid sensitive.^19^ Thus, increasing the OA loading led to higher lignin release from the biomass, but at the same time, it caused β-O-4 bond degradation, leading to reduced structural preservation. When mass loss is compared, it appears that the degradation of carbohydrate fractions was also more prominent at higher acidity, as expected.

In order to further confirm the role of the original ternary DES system, two separate fractionations without ChCl were conducted at 120 and 100 °C and for 4 and 12 h, respectively (E25, E26). In these instances, fractionation takes place in EG as a solvent, and acidity is controlled by the amount of OA. As expected, in the absence of a true DES solvent, the lignin recovery yield stays low (3.3%) at 120 °C, as evidenced by the E25 conducted in EG with 3.12 g of OA added. A further increase of OA loading and prolongation of fractionation time to 12 h (E26) at 100 °C increased the lignin yield to 20.1%. These data further indicate that OA promoted the release of lignin from the biomass. Intriguingly, the lignins obtained in EG as a solvent displayed markedly different Mw values depending on reaction conditions. For instance, the MW was 3800, 8400, and 2400 Da for E25, E26, and E17, respectively. This may open the possibility of using this solvent for isolating lignin with different MW.

Furthermore, we have also investigated the effect of the biomass particle size on fractionation efficiency. To this end, we have performed ball milling of birch according to our previously reported protocol and collected birch lignocellulose of different size ranges by sieving.^44^ The results are summarized in Section S2.3 (Figure S6, Tables S14 and S15) and show that ball milling had a positive effect on the lignin recovery yield.

Finally, we also evaluated the effect of the controlled addition of water (up to 10%) into the DES prior to biomass fractionation. It is interesting to note that increasing the DES water content also has a positive effect on lignin recovery yield, likely due to more efficient removal of hemicellulose-derived sugars from the cell wall. These results are also summarized in Supporting Information Section S2.3.

### 3.3 Extending the DES Extraction to Various Lignocellulose Species

Lignocellulosic biomass displays a broad compositional and structural variation depending on the plant species, geographical locations, and environmental factors.^14,15,45^ Therefore, we evaluated our DES fractionation method across various lignocellulose sources. Besides birch, other biomasses including pine wood, wheat, walnut shell, and bagasse from sugar cane were selected to represent softwood, grass, and biomass-residues, respectively. The results are summarized in Figure 5 and Tables S12 and S13. All fractionations were conducted at 140 °C for 2 h, earlier established for birch lignocellulose as optimal conditions for good lignin recovery yield and high β-O-4 retention. Overall, the different biomasses have shown markedly different lignin yields, as expected, due to their dissimilar lignin content. Lignin isolated from wheat straw (E28, Table S12) had the highest yield (44.8%), far surpassing that obtained from pine wood (E27, 21.0%) under the same conditions. Furthermore, lignin was also isolated from other biomasses such as walnut shell (E29, 40%) and bagasse (E30, 21.6%). As indicated by the 2D HSQC NMR spectrum in Figure 5, typical signals for the lignin from different biomass sources were observed.

**Figure 5:**
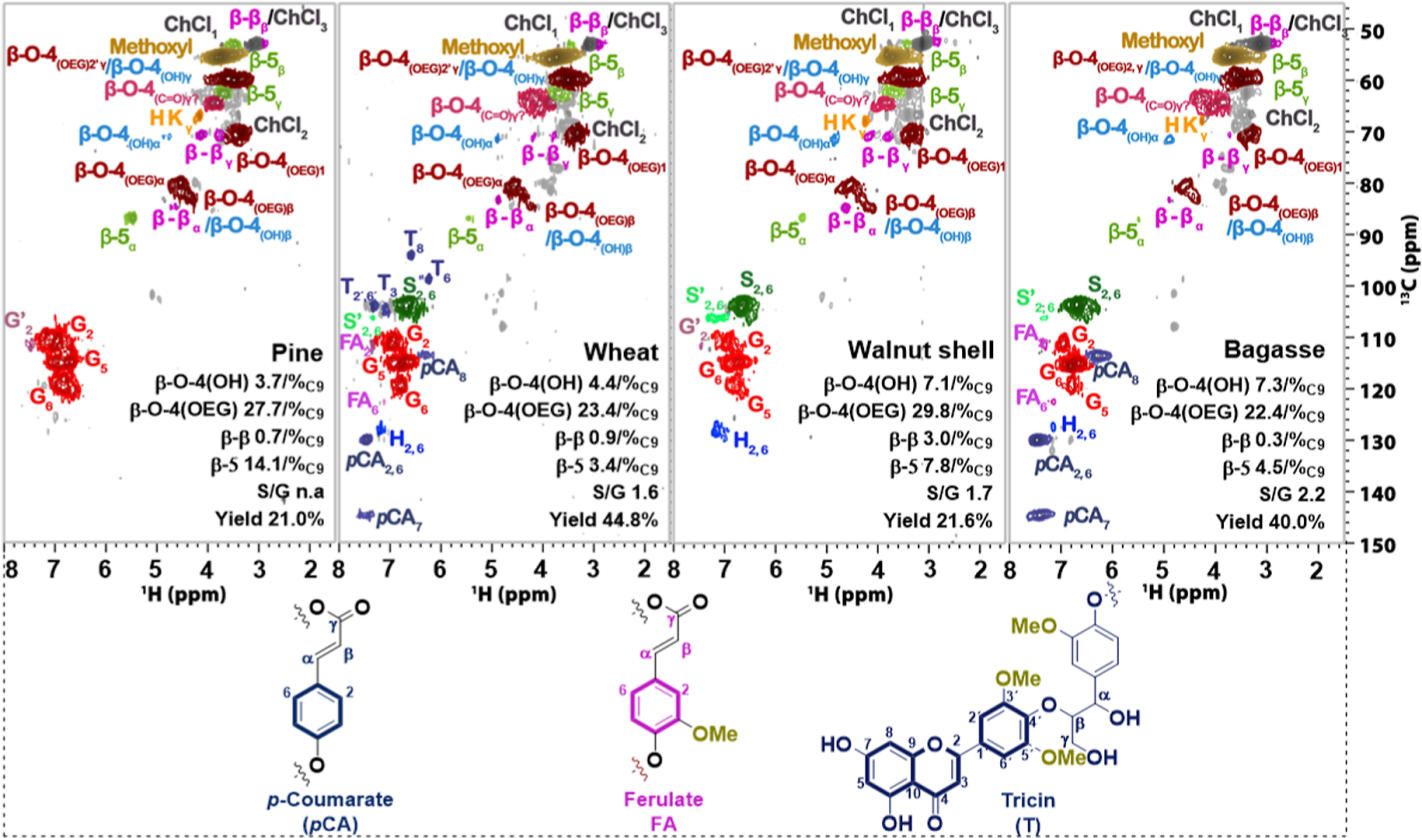
2D HSQC NMR (300 Hz, DMSO-*d*_6_) linkages of lignins obtained from treating different biomass with ChCl/EG/OA, β-O-4­(OH), aryl-alkyl ether, β-O-4­(OEG), aryl-alkyl ether with EG incorporation at the α position, β-5, phenylcoumaran, β–β, resinols; reaction conditions: 16.8 g of ChCl, 14.4 g of EG, 3.12 g of OA, 4 g of lignocellulose 140 °C for 2 h (the error is less than 5%). Guaiacyl units (red spots) predominant in pine lignin, Tricin, a flavone unit of lignin, was clearly found in the wheat lignin (blue spots). *p*CA (dark blue spots) and FA (pink spots) are typical units for grass biomass and can be clearly observed for both wheat and bagasse lignin. Walnut lignin showed *p*-hydroxyphenyl units (blue spots, H).

In terms of aryl ether linkages, under the same DES fractionation, lignin from the walnut shell (E29) still contained 36.9/100 C9 β-O-4 content, more than that achieved with birch 25.0/100 C9 (E12). The β-O-4 content of pine lignin was slightly higher than that of lignin obtained from sugar cane bagasse. However, the highest level of EG incorporation (7.4%) was found for lignin from pine.

This trend was also highlighted by the MW (Figure S5d), EG incorporation, and condensation (Table S13), likely due to the different proportions of S, G, and H units that are closely linked with the stability of the aryl ether linkage and reactivity of the benzylic hydroxy groups. For example, hardwoods such as walnut shell and birch, which are enriched in syringyl (S) units, generally show higher β-O-4 preservation, whereas guaiacyl (G)-rich softwoods such as pine are more condensed and therefore yield lower amounts of lignin with fewer intact β-O-4 linkages. Grasses such as wheat straw and bagasse display additional hydroxycinnamate-derived units (e.g., *p*-coumarates, ferulates, and tricin), which influence both solubilization efficiency and linkage stability. These differences highlight the need for further in-depth optimization of lignin fractionation via this DES in order to maximize the isolation of lignin and conservation of valuable linkages for different lignocelluloses.^46–49^

### 3.4 Analysis of the Residual Organic Extracts Obtained from the Aqueous Phase

Lastly, we have investigated the residual lignocellulose-derived products remaining in the aqueous phase, postfractionation, and lignin precipitation. As outlined in Scheme 1, our lignin-recovery procedure consisted of the dropwise addition of the concentrated lignin-liqueur into water, resulting in recovery of high-purity target lignin upon filtration (step 3 in Scheme 1). To understand the nature of residual, water-soluble products, we extracted the aqueous phases from a range of fractionation experiments using THF, followed by 2D HSQC NMR, GPC, as well as GC–MS analysis after THF removal. The detailed description of these experiments can be found in Section S2.4.

As expected, the aqueous phases contained water-soluble species ranging from hemicellulose, sugar derivatives, aromatic monomers, EG-functionalized lignin oligomer fragments, and even highly condensed lignin fragments, depending on fractionation temperature. Low-molecular-weight lignin fragments generated during fractionation exhibit increased polarity due to a higher content of phenolic hydroxyl groups and ethylene glycol incorporation, which enhances their solubility in water during DES quenching and washing steps. Interestingly, DES fractionation under milder conditions (80–100 °C) enabled the almost selective extraction of hemicellulose and derivatives into the aqueous phase, as also evidenced by the high MW (>1000 Da, Figure S9) as well as the presence of carbohydrate-relevant and lack of aromatic signals in 2D HSQC NMR (Figure 6). In contrast, typical aromatic signals ascribed to lignins and derived oligomers were observed, resulting from fractionation above 120 °C, with significant structural differences depending on processing conditions. For example, at around 120 °C, more pronounced EG incorporation was seen, while above 200 °C, significant condensation was seen.

**Figure 6:**
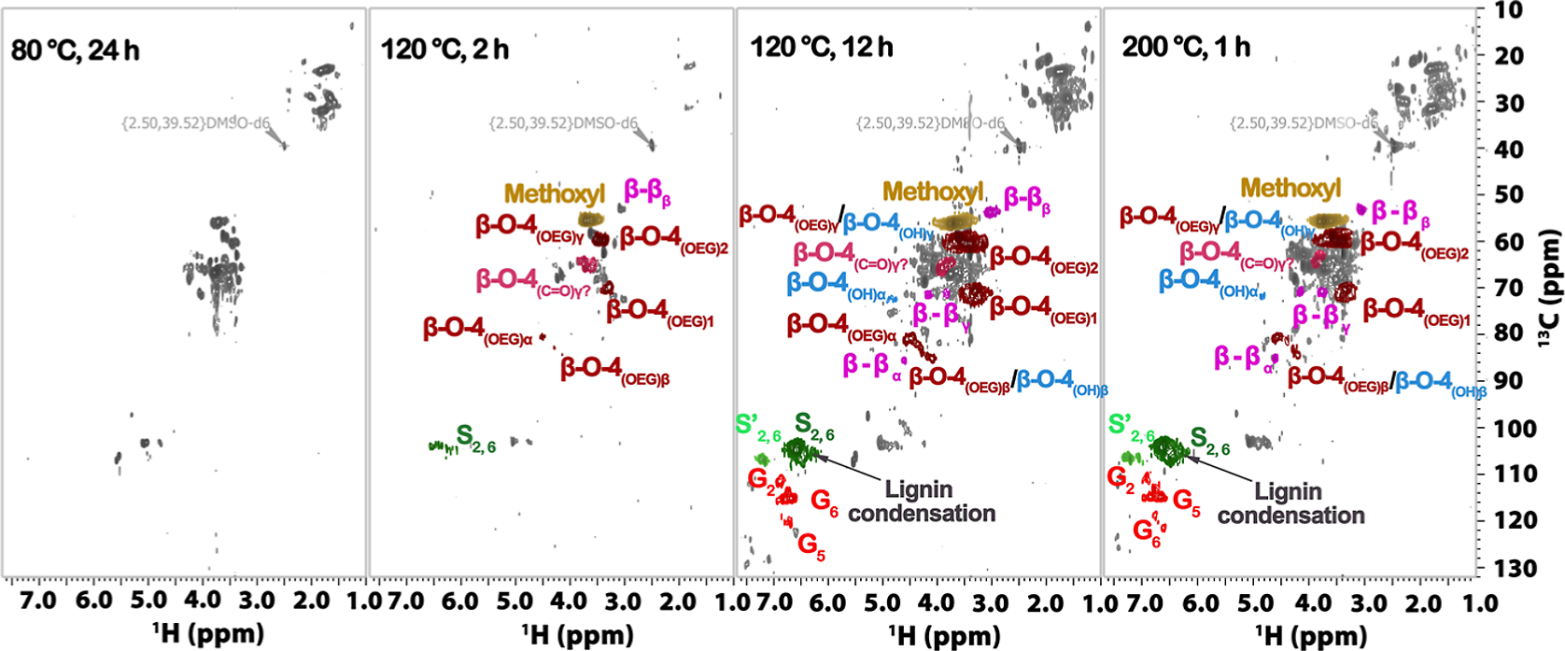
2D HSQC NMR spectra (300 Hz, DMSO-*d*_6_) of the oil obtained from the THF extraction of the liquid phase collected from DES treatment of birch with ChCl/EG/OA; here, it can be seen that at mild conditions (80 °C/24 h), products primarily derived from carbohydrates (green spots) are detected. As the temperature increases beyond 120 °C, lignin signals (β-O-4­(OH), β-O-4­(OEG), β–β) become prominent, with their intensity rising as fractionation time extends from 2 to 12 h. More severe conditions, such as 160 and 200 °C, lead to increased lignin condensation (S, G units); reaction conditions: 16.8 g of ChCl, 14.4 g of EG, 3.12 g of OA, 4 g of birch, 80 °C/24 h, 120 °C/2 and 12 h 200 °C/1 h. β-O-4­(OH), aryl-alkyl ether, β-O-4­(OEG), aryl-alkyl ether with EG incorporation at the α position, β–β, resinols.

The detailed GC–MS analysis of the volatile, monomeric range of the THF extracts (Figures S1, S10, and S11) has revealed weak signals of monophenolic compounds consistent with lignin depolymerization via acidolysis and diol-assisted fractionation as well as signals originating from (hemi)­cellulose degradation in the presence of EG.^13,42,43,50–54^ These reveal that, especially at higher fractionation temperatures, next to the main diol-mediated EG protection of lignin, some acid-mediated lignin fragmentation is also a viable pathway in the used ternary DES systems. These aromatic monomers do not coprecipitate with the lignin but remain in the aqueous phase during the lignin isolation protocol.

Overall, it can be concluded that the fractionation and lignin precipitation/purification protocol developed herein results in well-defined lignins in high purity and good yield under optimized conditions; however, some material loss into the aqueous phase, especially through EG incorporation or fragmentation, cannot be fully prevented.

### 3.5 Colloidal Dispersions from Lignin Isolated by ChCl/EG/OA Extraction

Recent trends in lignin valorization point toward the production of colloidal dispersion of LNPs as possible high-added-value components for nanocomposites.^11,55–60^ With the objective of valorizing the DES-extracted lignin in a possible one-pot process toward colloidal lignin dispersions, we first evaluated the tendency of DES lignin to self-assemble. Since the main modification of the lignin backbone induced by the reactive DES extraction involves the introduction of the hydroxy-ethoxy moiety in the α-position of the β-O-4 aryl ether bond, it is of pivotal importance to identify the effect of the overall hydrophobicity change on the propensity of lignin to aggregate.^61^ In this perspective, LNPs were synthesized via two different strategies involving a solvent–antisolvent approach. More specifically, LNPs were first obtained by dissolution in a *p*-toluenesulfonate hydrotropic solution followed by water-mediated nanoprecipitation.^62,63^

Alternatively, in the pH-drop-induced flash precipitation method, a colloidal system is established by rapidly decreasing the pH through the addition of a nitric acid solution to an ethylene glycol (EG)/lignin solution. This pH drop associated with the antisolvent behavior of water triggers the supersaturation of DES lignin, followed by nucleation and particle growth.^64,65^

The LNPs obtained were compared with analogous colloidal suspensions as synthesized from dioxane lignin (synthesized from a benchmark lignin extracted via acidolysis with dioxane/HCl). These particles exhibit a hydrodynamic radius within the submicrometer range (Figure 7). DES_LNPs were found to be slightly bigger than the corresponding dioxane_LNPs. Efforts to conduct reproducible analyses on larger aggregates synthesized via the hydrotropic method, using DES lignins as a starting material, faced limitations due to instrument sensitivity above 1 μm. The presence of these aggregates is ascribable to the low colloidal stability of the primary nanoparticles, which coalesce to form bigger aggregates. Additionally, the solutions appear cloudy, indicating the presence of particles comparable to or larger than 400–700 nm. DES nanoparticle solutions remain stable for up to 8 h, after which sedimentation occurs. After one month, the supernatant becomes transparent and colorless.

**Figure 7:**
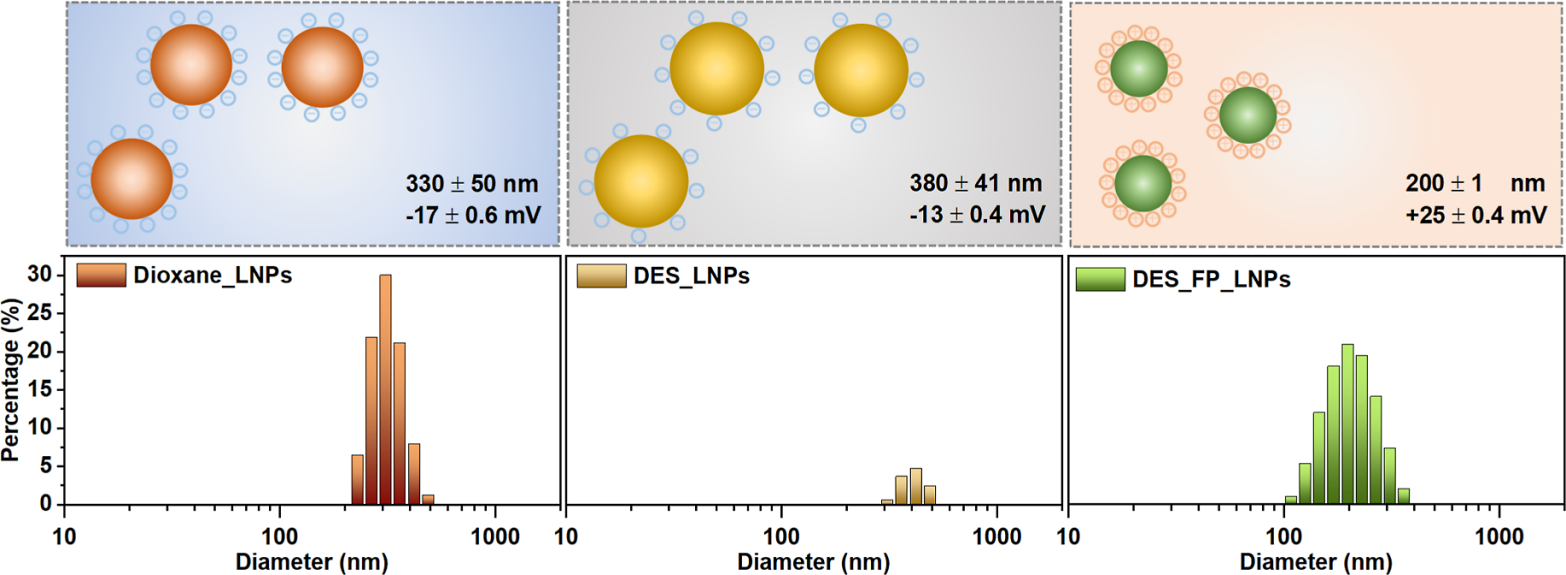
LNPs parameters obtained from DLS measurements, covering a size range of 1–1000 nm, as well as the zeta potential. Values are reported as mean ± standard deviation (SD) based on *n* = 3 independent measurements. The hydrodynamic radius is based on the first peak. The hydrodynamic radius and zeta potential values are provided for nanoparticles synthesized using lignin extracted with dioxane (dioxane_LNPs; see detailed lignin extraction procedure in Section S1.4) and lignin extracted with the DES method under conditions of 120 °C and 2 h, using both the hydrotropic method (DES_LNPs) and the pH-induced method (DES_FP_LNPs). For the lignin DES benchmark, only the values related to the hydrotropic method are included, as the pH-induced flash precipitation method did not result in particle formation.

Dioxane_LNPs display hydrodynamic radius and zeta potential values akin to those from DES-extracted lignin at 120 °C for 4 h (ChCl:EG:OAm 1:2:0.3 molar ratios). However, the turbid orange colloidal suspension of dioxane_LNPs remains stable for up to three months. Encouraging outcomes emerged from the pH-induced flash precipitation method, yielding particles with a radius of 200 nm and a zeta potential of 25 mV falling within the stability range of the nanoparticles. Solutions derived from lignin DES (120 °C, 2 h, ChCl:EG:OAm 1:2:0.3 molar ratios) are light brown and transparent.

The stabilization of lignin nanoparticles derived from DES-extracted lignins is closely linked to both the structural modifications introduced during DES fractionation and the nanoparticle formation pathway. It is likely that ethylene glycol incorporation at the benzylic position of β-O-4 linkages likely increases lignin polarity and reduces the intermolecular π–π stacking, while the lower degree of condensation favors controlled self-assembly rather than irreversible aggregation.^66^ In the hydrotropic method, gradual nanoparticle formation occurs upon dilution from a solubilized state, whereas pH-induced flash precipitation generates rapid supersaturation and enhanced electrostatic repulsion, leading to smaller and more stable nanoparticles, as reflected by their ζ-potential values.^63^

In short, this study demonstrates the successful production of lignin nanoparticles through two distinct methods: hydrotropic precipitation and pH-induced flash precipitation. Dynamic light scattering (DLS) analysis indicated a unimodal distribution of nanoparticles, with the hydrodynamic radius influenced by the degree of lignin functionalization. Zeta potential measurements provided valuable insights, highlighting the differences between the two methods and two types of lignin. Specifically, nanoparticles produced via the hydrotropic process using DES lignin exhibited a propensity to aggregate and displayed instability, whereas those synthesized through the pH-induced flash precipitation method were smaller in size and maintained stability throughout this study.

## 4 Conclusion

In this work, a systematic study on lignin isolation from diol-based DES was conducted. Lignin with tunable properties, such as the content of aryl ether linkage and MW, was obtained by tailoring the constitution of DES and changing the fractionation conditions. Among them, DESs prepared with a high EG/ChCl, higher than 2, isolate lignin with a better preservation of aryl ether linkage and high MW. Relatively high temperatures (160–180 °C) combined with long retention times (2–6 h) resulted in higher lignin yields, whereas moderate temperatures (80–120 °C) and high EG/ChCl ratios (≥2) promoted the isolation of lignin with a higher preservation of aryl ether linkages. These structural features directly translated into improved self-assembly behavior and enhanced colloidal stability of DES_LNPs with a radius of 200 nm and a zeta potential of 25 mV when prepared via pH-induced flash precipitation (DES_FP_LNPs). Moreover, it was convincingly demonstrated that the method can be extended to a range of different biomass types, although the lignin isolated varied from different biomasses. The versatility of LNPs obtained from DES lignin paves the way for the development of a one-pot procedure for the direct LNP synthesis from wood. Overall, this study shows that diol-based ternary DESs have a unique performance for isolating lignin with tailored properties, and the data presented in this work provide valuable insights into understanding lignin structural alteration under different conditions.

## Supplementary Material



## Notes

Supplementary experimental details, results and discussion, raw 2D HSQC NMR spectra, GPC analysis, mass spectra of the identified compounds, and other supplementary tables and figures (PDF)
