# Supplementary material for: Alcohol-Decorated Lignins for Nanoparticle Formation through Reactive Fractionation in Ternary Deep Eutectic Solvent Systems
Source: ACS Sustain Chem Eng. 2026 Feb 19;14(8):3814–25. doi: 10.1021/acssuschemeng.5c07102 (PMC12958331; doi:10.1021/acssuschemeng.5c07102)
Supplement: Supplementary file 1 [file sc5c07102_si_001.pdf]

Supporting information

Alcohol-decorated lignins for nanoparticle formation through reactive fractionation in ternary deep eutectic solvent systems

Zhiwen Wang[†‡], Danelon Umberto[§], Roberto Sole†, Claudia Crestini[§], Katalin Barta[†]**

[‡]College of Forestry, Northwest A&F University, Yangling, 712100, China,

[†]Department of Chemistry, Organic and Bioorganic Chemistry, University of Graz, Heinrichstrasse 28/II, 8010 Graz, Austria

[§]Department of Molecular Sciences and Nanosystems, Ca' Foscari University of Venice, Via Torino, 155, 30172, Mestre (VE) Italy

Index

S1. Supplementary experiment sections 2

S1.1. Yield correction for alcohol incorporation 2

S1.2. Correction of molecular weight distribution for acetylation and alcohol incorporation 2

S1.3. Isolation of enzyme mild acidolysis lignin (EMAL) 3

S1.4. Isolation of dioxane lignin 3

S1.5. Composition analysis: 4

S2. Supplementary results and discussion 4

S2.1. Method development: recovery of lignin from DES fractionation 4

S2.2. Lignin isolation from ChCl/EG/OA: fractionation under mild conditions 5

S2.3. Lignin isolation from ChCl/EG/OA: the influence of water addition and particle size 6

S2.4. Analysis of the organic extracts from water precipitation to detect lignin oligomers and monomeric fragments 7

S3. Supplementary figures 10

Supplementary tables 60

References 70

S1. Supplementary experiment sections

S1.1. Yield correction for alcohol incorporation

The yield was corrected after consideration of the ethylene glycol incorporation and a correction factor is calculated. The mass of the monolignols with and without ethylene glycol incorporation is attached below.

Table S1

Mass of the monolignols with and without ethylene glycol

Units	No incorporation		With incorporation		[a] Correction factor
S	226.08	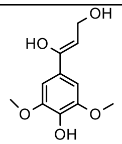	270.11	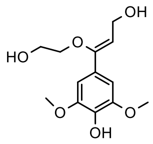	0.19
G	196.07	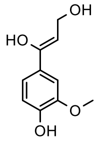	240.10	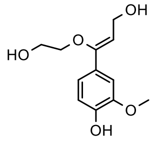	0.22
H	166.06	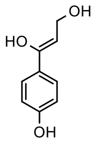	210.09	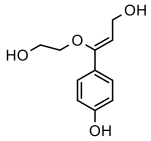	0.27

[a] The correction factor = $\frac{Mass\ [\beta{-}O{-}4(EG)]}{Mass\ [\beta{-}O{-}4(OH)]} - 1$

Based on the result of S, G and H units from 2D HSQC, the corrected yield can be calculated with the following equations:

Corrected yield of lignin (mg)= $\text{experimental yield (mg)} * (1 - \frac{\beta{-}O{-}4(EG)}{100} * (0.19 * fraction\ S + 0.22 * fraction\ G + 0.27 * fraction\ H))$

S1.2. Correction of molecular weight distribution for acetylation and alcohol incorporation

The molecular weight was corrected after consideration of the ethylene glycol incorporation and acetylation, and a correction factor is calculated. The mass of the monolignols with and without ethylene glycol incorporation as well as acetylation is attached below.

Table S2

Mass of the monolignol with and without ethylene glycol and acetylation

Units	No incorporation	No incorporation+acetyl	[a] Cf for β-O-4(OH)	With incorporation+acetyl	[b] Cf for β-O-4(OEG)	β-5	[c] Cf for β-5
S	226.08 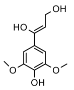	310.11 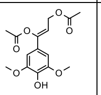	0.37	354.13 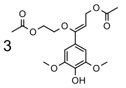	0.57	268.09 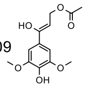	0.19
G	196.07 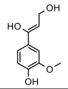	280.09 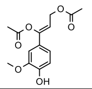	0.43	324.12 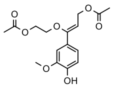	0.65	238.08 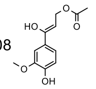	0.21
H	166.06 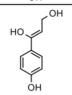	250.08 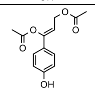	0.51	294.11 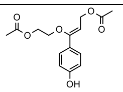	0.77	208.07 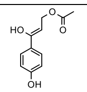	0.25

[a] The correction factor = $\frac{Mass\ [\beta{-}O{-}4(OA)]}{Mass\ [\beta{-}O{-}4(OH)]} - 1$

[b] The correction factor = $\frac{Mass\ [\beta{-}O{-}4(OEGA)]}{Mass\ [\beta{-}O{-}4(OH)]} - 1$

[c] The correction factor = $\frac{Mass\ [\beta{-}5(OA)]}{Mass\ [\beta{-}O{-}4(OH)]} - 1$

Based on the result of S, G and H units from 2D HSQC, the corrected yield can be calculated with the following equations:

Corrected mass of lignin (D)= $\text{experimental mass (D)} * (1 - \frac{\beta{-}O{-}4(OH)}{100} * (0.37 * fraction\ S + 0.43 * fraction\ G + 0.51 * fraction\ H) - \frac{\beta{-}O{-}4(EG)}{100} * (0.57 * fraction\ S + 0.65 * fraction\ G + 0.77 * fraction\ H) - \frac{\beta{-}5}{100} * (0.19 * fraction\ S + 0.21 * fraction\ G + 0.25 * fraction\ H))$

S1.3. Isolation of enzyme mild acidolysis lignin (EMAL)

The EMAL was isolated by following steps from published procedures. 20 g biomass with 6 h ball milling was mixted 500 ml sodium acetate (50 mM, Ph=5.5), and 10 mL fresh Cetec2 was added drop by drop under vigorous stirring in a 1000 mL scale of bottles. The sample was incubated under 50°C, 250 rpm shaking for 72 hours. After incubation, the solid residue was separated by centrifugation and flowed by Milli-Q water washing and freeze-drying. 10 g residue was mixed with 400 mL 1,4-dioxane and 1.42 mL 35% HCl. The mixture was put in an oil bath (preheated at 86°C) for 2 hours, and separated by filtrating through a filter paper. The liquid fraction was neutralized by $NaHCO_3$ and then condensed by rotary rap. Lignin was recovered by precipitating the condensed liquid in acid water (pH=2), and separated by centrifugation. The final lignin was further washed by acid water and dried in lyophilier (ALPHA 2-4 LD, Appropriate Technical Resources).

S1.4. Isolation of dioxane lignin

Milled wood lignins are isolated by the original method described by Pepper. Dewaxed milled wood was stirred with *p*-dioxane: water (9:1)/HCl resulting in the dissolution of crude milled wood lignin. The resulting dispersion was filtered off and the solvent evaporated from the filtrate under reduced pressure.

A three necked round bottom flask equipped with a condenser, an N_2 valve and a dripping funnel, was filled with 10 grams of wiley milled wood and the whole system is vacuum packed for at least 15 mins. Pour 80 ml of 9:1 solution of dioxane: water containing 0,58 g of dry HCl gas in the dropping funnel with a final molarity of 0,2 M, and subsequently added slowly to the biomass. The reaction mixture has been heated with an oil bath and refluxed gently for 1 h under N_2 atmosphere and constant magnetic stirring.

After 1h the reaction was allowed to cool, filtered whilst blanketed with nitrogen, and the residual washed on the filter with a further 50 ml of the same solvent. Cellulose and sugar residues were dried to constant weight to determine the yield. The filtrate was neutralized with excess of sodium bicarbonate and allowed to stand overnight. The liquid layer was concentrated under reduced pressure, using a nitrogen leak to a thick syrupy consistency, dissolved in ca. 30 mL of solvent (dioxane: water 9:1) and added slowly dropwise on the surface of 250mL of a 1% Na_2SO_4 solution. The precipitated lignin was separated by centrifugation and decantation, washed thoroughly with water, and centrifuged three times (5000 rpm for 15 min) and finally freeze dried to constant weight. Lignin isolated was a light tan colored powder, slightly hygroscopic and highly susceptible to static electricity when dry.

S1.5. Composition analysis:

The carbohydrate composition analysis followed the NREL method. A 0.3 g scale was used for raw material analysis, while lignin and pretreated biomass samples were analyzed on a 0.1 g scale. Samples were hydrolyzed by 72% sulfuric acid at 30 °C for 1 h and further diluted to 4% at 121°C for another 1 h. In addition, the sugar degradation during the two steps acid hydrolysis was corrected by their corresponding monosaccharides. The concentration of monosaccharides in the resulting solution was determined by HPLC (1200 Agilent Technologies, USA) with a refractive index detector (RID). An aminex column HPX-87H (Bio37 Rad, USA) was used to separate the monosaccharides at 50°C with 5 mM sulfuric acid at a flow rate of 0.6 mL/min. Under these conditions, xylose, mannose and galactose was eluted at the same retention time which were integrated at a single peak. Since xylan is the main constituent and the calibration constant among these three monosaccharides was not very big. Thus, that overlapped peak was directly quantified by the calibration curve of xylose, which is similar to a reported method (Ferraz et al., 2000) and the hemicellulose of the samples was represent as the total amount of xylan, mannan, galactan and arabinan.

S2. Supplementary results and discussion

S2.1. Method development: recovery of lignin from DES fractionation

The yield of lignin recovered from the fractionation process is closely linked to the method used for its separation from the biomass. This is even more pronounced for the DES systems, since the final mixture always results in a viscous liquid. Therefore, efficient fractionation methods need to be developed to maximize the lignin yield.
Aqueous organic solvents such as ethanol and acetone are commonly employed for the separation of polysaccharide-rich residue from the final mixture. For the aqueous organic solvents, high solubility of DES and lignin is a key prerequisite, as this determines the yield of lignin recovered. We selected 90% aqueous ethanol and 80% acetone for the separation, as previous studies indicated that lignin had a relatively high solubility in these mixtures [1,2].

With this in mind, two parallel extractions using aqueous ethanol (**path 1** in Fig. S2a) and acetone (**path 2** in Fig. S2a) to separate polysaccharide residues were performed at 100°C for 6 h. After extraction, 34.3 g

of DES mixture was poured in 200 mL aqueous solvent (80% acetone and 90% ethanol, separately) and stirred for 2 h. The DES mixture had excellent miscibility in aqueous ethanol and acetone (**paths 1** and **2**). However, for path **2**, lignin precipitation occurred after removing the aqueous acetone. Moreover, stirring the polysaccharide-residue obtained from acetone diluted-DES mixture with another 200 mL fresh 90% ethanol for 2 hours, another 60 mg lignin extract was obtained apart from the 150 mg lignin obtained from the DES phase (Lignin from water-precipitation). The extract was submitted for 2D HSQC NMR analysis, as showed in the Fig. S45, the main signals were ascribed to polysaccharide and DES, and some weak signals from lignin were observed. This test indicated that the 80% acetone was unsuitable to separate the polysaccharide-rich residue from DES and lignin. Therefore, i 90% aqueous ethanol was employed for separating lignin from the DES and polysaccharide-rich residue.

Following the separation of the polysaccharide-rich residue, lignin was recovered from the ethanol-DES phase. Ethanol was first removed by vacuum evaporation to yield the lignin-DES solution (**path 1**, Fig. S2b). Isolating lignin from this phase presents several challenges. Our group previously developed a liquid-liquid extraction method using THF; however, in diol-based DES systems, this approach consistently led to significant ethylene glycol (EG) contamination, due to its leaching into the water phase. As shown in **path 3** (Fig. 1b), the resulting lignin formed a viscous oil requiring multiple brine washes, which introduced additional salt impurities necessitating further purification. Attempts to substitute THF with the less water-miscible 2-methyl THF (**path 4**, Fig. 1b) led to premature lignin precipitation that adhered to the separation funnel, reducing recovery and complicating handling.

Due to these limitations, we reverted to the classical water-precipitation method (**path 1**) for lignin recovery. While pH adjustment to 2 is typically employed to enhance precipitation, its effect in diol-based DES systems is rarely reported. Comparative experiments using Milli-Q water (E1, E3) and acidic water (E2, E4, pH = 2) under identical conditions showed negligible differences in lignin yield and structure (Tables S2 and S3).

Washing steps were found to critically influence yield. Consistent with prior findings, diol-modified lignin exhibited high water dispersibility. In one control experiment (E26), additional precipitation (1.6% lignin) occurred after the supernatant was left overnight post-shaking. This suggests that vigorous agitation during washing may lower yield by retaining lignin in the aqueous phase. GPC analysis (Fig. S2) confirmed that lignin recovered from secondary precipitation had lower molecular weight compared to that isolated through intensive washing.

These results indicate that milder washing, avoiding excessive agitation, improves lignin recovery from diol-based DES systems. Overall, water precipitation (**path 1**) proved to be the most efficient approach for separating lignin from the DES phase. Liquid–liquid extraction with THF or 2-methyl THF was less effective due to impurities and handling difficulties. Finally, any lignin fragments lost during precipitation can be recovered via THF extraction, ensuring high overall lignin recovery through this two-step approach.

S2.2. Lignin isolation from ChCl/EG/OA: fractionation under mild conditions

Reactive and labile aryl ether linkages play a critical role in determining the properties, distribution, and yield of monomers produced through hydrogenolysis and acidolysis. Therefore, in this study we attempted to optimize a milder fractionation process.

Initially, a 2h extraction was carried out at 80°C. During this initial extraction, birch particles were suspended in the transparent DES mixture. At the end of the extraction, the mixture exhibited a light pink color, and birch particles were recovered almost intact without visible physical change (see the state in Fig. S3b), partially indicating the lower efficacy of 80 °C temperature towards high-yield lignin isolation. This was confirmed by low lignin yield (1.2%, 8.6 mg, E5, detailed data is listed in Table S4), which was far less to submit a 2D HSQC NMR analysis. Increasing the retention time to 6 h and 12 h led to an increase of lignin yield of 2.5% (E5) for 6 h to 4.4% (E6) for 12 h. It seems that lignin yield was linearly correlated with the retention time under this temperature. A further increase of retention time to 24 h gave a yield of 7.8% (E8) which is slightly less than that of E6. However, this was accompanied by a significant increase of the mass loss from 8.5% to 23.8%, a negative effect for the selective isolation of lignin. 2D HSQC NMR was collected for the lignin obtained from E8, and 74% conservation of the total aryl ether linkage to that of corresponding enzyme milder acidolysis lignin (EMAL) was observed under this mild temperature. It seemed that the labile aryl ether linkage had a higher stability at mild conditions.

Molecular weight is a more sensitive index to reveal the structure alteration of lignin. Here, gel permeation chromatography (GPC) was used to measure the molecular weight and distribution of the lignin (Fig. S4). Average molecular weight (MW) slightly decreased from 9100 Da (E5) to 4190 Da (E9), indicating cleavage of linkages even at 80°C. Thus, in order to test the potential of β-O-4 content conservation and the maximum yield of lignin under this temperature, we further increased the retention time to 72 h, but only a slight increase of yield to 9.3% (E9), an even lower EG incorporation as well as a higher mass loss of 33.4% were observed. These data indicated depolymerization of other components such as labile hemicellulose and amorphous cellulose. However, there was still approximately 64% of β-O-4 content conserved, further indicating that the released lignin had a higher stability under relatively lower temperature.

Since unsatisfactory yields were obtained under mild temperature, further increase of the temperature to 100°C and retention times to 6 h and 12 h allowed lignin yields of 11.02±0.36% (E10) and 14.01±0.88% (E16), respectively. However, such a short retention time had close and even higher mass loss than that of E8, and almost the same retention of β-O-4 as that of E9 was obtained. It seemed that increased temperature had a greater contribution than the prolongation of retention time towards high mass loss and low β-O-4 content. From these preliminary results about lignin isolations via DES, it was indicated that the retention time and particularly temperature had a dramatic effect on the mass loss of starting material, yield and structural integrity of lignin, which deserves a further comprehensive study.

S2.3. Lignin isolation from ChCl/EG/OA: the influence of water addition and particle size

The natural amphiphilicity of lignin suggests that it acts not only as a H-bond acceptor but also as a donor. Moreover, a previous study on lignin isolation from DES reported that the efficiency of the extraction might be related to the Kamlet-Taft polarity parameters.[3] H-bond donating and accepting ability as well as interactions caused by polarity are the main contributions to this value. This might explain why the efficacy of lignin isolation varied upon the constitutions of DES, as observed from the above studies. Due to the strong ability of hydrogen-accepting, ChCl has high hygroscopicity when in contact with open air.[4,5] Therefore, water contamination is unavoidable, and impacts interactions between H-bond acceptance and donation, thereby affecting the solvents physical properties and performance. The influence of water addition on the lignin extraction of DES was studied (Table S14-S15 and Fig. S6a). Isolation with 10% water addition was performed at two conditions: 80°C for 24 h (E32), and 100°C for 6 h (E35). A slight decrease of aryl ether linkage and an increase of condensation were found for the extraction under lower temperature and long retention time, while no big difference was observed for the lignin obtained from high temperature, except of the decrease of EG incorporation. However, the MW decreased for both

extractions compared with the extraction without water addition (Fig. S5d). The extraction with water addition indicated water addition did not influence the yield of lignin but induced more linkage cleavage of lignin and dropping of MW. MW of the lignin might be tuned by the addition of water under proper conservation of aryl ether linkage.

The size of the biomass particles also affects lignin extraction, particularly for the extractions under milder conditions. Ball milling is an effective physical treatment that not only decreases the size of the biomass but also breaks the cell wall structure, significantly enhancing the efficacy of lignin extraction.[6] Birch (less than 10 μm, almost the minimum size achieved from ball milling) milled by following a published protocol was extracted at 80 °C for 24 h (E31). The yield of lignin almost doubled from the extraction of E8. Less lignin but a relatively better conservation of linkage was observed when the size of birch increased from a range of 355 μm-500 μm (E33) to a size higher than 500 μm (E34). From these extractions, it was found that the particle size can significantly influence the DES to isolate lignin.

S.2.4. Analysis of the organic extracts from water precipitation to detect lignin oligomers and monomeric fragments

As outlined in Scheme 1, our lignin recovery procedure consisted of the dropwise addition of the concentrated lignin-liqueur into water upon which the precipitated lignin was recovered by filtration (Step 3 in Scheme 1) in high purity. We have also attempted to qualitatively understand the residual, water-soluble products remaining in the aqueous phase. These could consist of EG-functionalized lignin oligomers as well as monophenolic fragments. With this in mind, the combined aqueous phase from the fractionation experiments at 80°C/24 h, 100°C/2h, 120°C/2 h, 120°C/4h, 120°C/6h, 120°C/12 h, 160°C/2 h, 180°C/2h and 200°C/1 h were collected and extracted with THF (See detailed steps in Figure S6) and further evaluated by 2D HSQC NMR, GPC, and GC-MS analysis. Structural quantification of the extracts that yielded sufficiently strong lignin-derived signals for detection by 2D HSQC NMR is provided in Table S16

After solvent removal, the molecular weight and distribution of the THF extract were determined and profiled in Figure S9. Monomers and small oligomers were observed in all the samples, albeit in different nature and quantity. Among them, extracts obtained from milder fractionation conditions, such as at 80°C and 100°C, showed a high MW (> 1000 Da, Figure S9), while 2D HSQC NMR spectra (Figure S1 below or Figure 6 in the main text) indicated almost no typical signals for lignin in the aromatic region, with the signals ascribed to products from carbohydrates, most likely hemicellulose. These results are consistent with the low lignin yield (7.2%) isolated from this condition, despite the high mass loss (23.8%). However, more typical signals for lignin and aromatic monomers were clearly observed for samples obtained from conditions above 120°C, indicating lignin fragments which were not originally precipitated, and remained in the aqueous phase. The intensity of these signals increased with prolonged reaction times (from 2 hours to 12 hours at 120°C) and temperature (160 and 200°C). Moreover, it was shown that the lignin fragments obtained from harsh conditions are more condensed, as indicated by NMR measurements. For example, comparing the spectra of oils extracted from the fractionation experiments at 120°C, 2 hours and 12 hours, respectively, the condensation increased from 10.1/C9 unit to 26.5/C9 unit (Table S16).

In addition, EG incorporation of the lignin fragments was also observed from the 2D HSQC NMR spectra of samples from fractionations at temperatures higher than 120°C (Figure S1 below or Figure 6 in the main text).

The composition analysis of the monomeric range of the THF extracts recovered after DES treatments at 80°C/24h and 200°C/1h was performed by GC-MS (Figure S1 below), the original mass spectra were profiled in Figure S48 in the SI), and the product distributions displayed marked differences. Most

compounds identified for the extract obtained at 80°C were aliphatic products or carbohydrate-derived compounds, possibly also originating by EG fragments, in line with the observations from 2D HSQC NMR. In contrast, the main products found for extract obtained at 200°C and 1 hour contained aromatic monomers such as phenolic alcohols **1**, **4** and **6**, aldehyde **8** and **10**, as well as ketone products **2**, **5** and **7**, which are typical products from lignin acidolysis (Scheme S1) and have also been reported in previous lignin depolymerization studies under acidic conditions [7-12]. Aromatic acetal such as products **3** (confirmed by authentic standard, Figure S11) and **9** were also clearly observed. These are formed by the reaction between the aromatic C2 aldehyde with EG through diol-assisted lignin depolymerization/fractionation. [13,14] Similar products were observed for extract obtained from 120°C and reaction times of 2 h and 12 h (Figure S11), suggesting some lignin depolymerization took place already at 120°C, consistent with literature. Summing up, at 80°C/24h, mass loss mainly results from carbohydrate-derived oligomers and monomers, though their precise structures remain challenging to confirm. This however could be an excellent method for the mild and selective removal of hemicellulose and derived fragments under very mild conditions. At temperatures above 120 °C, an increased fractionation efficiency boosts the removal of lignin from the lignocellulose matrix. Therefore, it is reasonable that residual lignin-derived products, such as EG-functionalized lignin oligomers (see GPC analyses Figure S9 and 2D HSQC NMR spectra in Figure S1 or Figure 6 in the main text) and lignin-derived monomers, and some residual condensed lignin (mainly at 200°C), are recovered from the THF extracts. These, together with the carbohydrate-derived products, are likely to contribute to the overall mass balance detected at such conditions (21.9% at 80°C/24h to 44.6% at 200°C/1h).

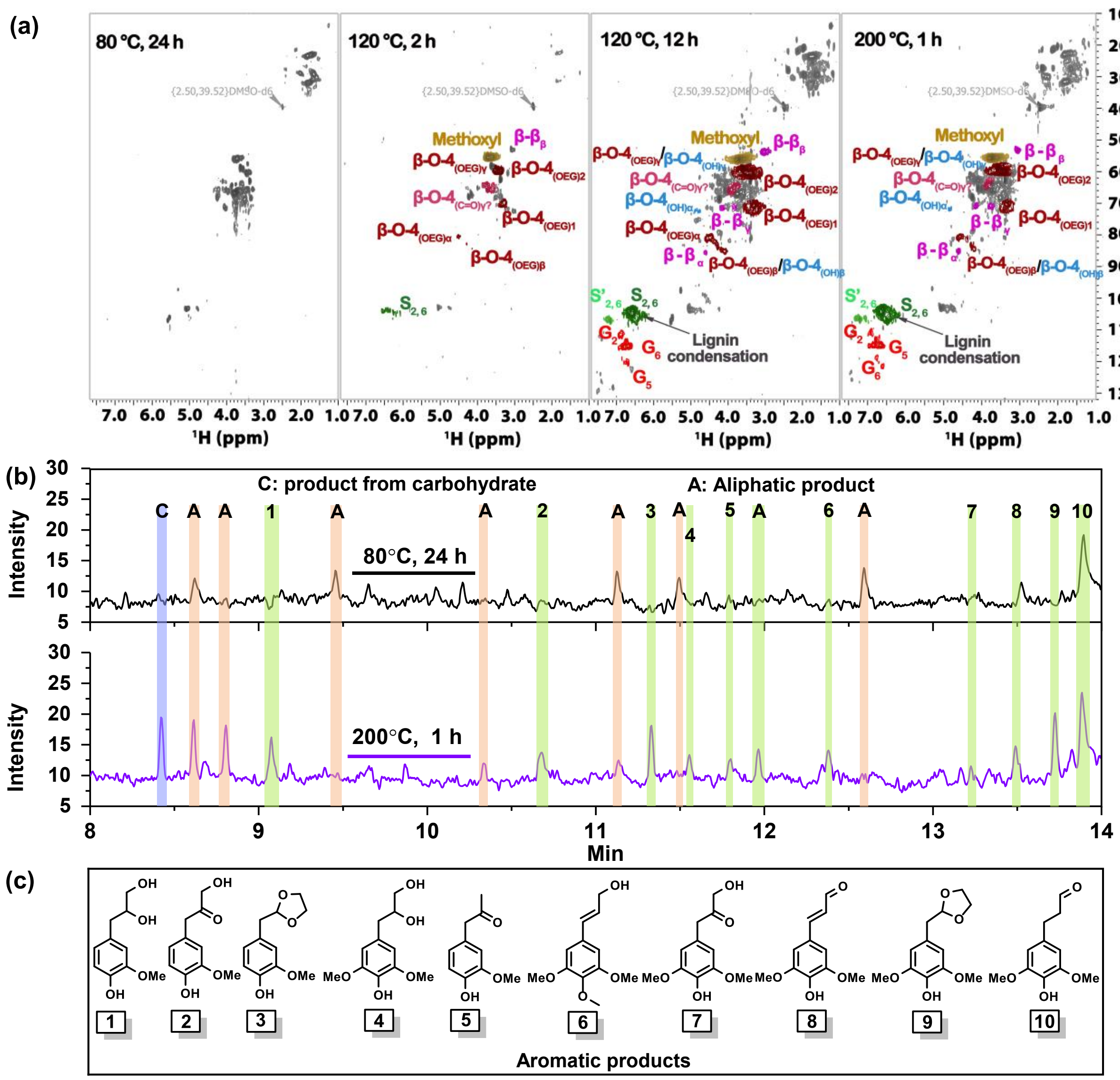

Figure S1. Composition and structural analysis of the lignin fragments obtained from the THF extraction of the liquid phase collected from DES treatment of birch with ChCl/EG/OA; (a) 2D HSQC NMR spectra (300 Hz, DMSO-d_6) of the oil. Here it can be seen that at mild conditions (80°C/24 hours), products primarily derived from carbohydrates (green spots) are detected. As the temperature increases beyond 120°C, lignin signals (β-O-4(OH), β-O-4(OEG), β-β) become prominent, with their intensity rising as fractionation time extends from 2 to 12 hours. More severe conditions, such as 160°C and 200°C, lead to increased lignin condensation (S, G units); reaction conditions: 16.8 g of ChCl, 14.4 g of EG, 3,12 g of OA (ChCl/EG 1:2 molar ratio, OA 10 wt% calculated on the total mass of ChCl and OA) 4 g of birch, 80°C/24 h, 120°C/2 h and 12 h 200°C/1 h. β-O-4(OH), aryl-alkyl ether, β-O-4(OEG), aryl-alkyl ether with EG incorporation at the α position, β−β, resinols ; (b) The GC-MS chromatography of the products. See original MS spectra in Figures S9-S10 in the SI (c) Peak assignment of the products. The peaks were assigned based on the fragmentation pattern and previous literature knowledge[15,30]. Authentic standard was used for the acetal **3**.

S3. Supplementary figures

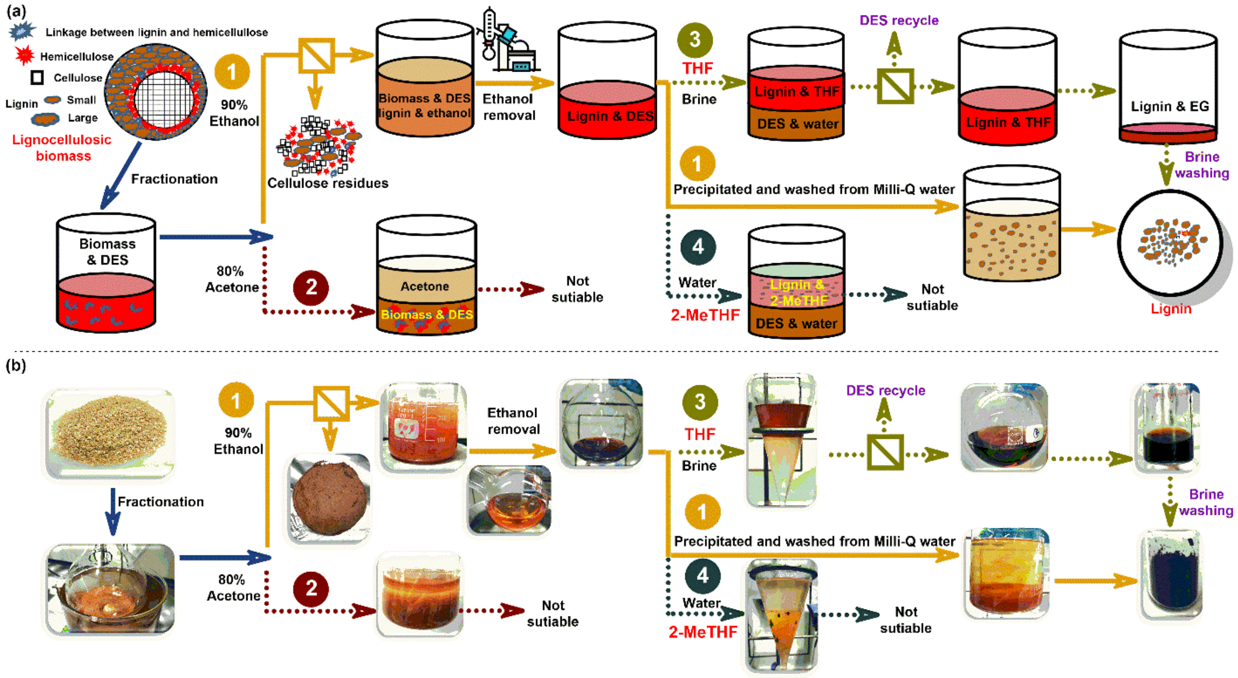

Fig. S2. Lignin isolation protocol. **a** Schematic illustration of lignin fractionation and recovery from the diol-based DES. Water precipitation is still the most effect way to separate lignin from the DES phase after increasing the loading of biomass before the DES fractionation. **b** Exemplified actual lignin fractionation by following the protocols illustrated in scheme **a**.

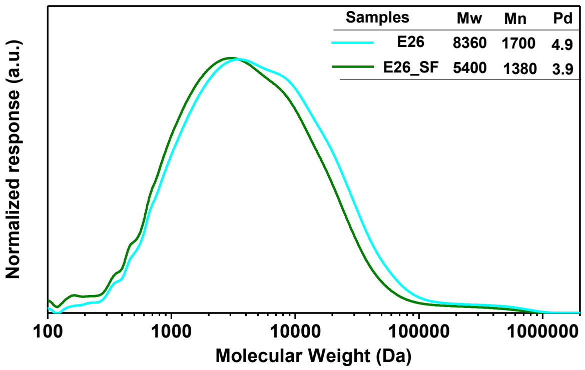

Fig. S3. The molecular weight and distributions of lignin obtained from water precipitation (E26) and reprecipitation (E26_SF) of the supernatant phase obtained from washing steps.

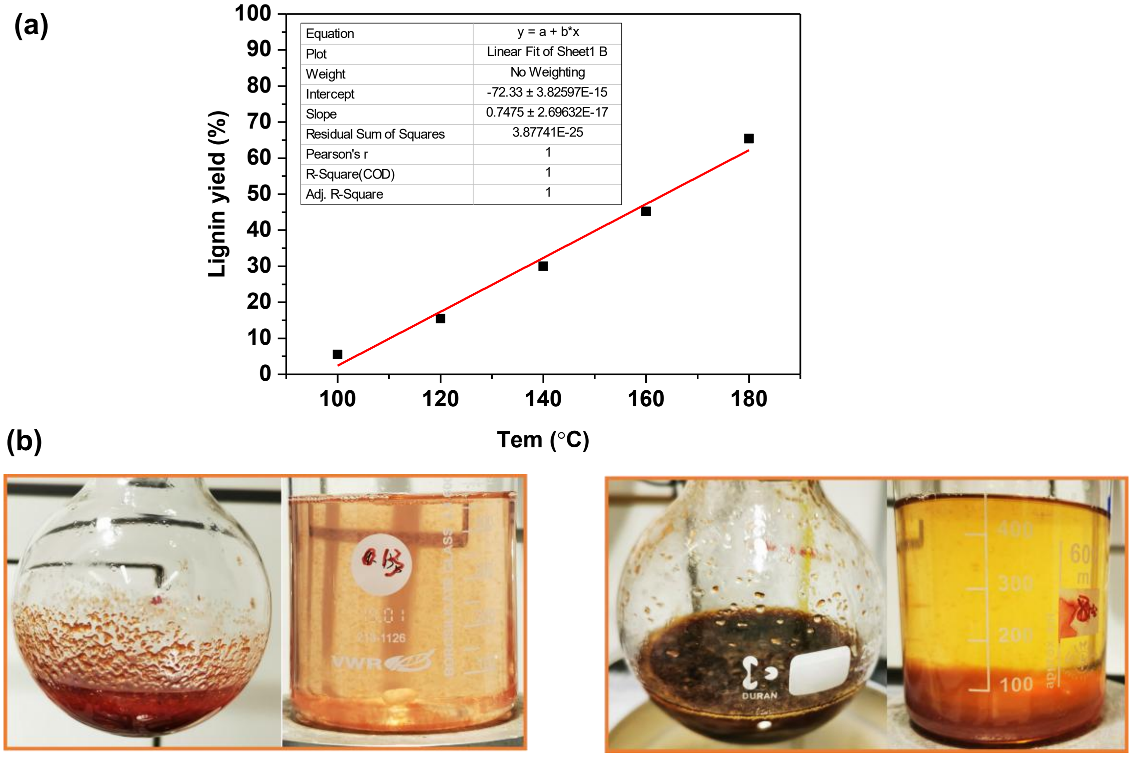

Fig. S4. The correlation between lignin yield and temperature and the pictures of the DES after fractionating the birch under 80 and 120 C for 12 and 2 respectively. a, the linear fitting for the lignin yield and temperature of the DES lignin extraction. b, Pictures of the DES mixture at the ending of the treatment and precipitation of lignin from water for the treatment at 80°C and 120°C For 2 h. (c), fractionation under 80°C and 12 h; (d), fractionation under 120°C and 2 h. Treatment condition: see E1 and E10 in Table S2 and S4

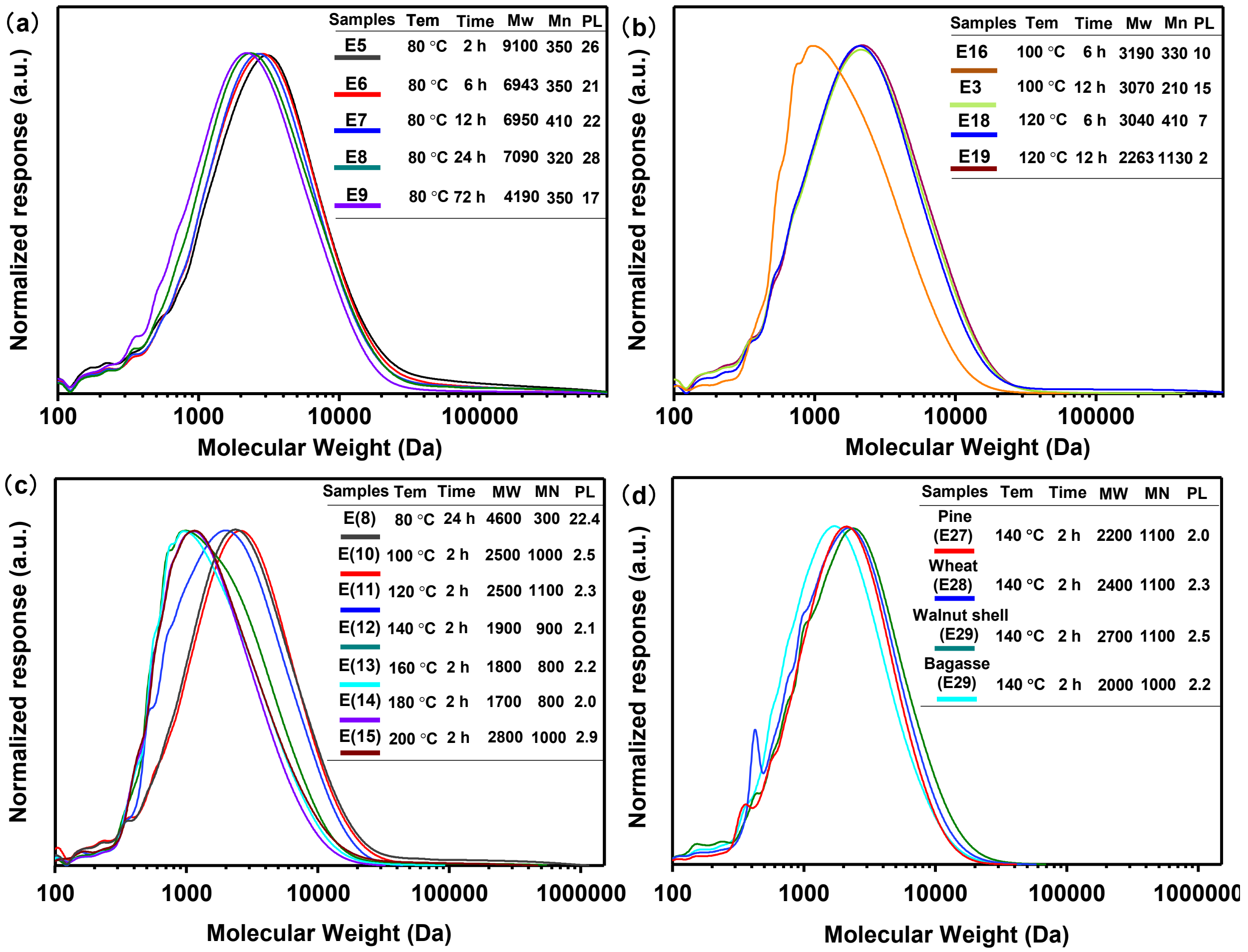

Fig. S5. The molecular weight and distributions of lignin obtained from ChCl/EG/OA (16.8 g of ChCl, 14.4 g of EG, 3,12 g of OA) DES extraction at various temperatures and times, using 4g of birch. a) and b) were not corrected by the incorporation of ethylene glycol and acetylation, while c) was corrected by the incorporation of ethylene glycol and acetylation. a) Molecular weight variations resulting from the fractionation process at mild conditions (80°C) and different time ranges. It is possible to note that MW decreases upon longer reaction times, indicating cleavage of linkages even at such conditions; b) Molecular weight variations resulting from the fractionation process at higher temperatures (> 80°C) and different temperatures and time ranges. The same trend indicates that harsher conditions promote cleavage of linkages, hence lower MWs. c) Molecular weight variations resulting from the fractionation process at different temperatures and time ranges. An initial decrease from 4600 Da to 1700 Da for the fractionation under 80°C (E8) and 180°C (E14) was observed, resulting from more extensive cleavage of linkages at higher temperatures. At 200 °C the MW went up to 2800 Da, revealing recondensation may occur at harsher conditions. d) Molecular weight variations resulting from the fractionation process with different biomass.

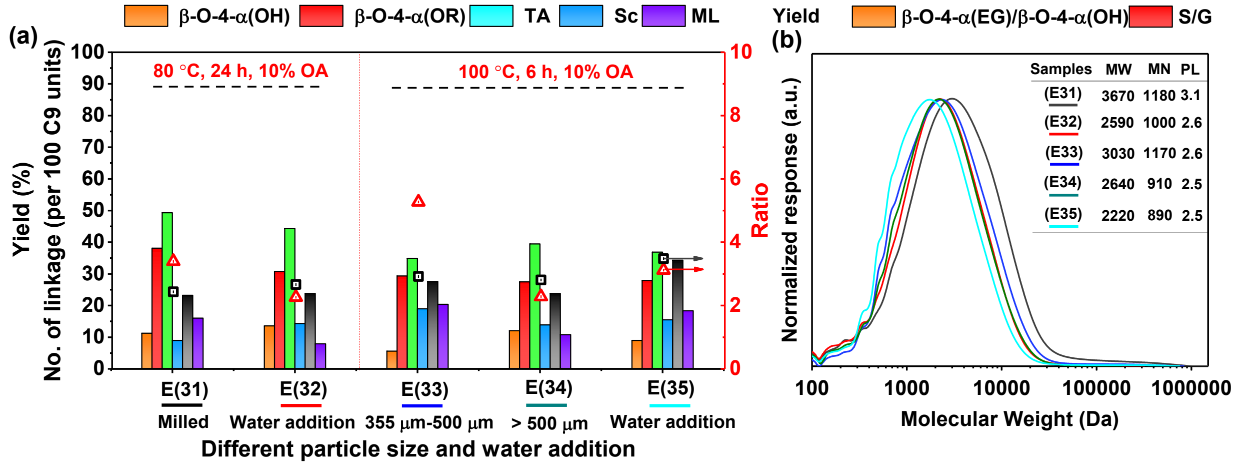

Fig. S6 Influence of particle size and water addition treating birch with ChCl/EG/OA at different times and temperatures (See detailed data and conditions in SI, Table S12-S13); β-O-4(OH), aryl-alkyl ether, β-O-4(OEG), aryl-alkyl ether with EG incorporation at the α position, β-5, phenylcoumaran, β–β, resinols, TA, total aryl ether linkage [β-O-4(OH) + β-O-4(OEG)], TL, total linkage (TA + β-5 + β–β); Sc, the condensation calculated by 100 C9; ML, main mass loss of starting material. a) reaction conditions: 16.8 g of ChCl, 14.4 g of EG, 3,12 g of OA (ChCl/EG 1:2 molar ratio, OA 10 wt% calculated on the total mass of ChCl and OA), 4 g of birch. The yield and detailed structural information calculated from 2D HSQC NMR (detailed reaction condition and linkage data are listed in Table S12 and S13, and original spectra in Fig. S38-S43); (b) The molecular weight and distribution of the corresponding lignins obtained.

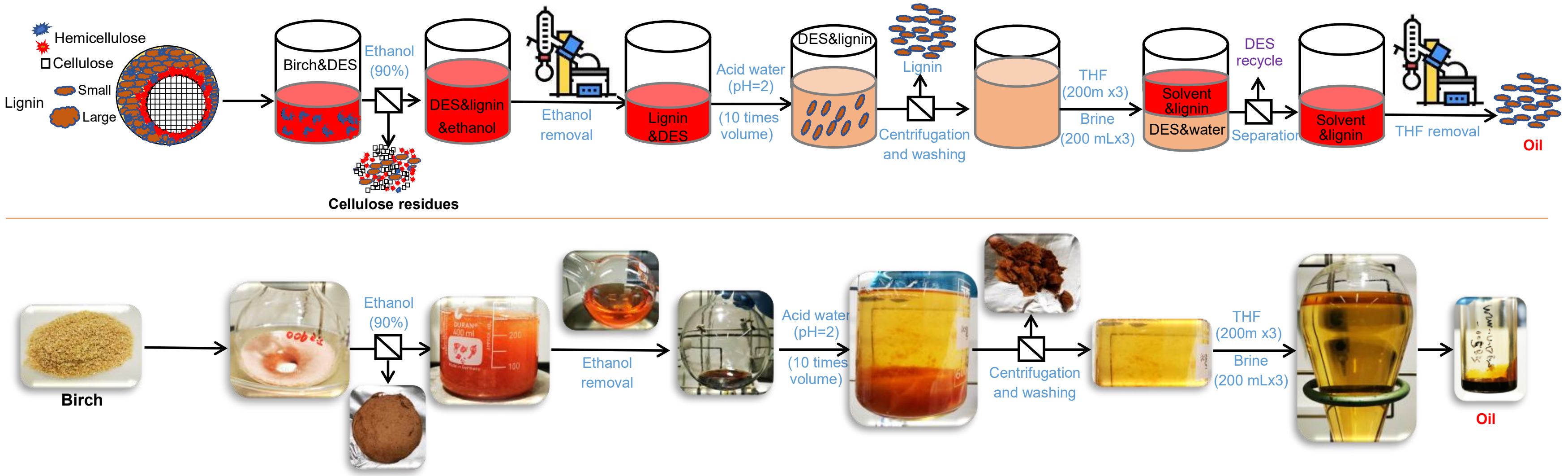

Fig. S7. Illustration of the steps used for obtaining the oil from the liquid phased collected from water precipitation and washing steps.

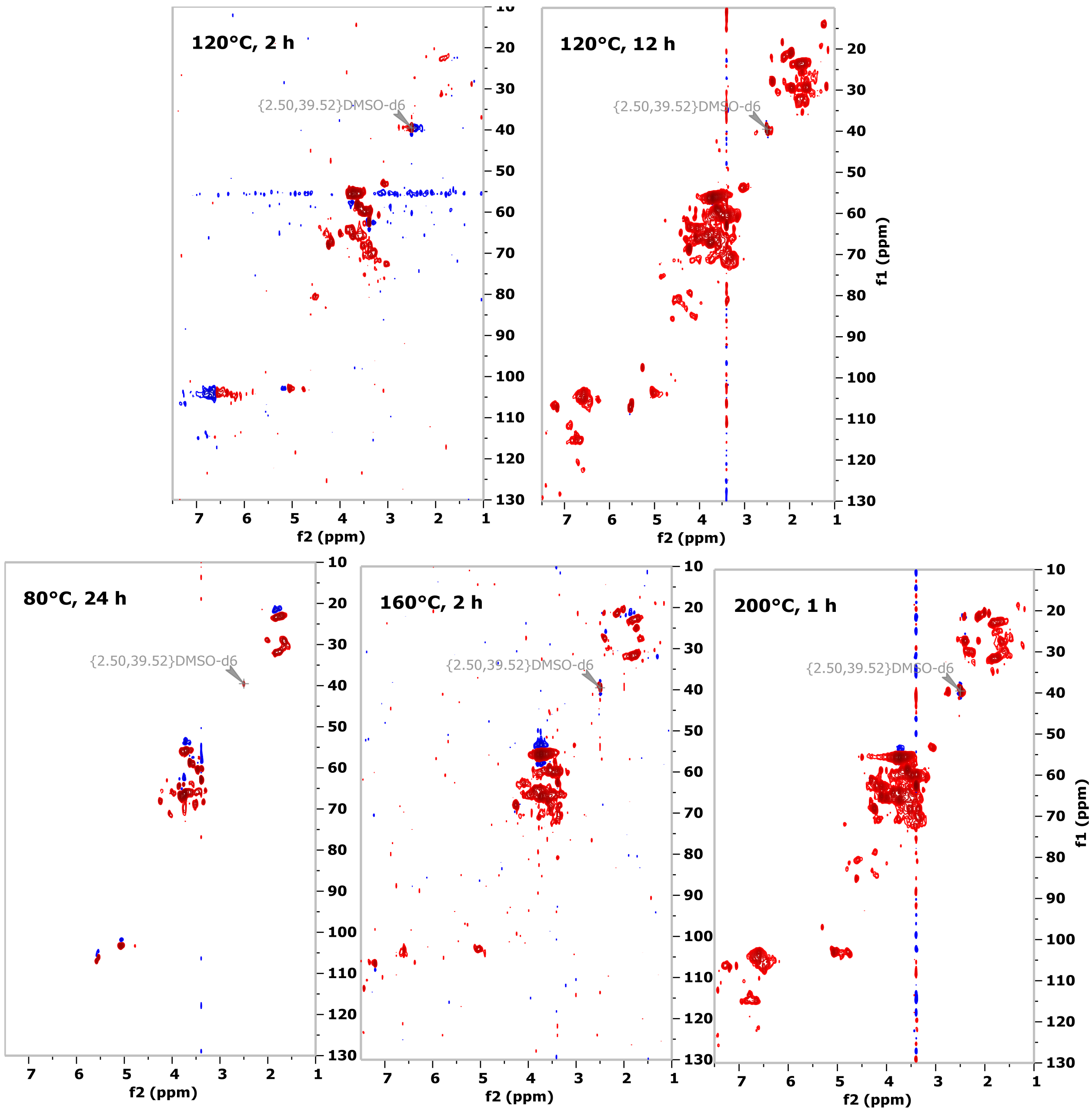

Fig. S8. 2D HSQC NMR of the oil obtained from THF extraction of the supernatant collected from the water precipitation and washing steps (Step 3).

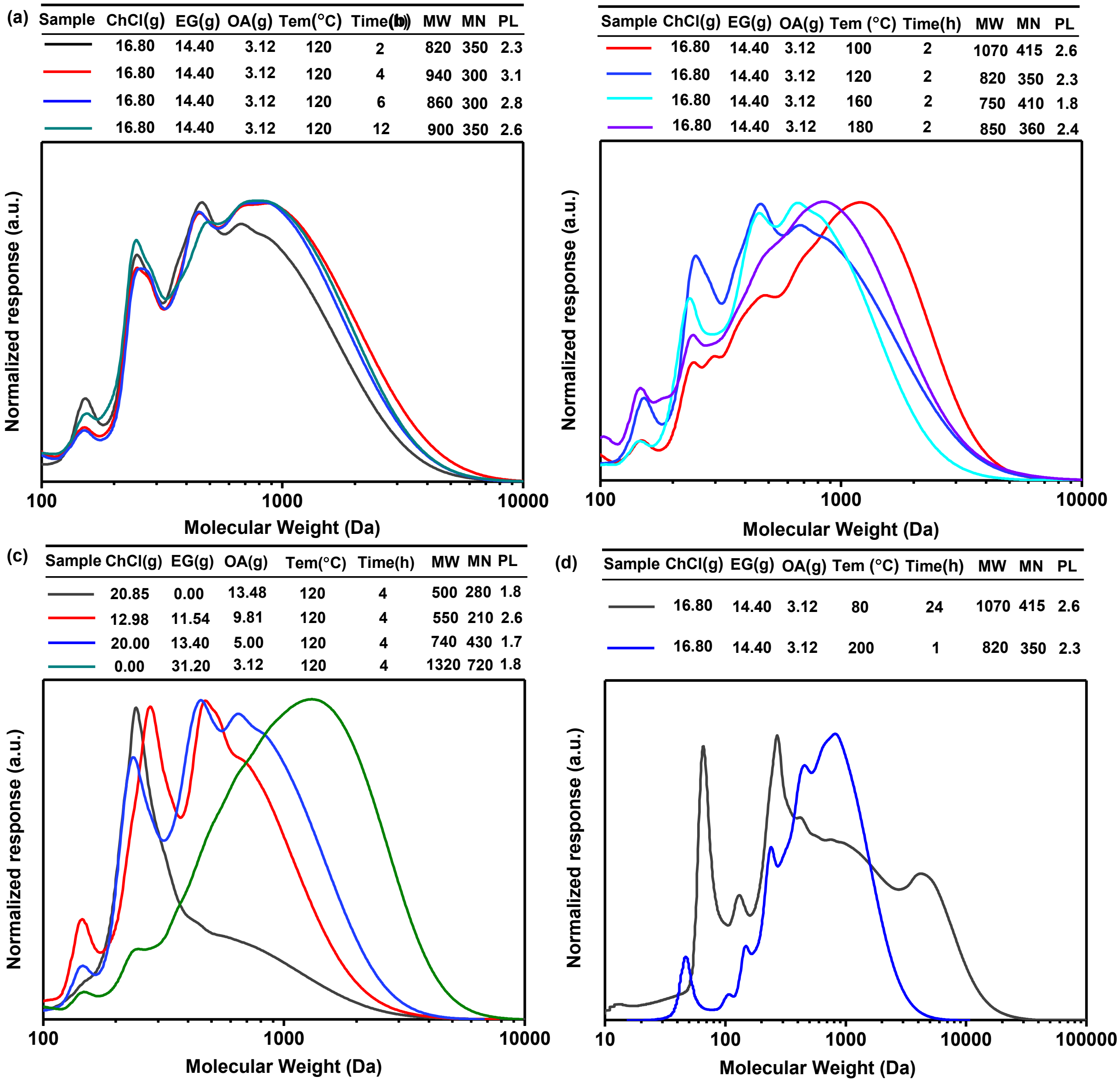

Fig. S9. Molecular weight and distribution of oils extracted from the liquid phases obtained from lignin precipitation and washing steps. a) oils obtained from the DES fractionation under 120°C for different times; b) oils obtained from the DES fractionations under different temperatures for 2 h; c) oils obtained from fractionations under DES or solvents prepared by different proportions of ChCl, EG, and OA; d) oils obtained from DES fractions under 80°C and 200°C for 24 h and 1 h, respectively.

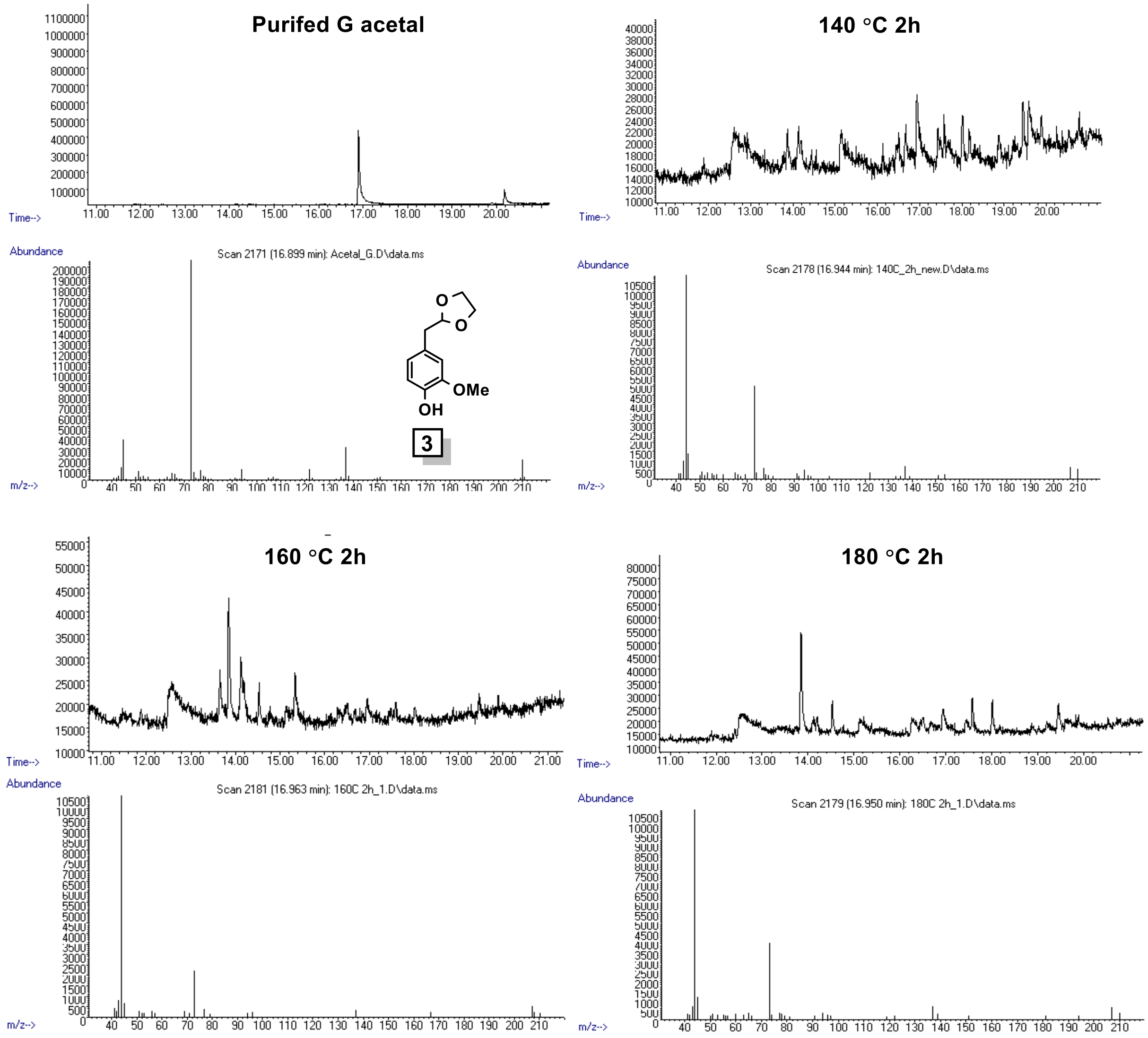

Fig. S10. GC–MS data confirming the formation of the G acetal from the oil fractions recovered after THF extraction (Step 4) of the liquid phase collected from DES treatment of birch (4 g) with ChCl/EG/OA (16.8 g of ChCl, 14.4 g of EG, 3,12 g of OA) for 2 h at 140°C, 160°C, 180°C, respectively.

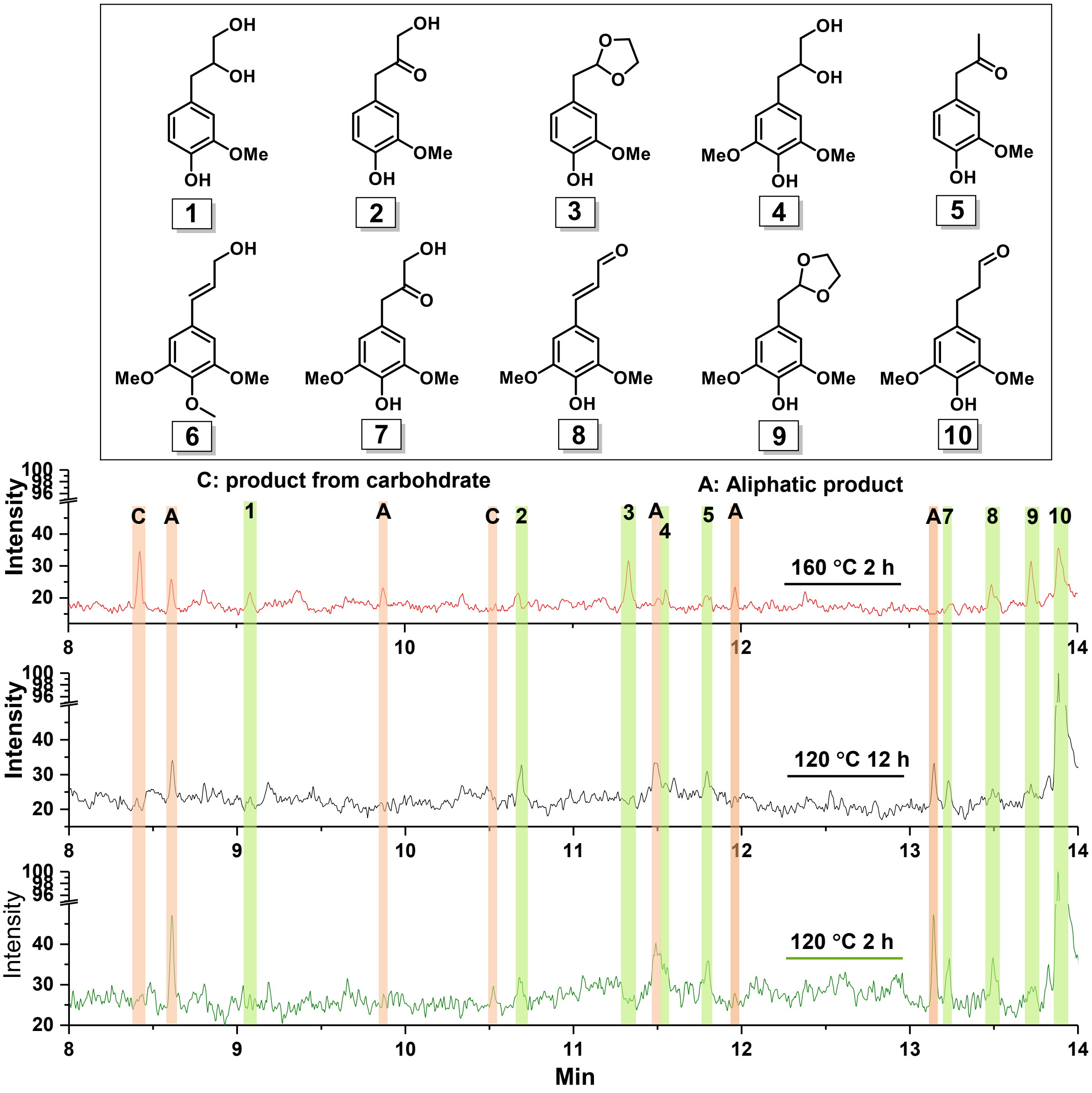

Fig. S11. The GC-MS spectra of the oil fractions recovered after THF extraction (Step 4) of the liquid phase collected from DES treatment of birch (4 g) with ChCl/EG/OA (16.8 g of ChCl, 14.4 g of EG, 3,12 g of OA) 160°C, 2 h, 120°C, 12 h and 120°C, 2h.

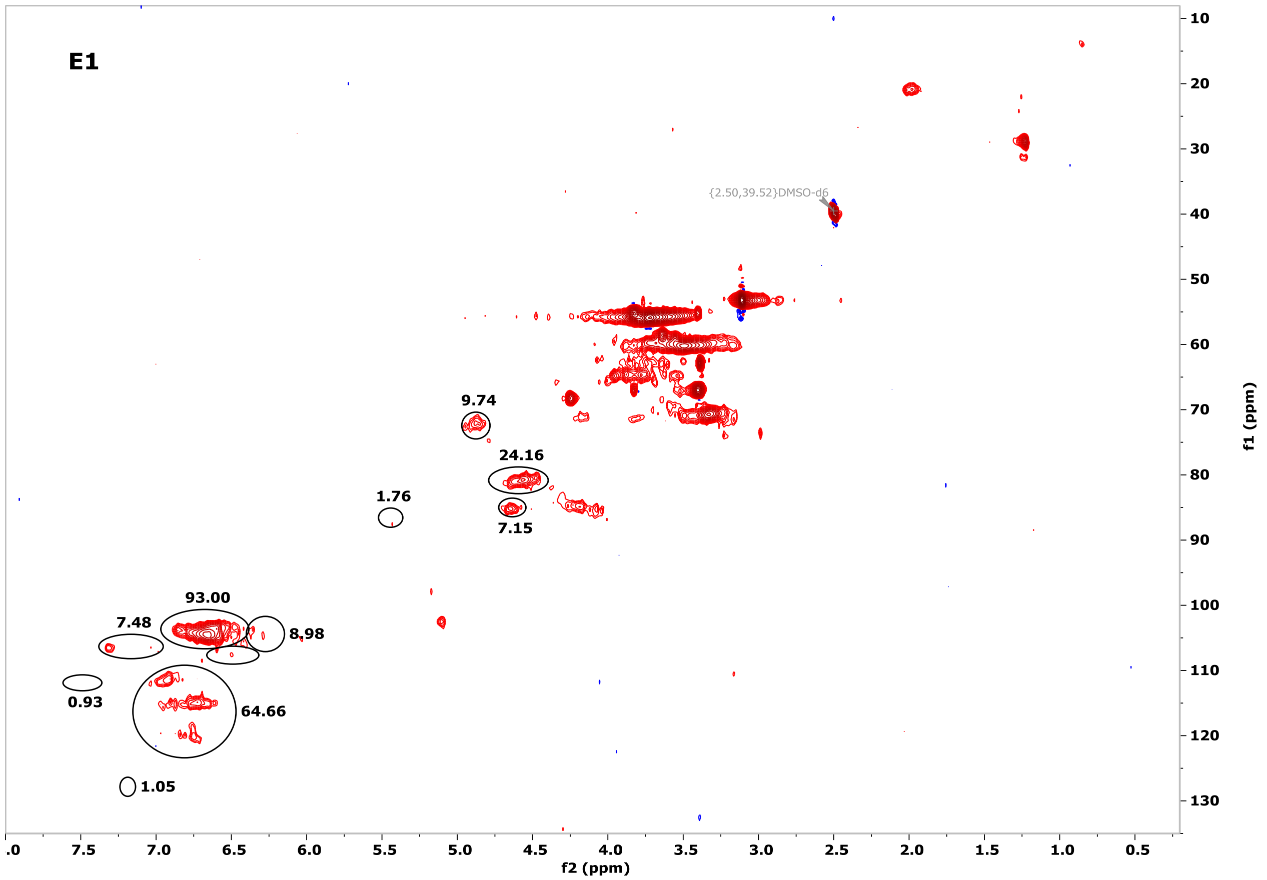

Fig. S12. 2D HSQC NMR (300 Hz, DMSO-d_6) and signal integration of lignin obtained from treating birch in the ChCh/EG/OA at 120°C for 2 h. Reaction condition: 4 g birch, 16.8 g ChCl, 3.6 g ethylene glycol, 3.12 g oxalic acid, 120°C, 2 h (See detailed reaction condition and calculating data in Table S2, E1 and Table S3, E1). Lignin was recovered by acid water (pH=2) precipitation.

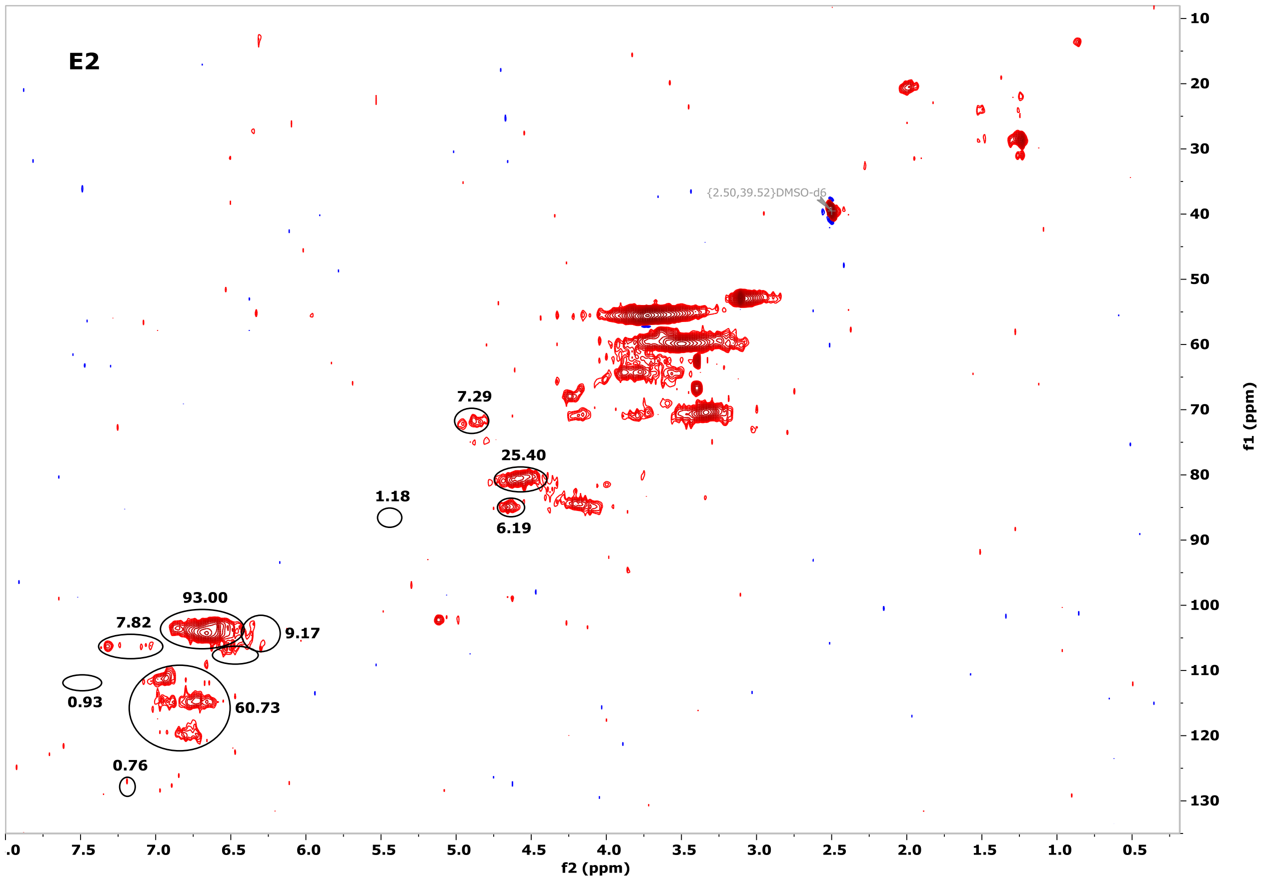

Fig. S13. 2D HSQC NMR (300 Hz, DMSO-d_6) and signal integration of lignin obtained from treating birch in the ChCh/EG/OA at 120°C for 2 h. Reaction condition: 4 g birch, 16.8 g ChCl, 3.6 g ethylene glycol, 3.12 g oxalic acid, 120°C, 2 h (See detailed reaction condition and calculating data in Table S2, E2 and Table S3, E2). Lignin was recovered by Milli-Q water precipitation.

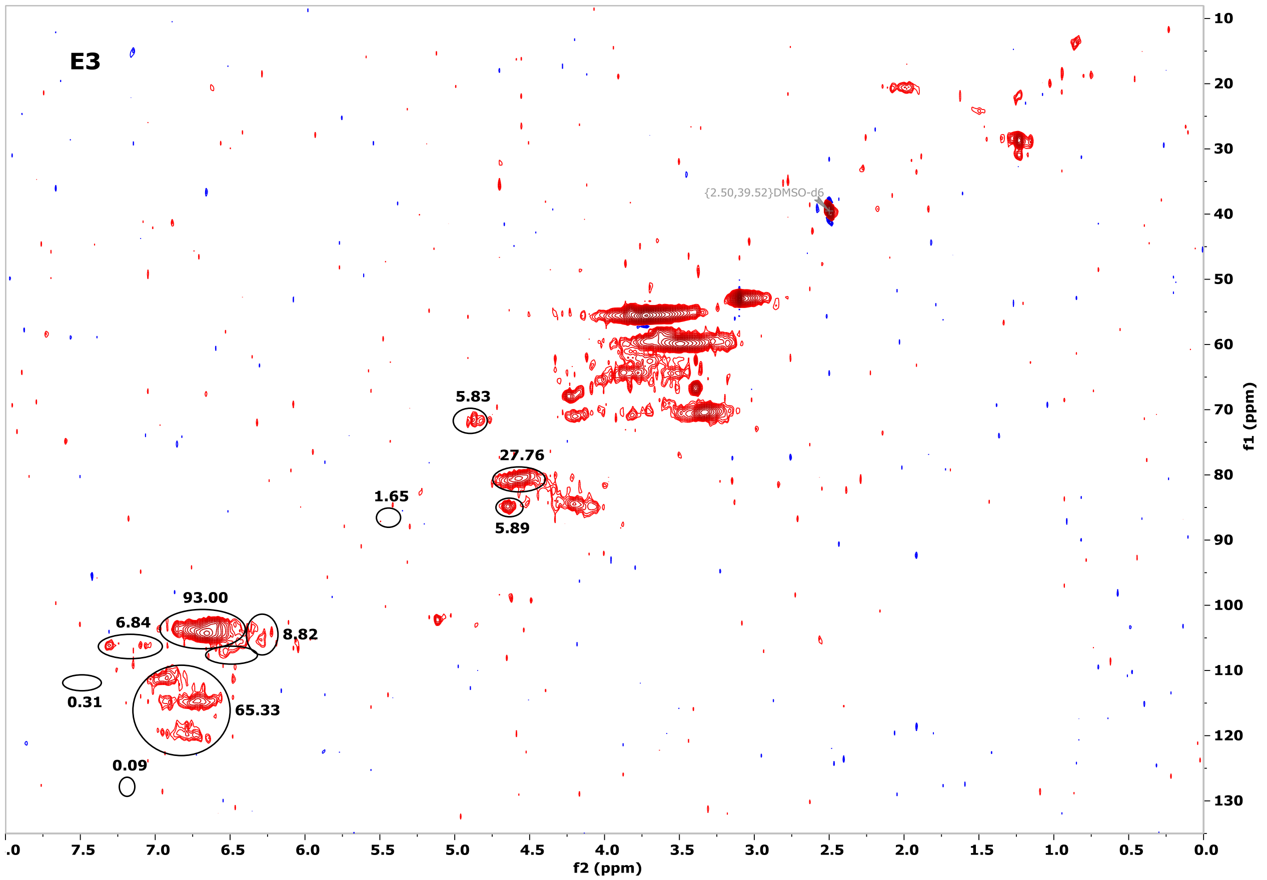

Fig. S14. 2D HSQC NMR (300 Hz, DMSO-d_6) and signal integration of lignin obtained from treating birch in the ChCh/EG/OA at 100°C for 12 h. Reaction condition: 4 g birch, 16.8 g ChCl, 3.6 g ethylene glycol, 3.12 g oxalic acid, 100°C, 12 h (See detailed reaction condition and calculating data in Table S2, E3 and Table S3, E3). Lignin was recovered by acid water (pH=2) precipitation.

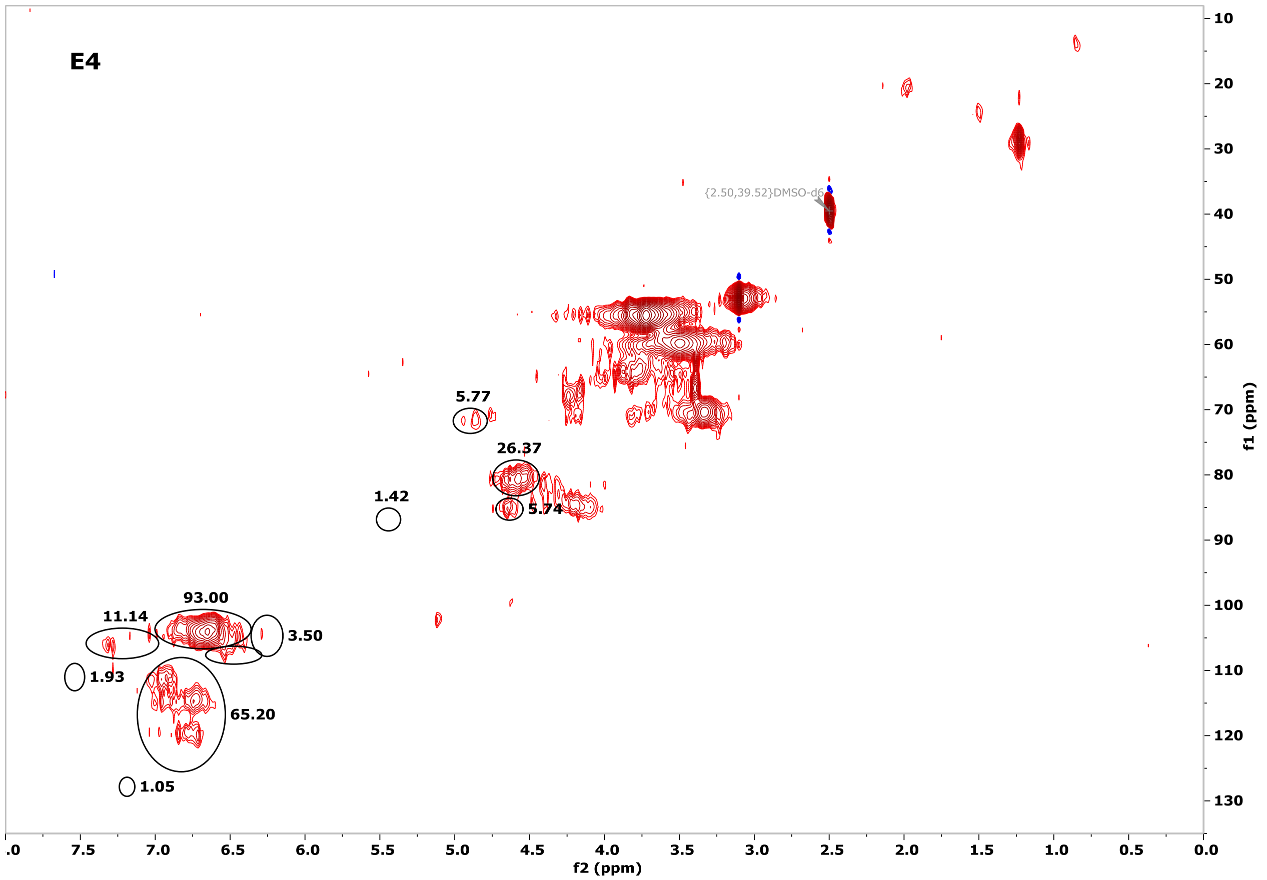

Fig. S15. 2D HSQC NMR (300 Hz, DMSO-d_6) and signal integration of lignin obtained from treating birch in the ChCh/EG/OA at 100°C for 12 h. Reaction condition: 4 g birch, 16.8 g ChCl, 3.6 g ethylene glycol, 3.12 g oxalic acid, 100°C, 12 h (See detailed reaction condition and calculating data in Table S2, E4 and Table S3, E4). Lignin was recovered by Milli-Q water precipitation.

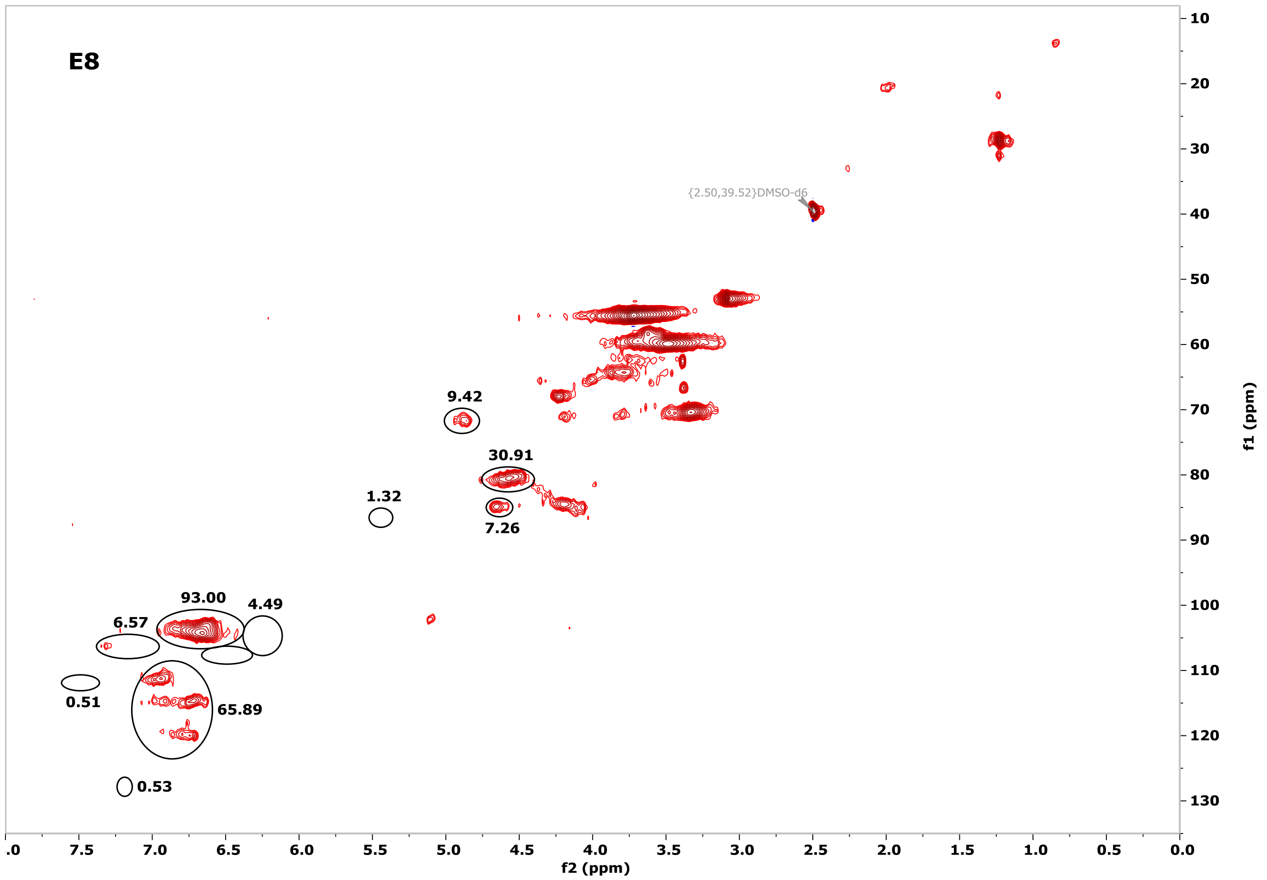

Fig. S16. 2D HSQC NMR (300 Hz, DMSO-d_6) and signal integration of lignin obtained from treating birch in the ChCh/EG/OA at 80°C for 24 h. Reaction condition: 4 g birch, 16.8 g ChCl, 3.6 g ethylene glycol, 3.12 g oxalic acid, 80°C, 24 h (See detailed reaction condition and calculating data in Table S4, E8 and Table S5, E8).

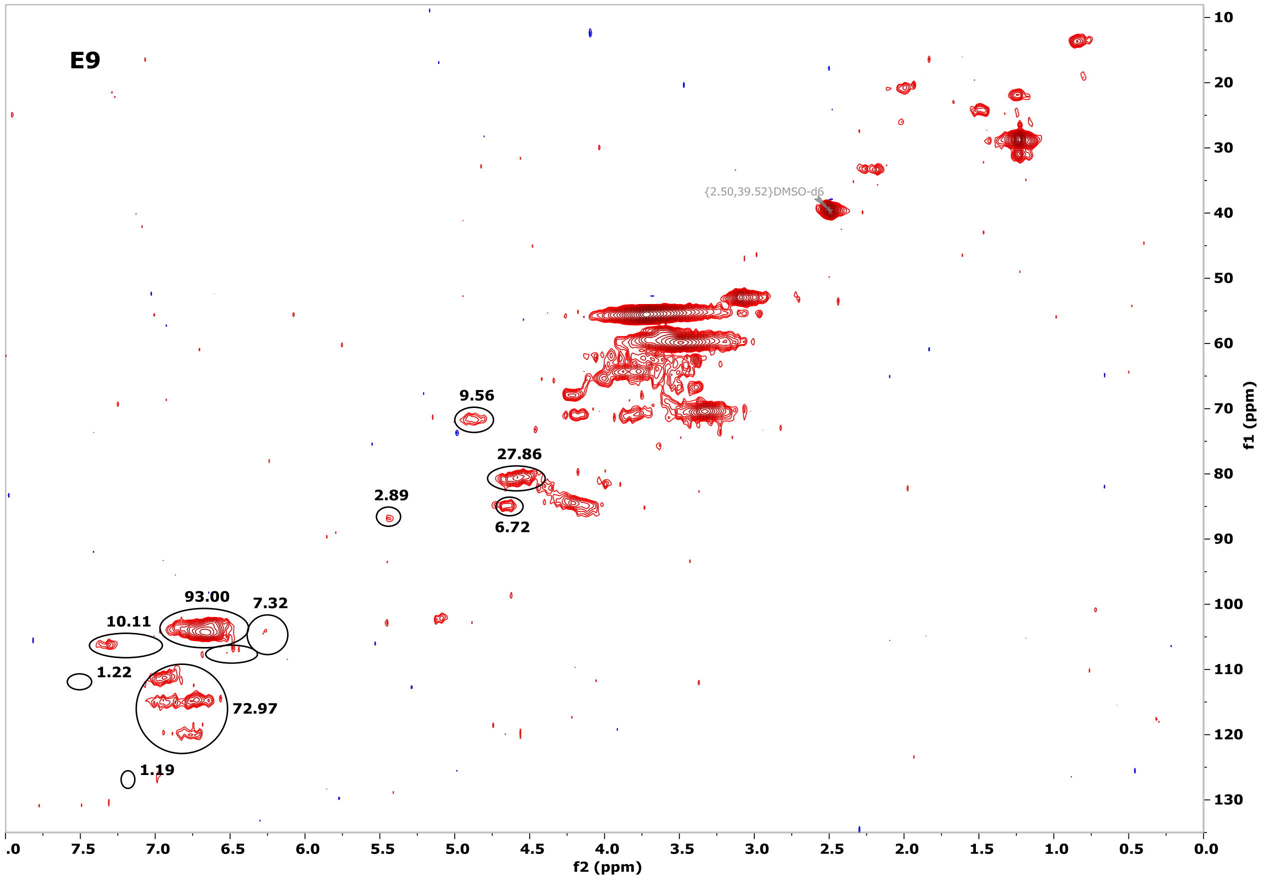

Fig. S17. 2D HSQC NMR (300 Hz, DMSO-d_6) and signal integration of lignin obtained from treating birch in the ChCh/EG/OA at 80°C for 72 h. Reaction condition: 4 g birch, 16.8 g ChCl, 14.4 g ethylene glycol, 3.12 g oxalic acid, 80°C, 72 h (See detailed reaction condition and calculating data in Table S4, E9 and Table S5, E9).

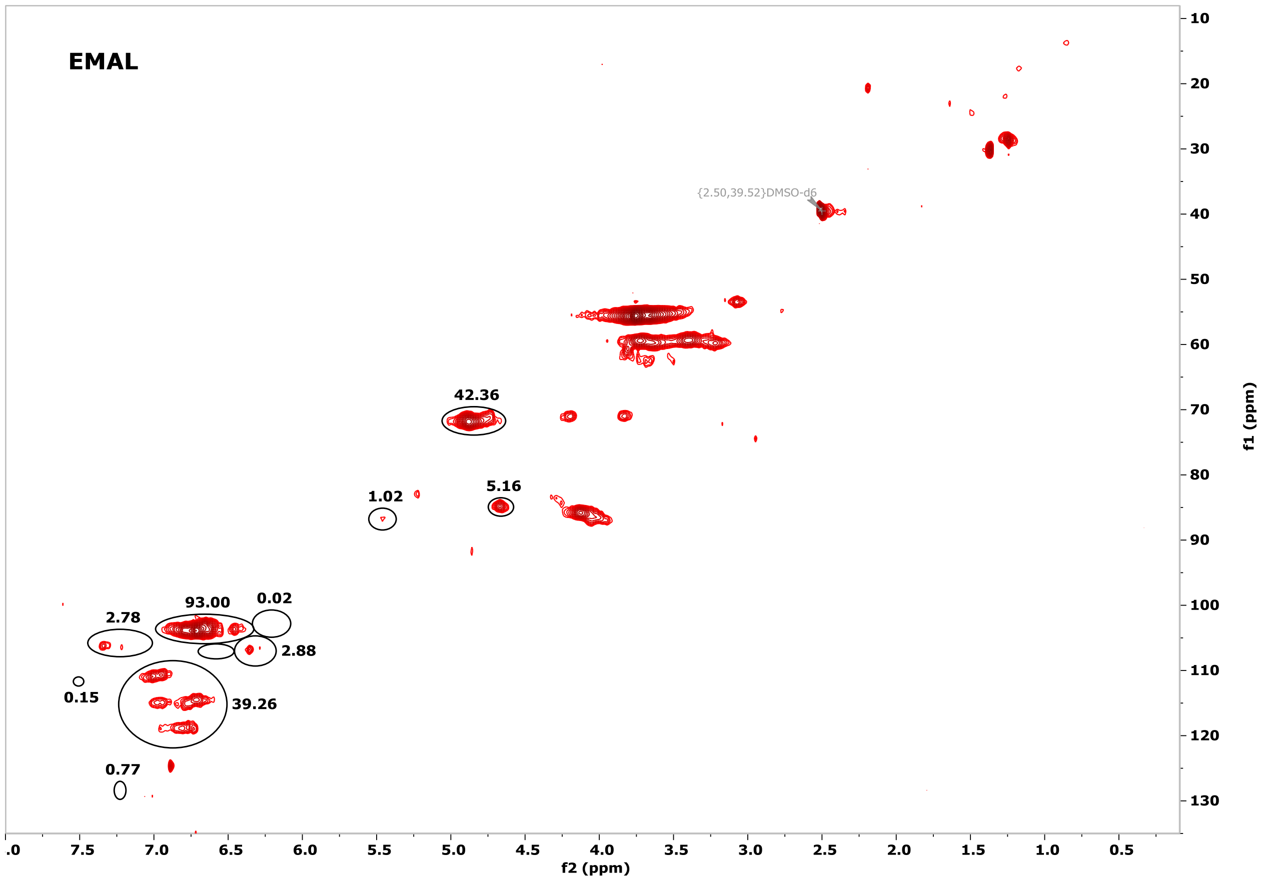

Fig. S18. 2D HSQC NMR (300 Hz, DMSO-d_6) and signal integration of enzyme mild acidolysis lignin. (See detailed reaction condition in **Supplementary experimental part S1.3** and calculating data in Table S9, MWL).

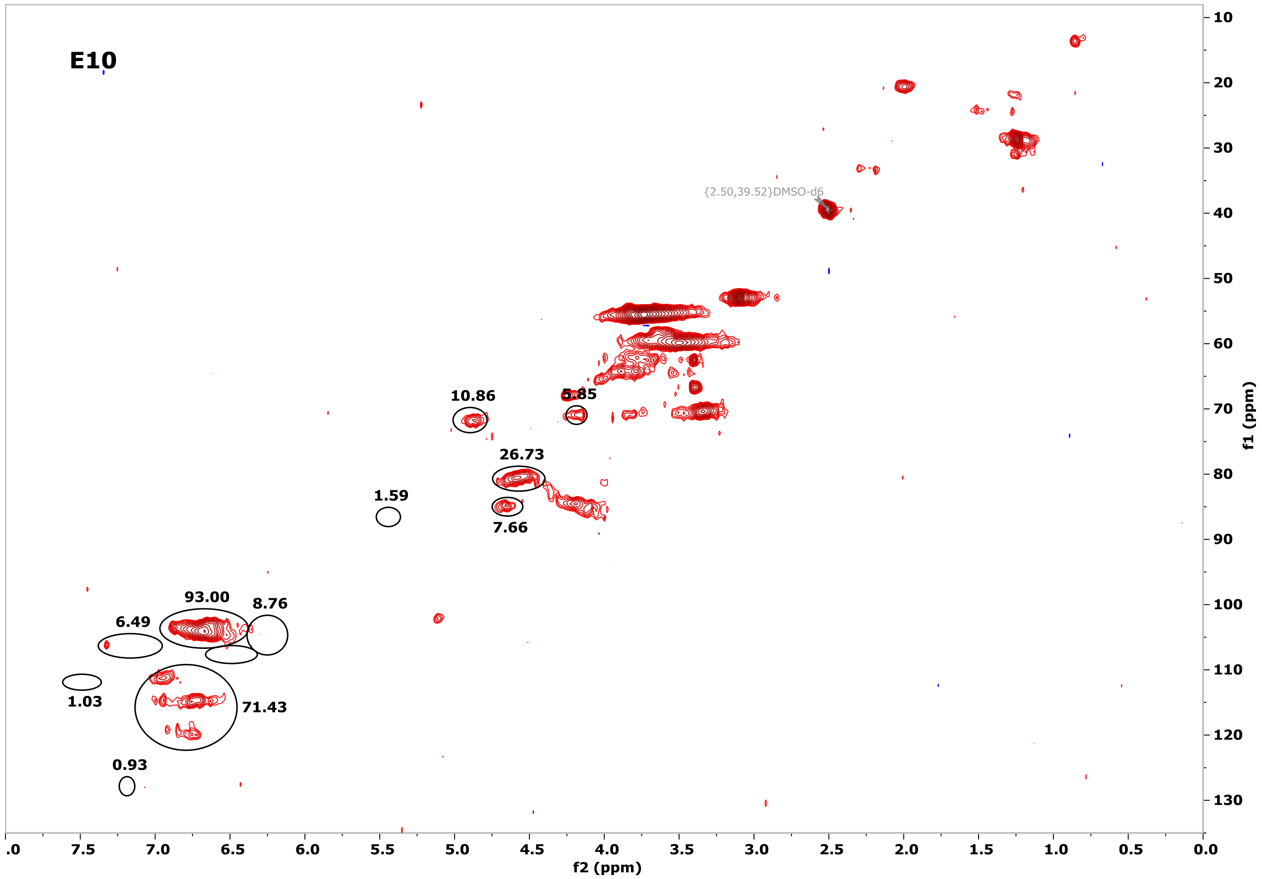

Fig. S19. 2D HSQC NMR (300 Hz, DMSO-d_6) and signal integration of lignin obtained from treating birch in the ChCh/EG/OA at 100°C for 2 h. Reaction condition: 4 g birch, 16.8 g ChCl, 3.6 g ethylene glycol, 3.12 g oxalic acid, 100°C, 2 h (See detailed reaction condition and calculating data in Tables S6, E10)

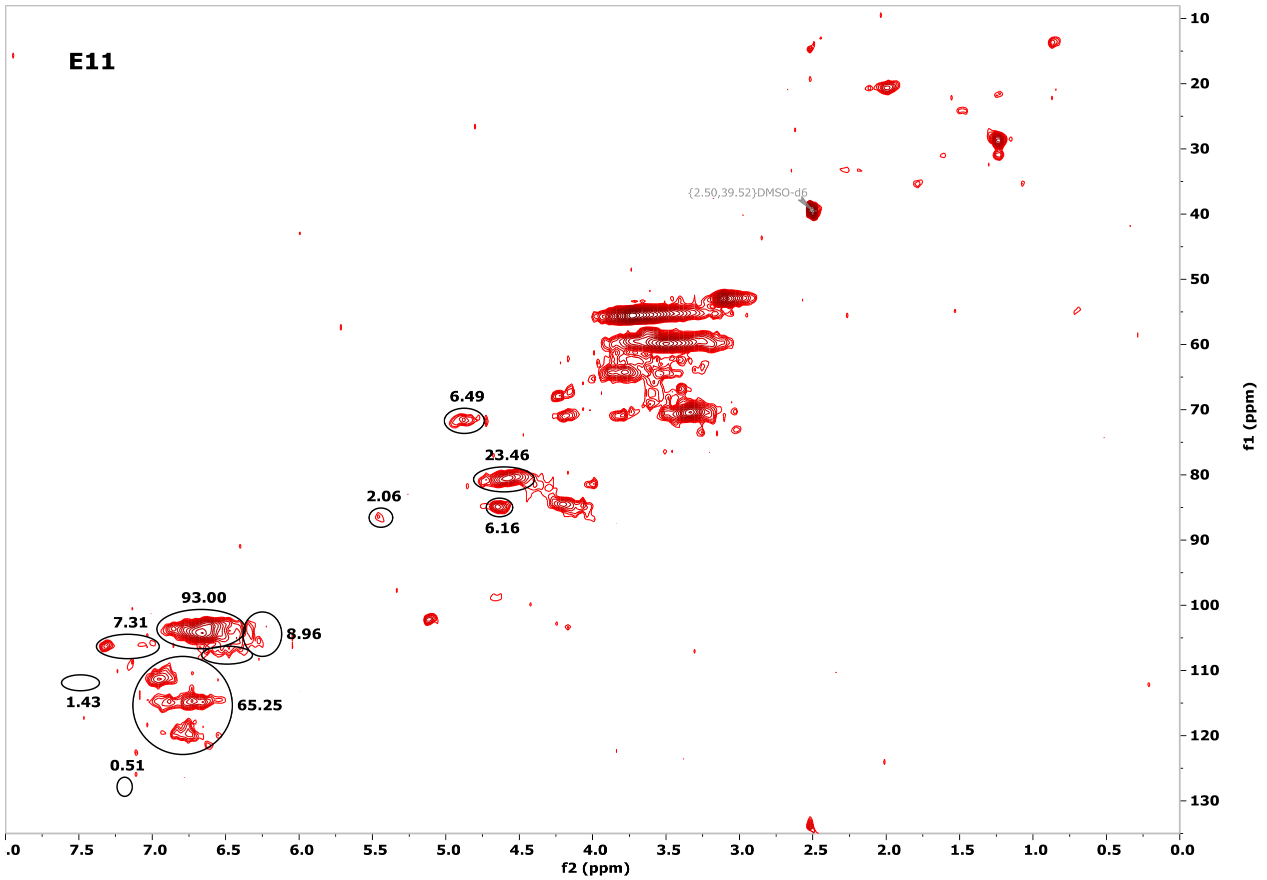

Fig. S20. 2D HSQC NMR (300 Hz, DMSO-d_6) and signal integration of lignin obtained from treating birch in the ChCh/EG/OA at 120°C for 2 h. Reaction condition: 4 g birch, 16.8 g ChCl, 3.6 g ethylene glycol, 3.12 g oxalic acid, 120°C, 2 h (See detailed reaction condition and calculating data in Table S6, E11)).

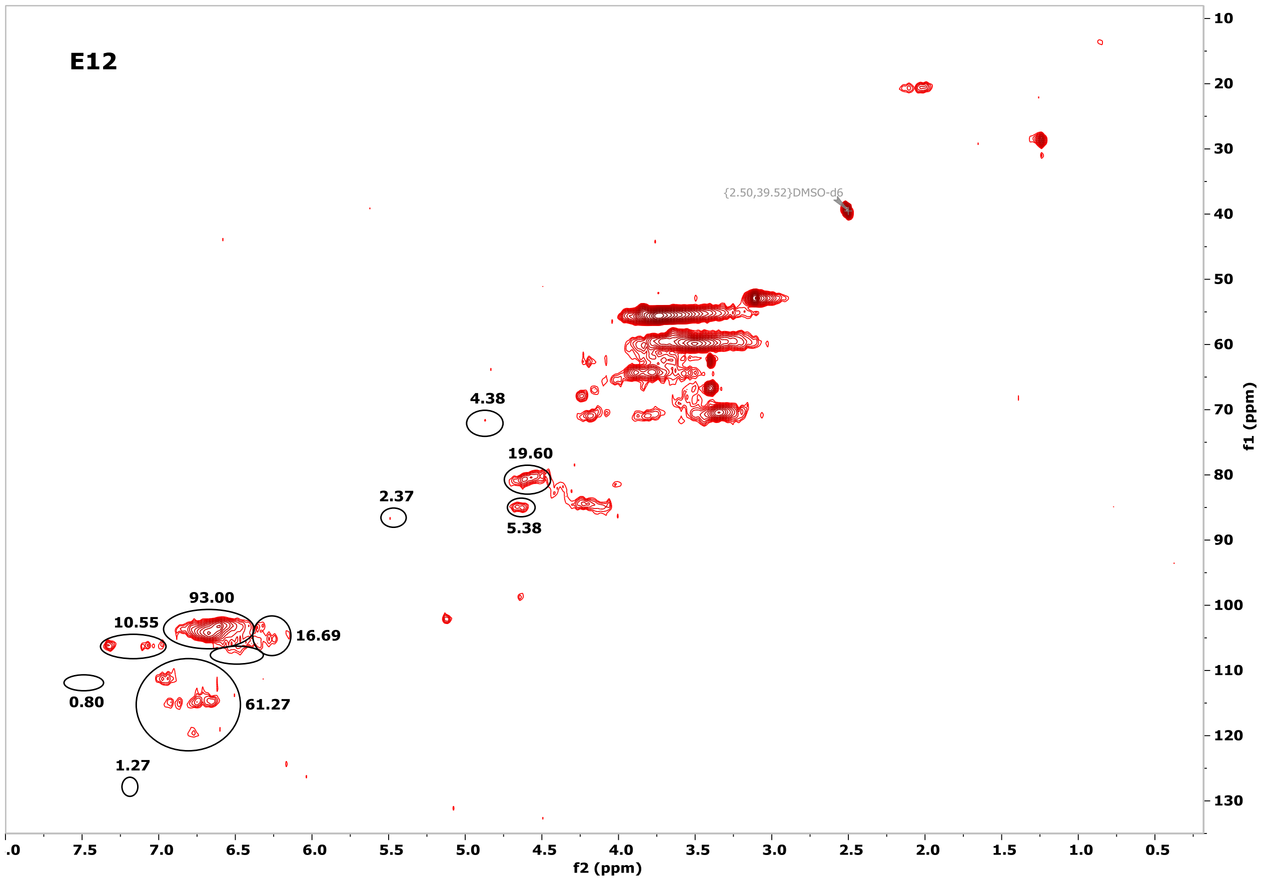

Fig. S21. 2D HSQC NMR (300 Hz, DMSO-d_6) and signal integration of lignin obtained from treating birch in the ChCh/EG/OA at 140°C for 2 h. Reaction condition: 4 g birch, 16.8 g ChCl, 3.6 g ethylene glycol, 3.12 g oxalic acid, 140°C, 2 h (See detailed reaction condition and calculating data in Table S6, E12).

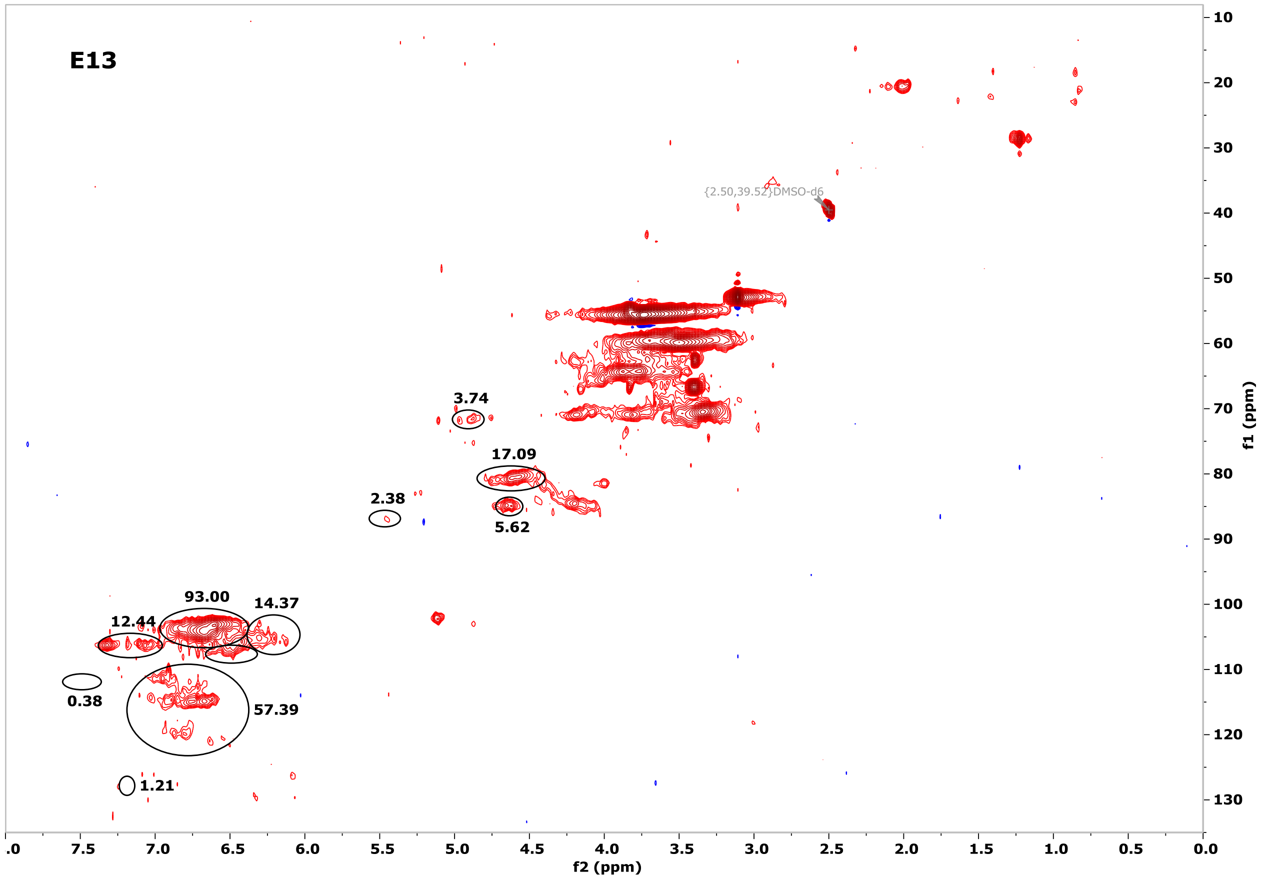

Fig. S22. 2D HSQC NMR (300 Hz, DMSO-d_6) and signal integration of lignin obtained from treating birch in the ChCh/EG/OA at 160°C for 2 h. Reaction condition: 4 g birch, 16.8 g ChCl, 3.6 g ethylene glycol, 3.12 g oxalic acid, 160°C, 2 h (See detailed reaction condition and calculating data in Table S6, E13).

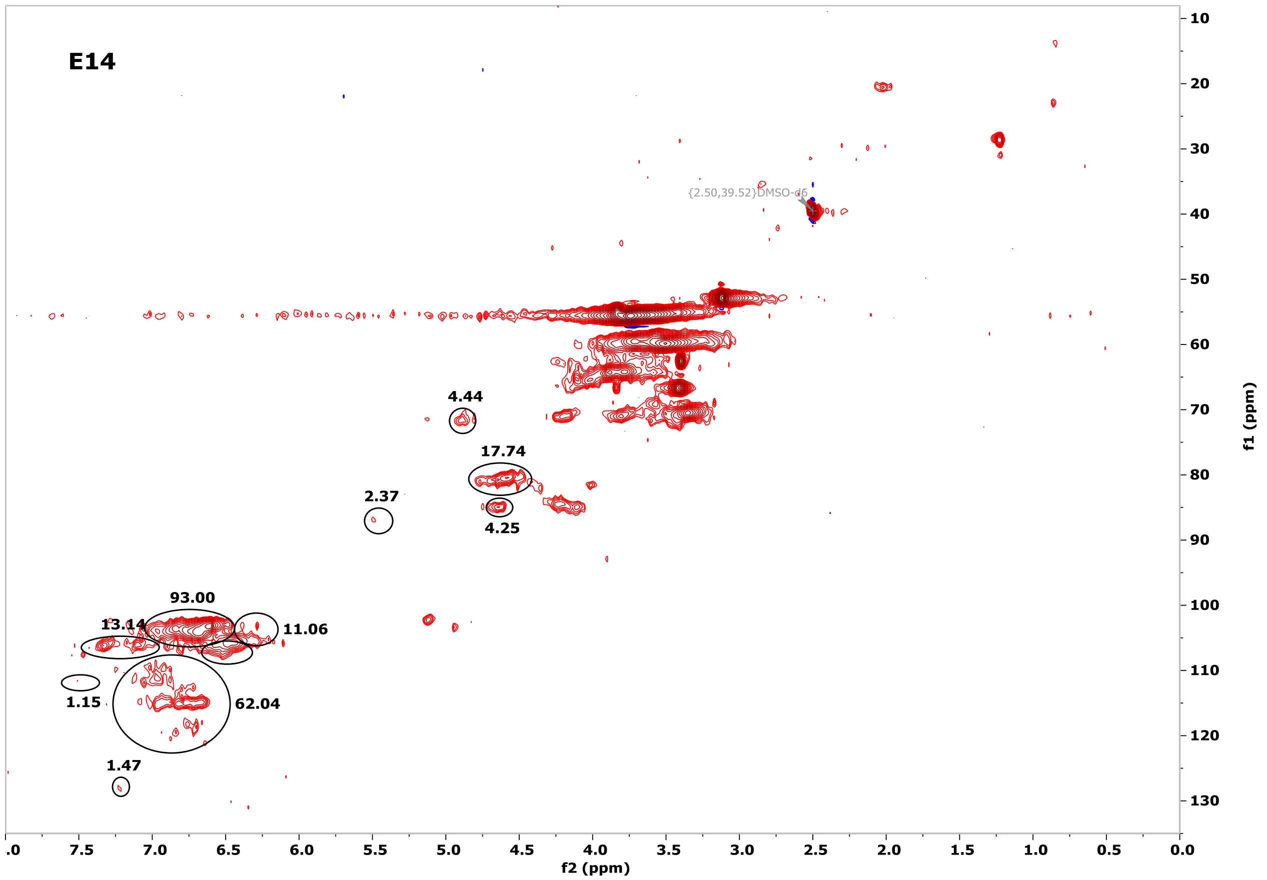

Fig. S23. 2D HSQC NMR (300 Hz, DMSO-d_6) and signal integration of lignin obtained from treating birch in the ChCh/EG/OA at 180°C for 2 h. Reaction condition: 4 g birch, 16.8 g ChCl, 3.6 g ethylene glycol, 3.12 g oxalic acid, 180°C, 2 h (See detailed reaction condition and calculating data in Table S6, E14).

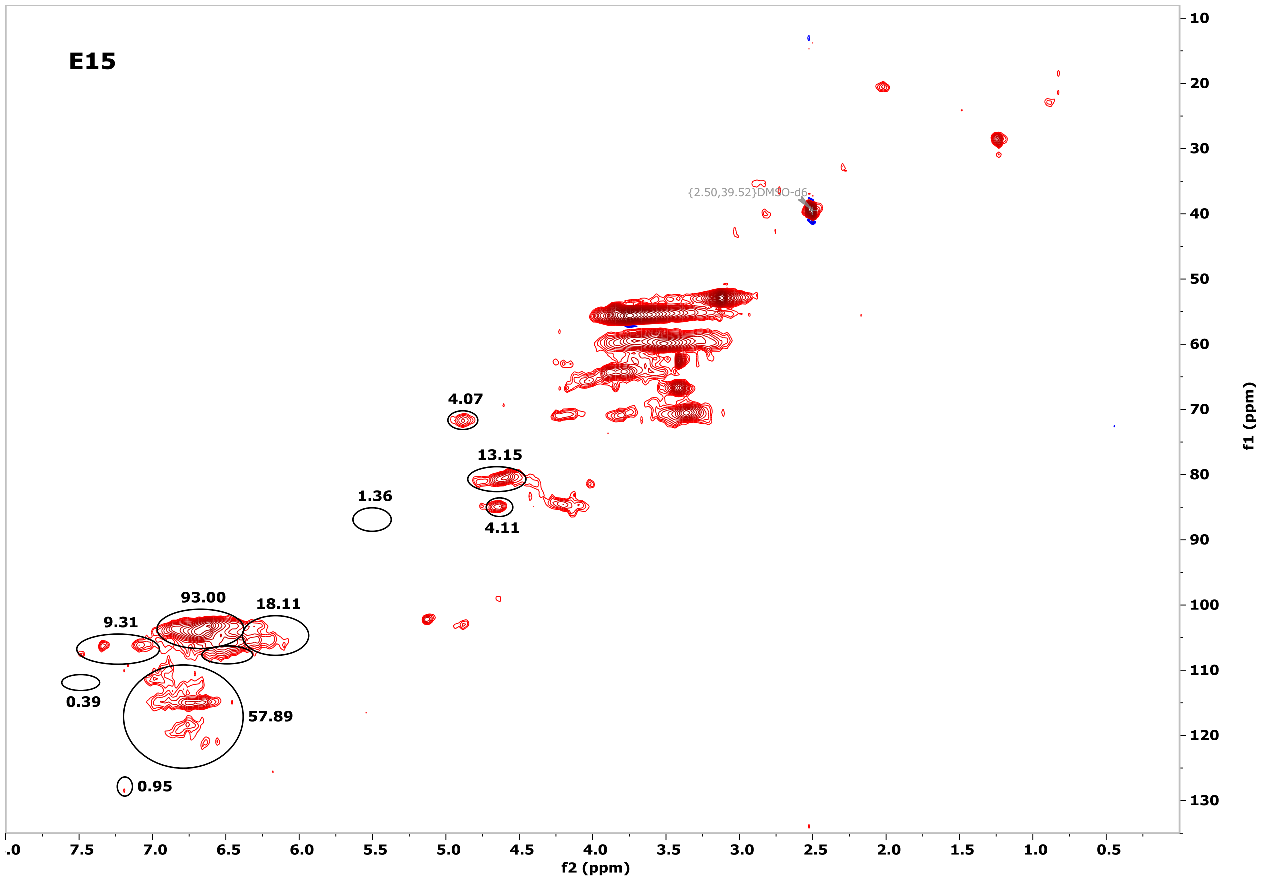

Fig. S24. 2D HSQC NMR (300 Hz, DMSO-*d*$_6$) and signal integration of lignin obtained from treating birch in the ChCh/EG/OA at 200°C for 1 h. Reaction condition: 4 g birch, 16.8 g ChCl, 3.6 g ethylene glycol, 3.12 g oxalic acid, 200°C, 1 h (See detailed reaction condition and calculating data in Table S6, E15).

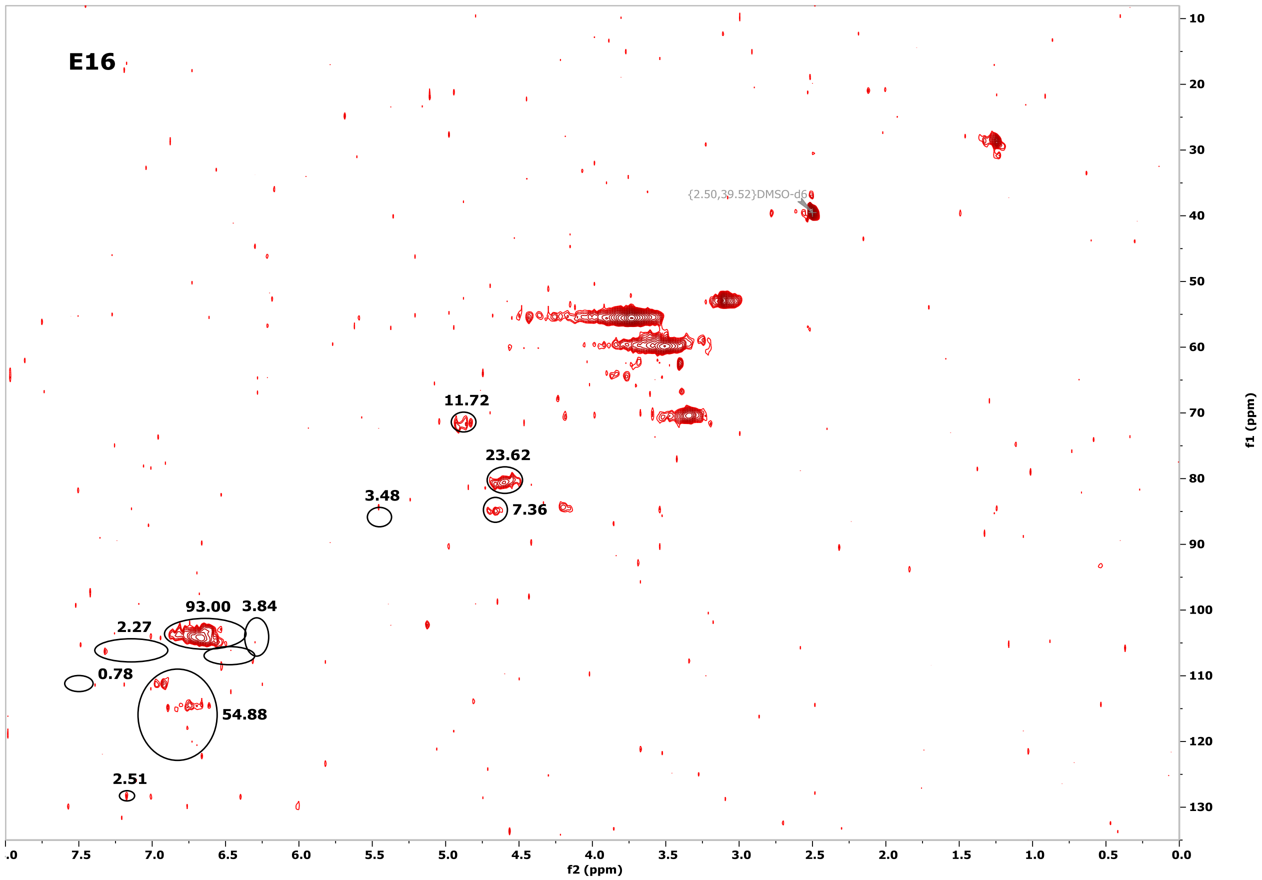

Fig. S25. 2D HSQC NMR (300 Hz, DMSO-d_6) and signal integration of lignin obtained from treating birch in the ChCh/EG/OA at 100°C for 6 h. Reaction condition: 4 g birch, 16.8 g ChCl, 3.6 g ethylene glycol, 3.12 g oxalic acid, 100°C, 6 h (See detailed reaction condition and calculating data in Table S8, E16).

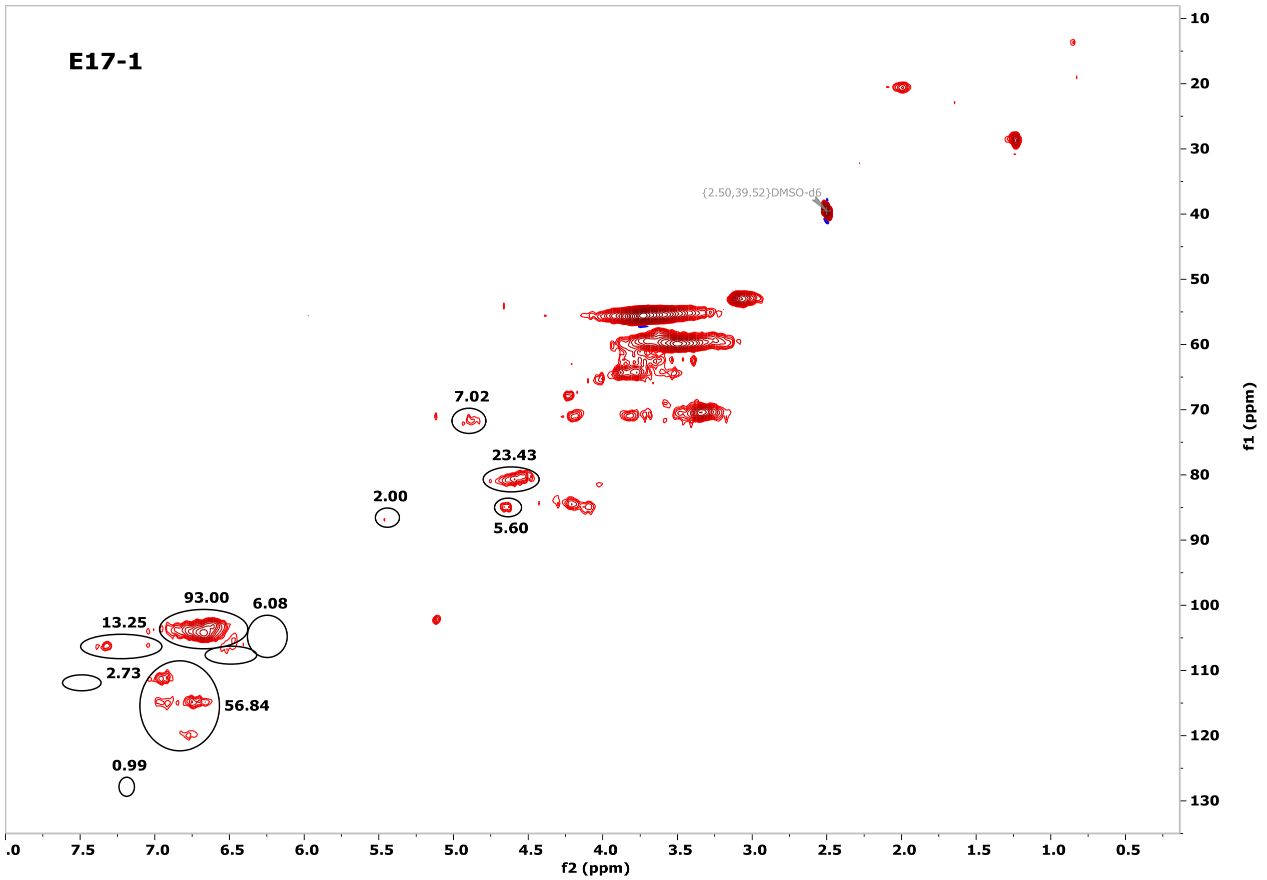

Fig. S26. 2D HSQC NMR (300 Hz, DMSO-d_6) and signal integration of lignin obtained from treating birch in the ChCh/EG/OA at 120°C for 4 h. Reaction condition: 4 g birch, 16.8 g ChCl, 3.6 g ethylene glycol, 3.12 g oxalic acid, 120°C, 4 h (See detailed reaction condition and calculating data in Table S8, E17_1).

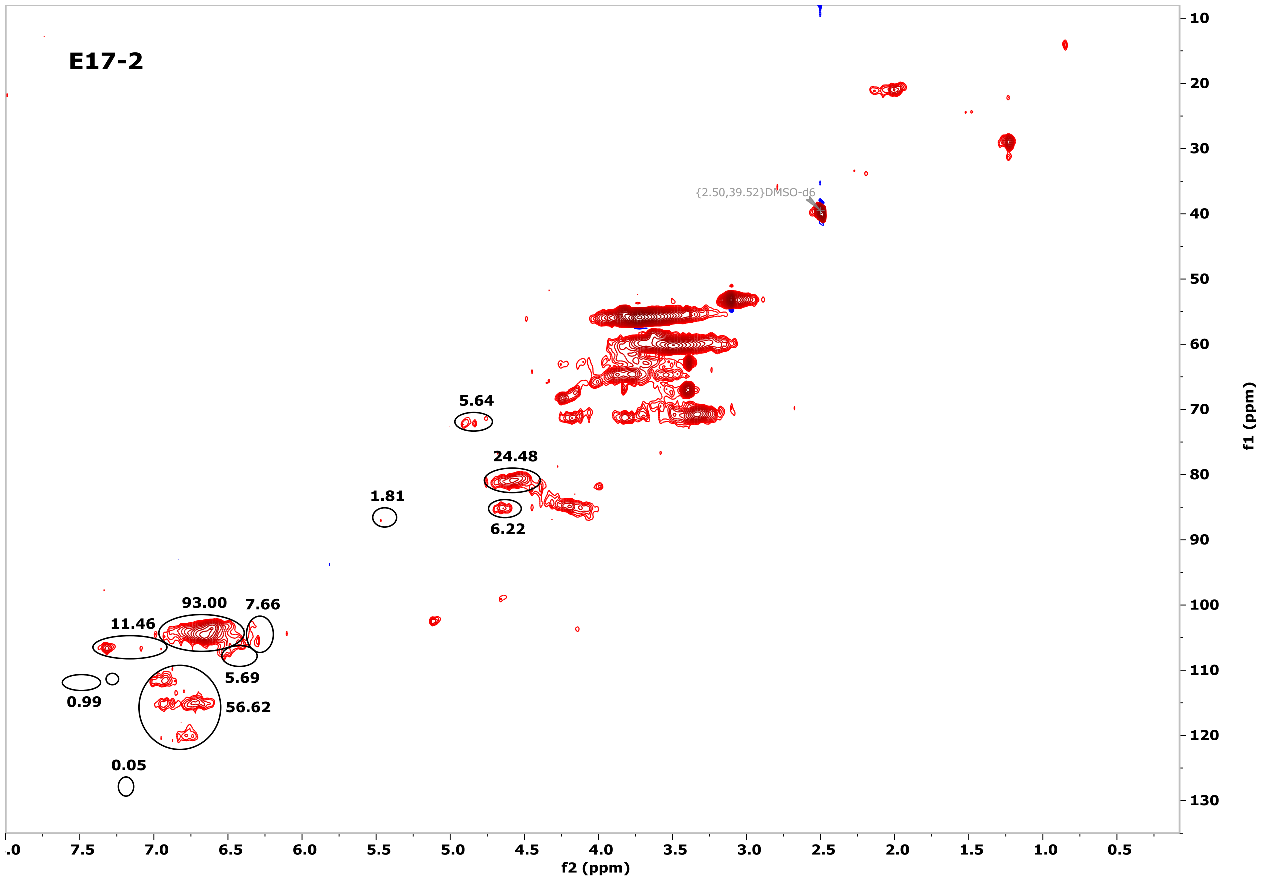

Fig. S27. 2D HSQC NMR (300 Hz, DMSO-d_6) and signal integration of lignin obtained from treating birch in the ChCh/EG/OA at 120°C for 4 h. Reaction condition: 4 g birch, 16.8 g ChCl, 3.6 g ethylene glycol, 3.12 g oxalic acid, 120°C, 4 h (See detailed reaction condition and calculating data in Table S8, E17_2).

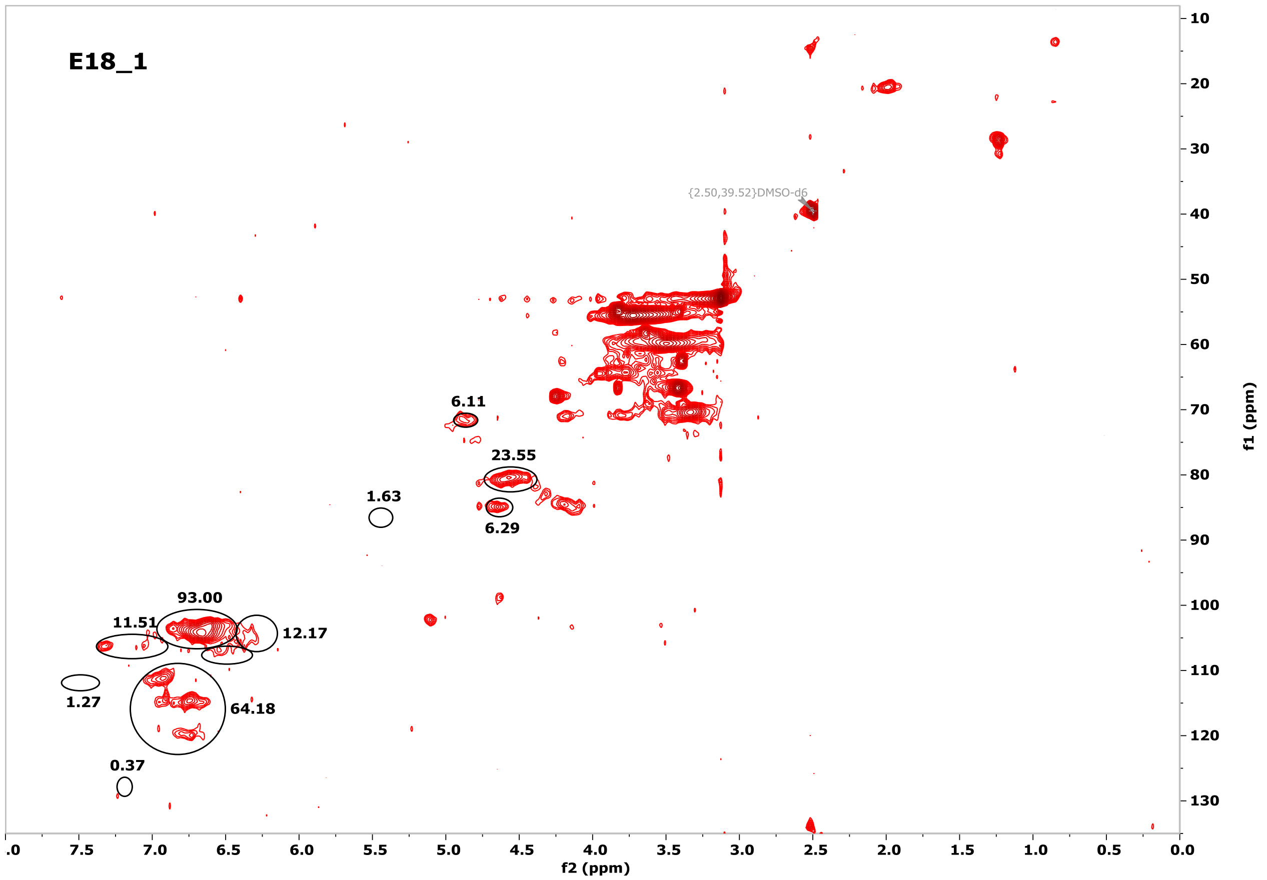

Fig. S28. 2D HSQC NMR (300 Hz, DMSO-d_6) and signal integration of lignin obtained from treating birch in the ChCh/EG/OA at 120°C for 6 h. Reaction condition: 4 g birch, 16.8 g ChCl, 3.6 g ethylene glycol, 3.12 g oxalic acid, 120°C, 6 h (See detailed reaction condition and calculating data in Table S8, E18_1).

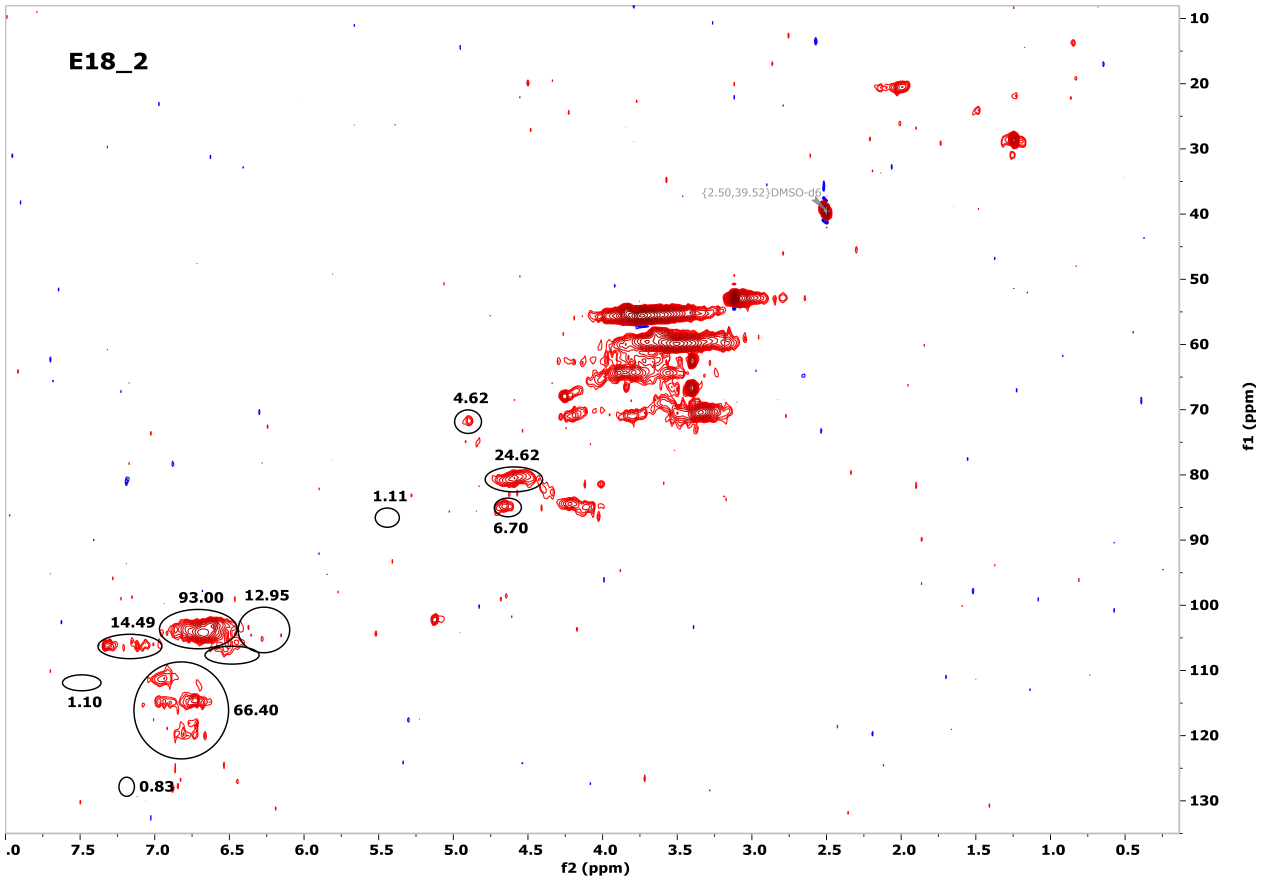

Fig. S29. 2D HSQC NMR (300 Hz, DMSO-*d*$_6$) and signal integration of lignin obtained from treating birch in the ChCh/EG/OA at 120°C for 6 h. Reaction condition: 4 g birch, 16.8 g ChCl, 3.6 g ethylene glycol, 3.12 g oxalic acid, 120°C, 6 h (See detailed reaction condition and calculating data in Table S8, E18_2).

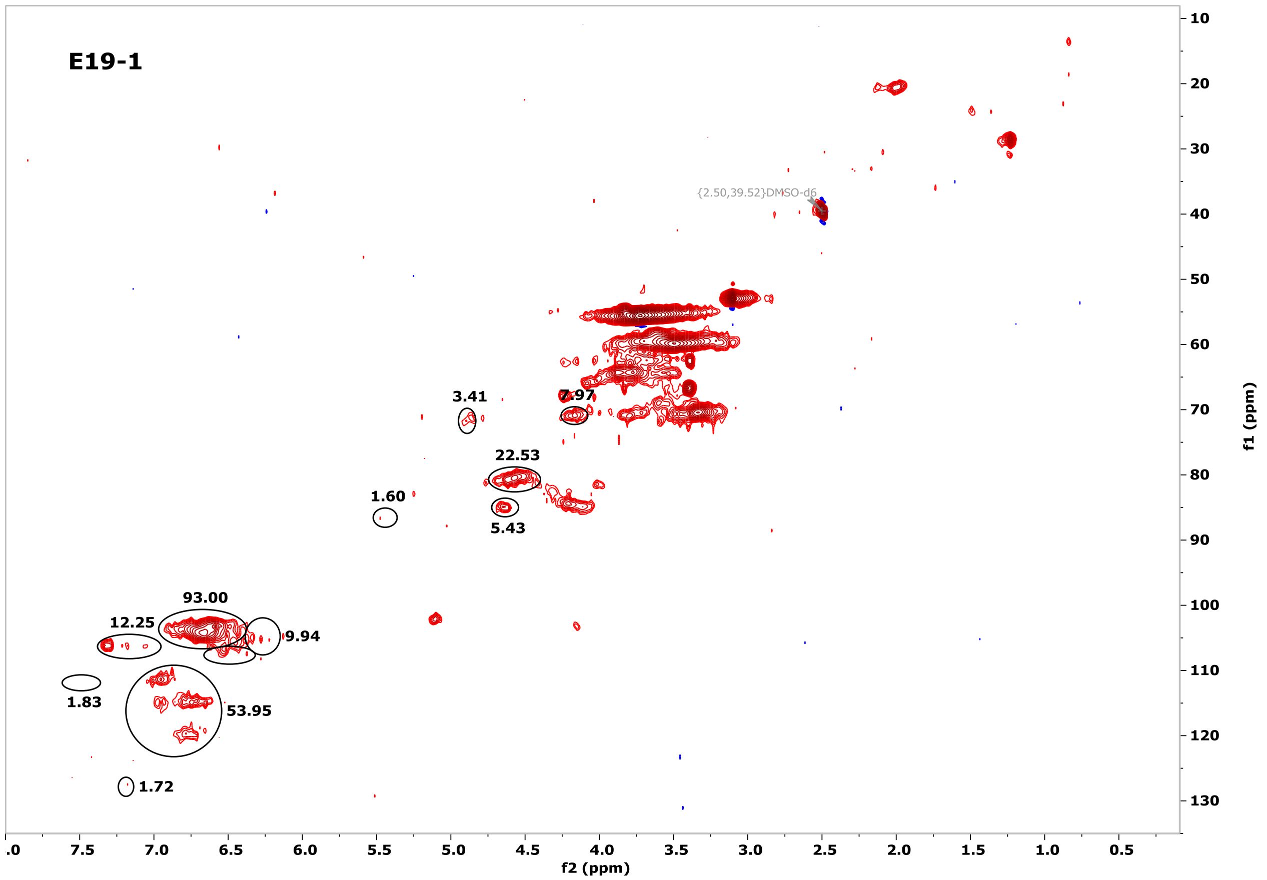

Fig. S30. 2D HSQC NMR (300 Hz, DMSO-d_6) and signal integration of lignin obtained from treating birch in the ChCh/EG/OA at 120°C for 12 h. Reaction condition: 4 g birch, 16.8 g ChCl, 3.6 g ethylene glycol, 3.12 g oxalic acid, 120°C, 12 h (See detailed reaction condition and calculating data in Table S8, E19_1).

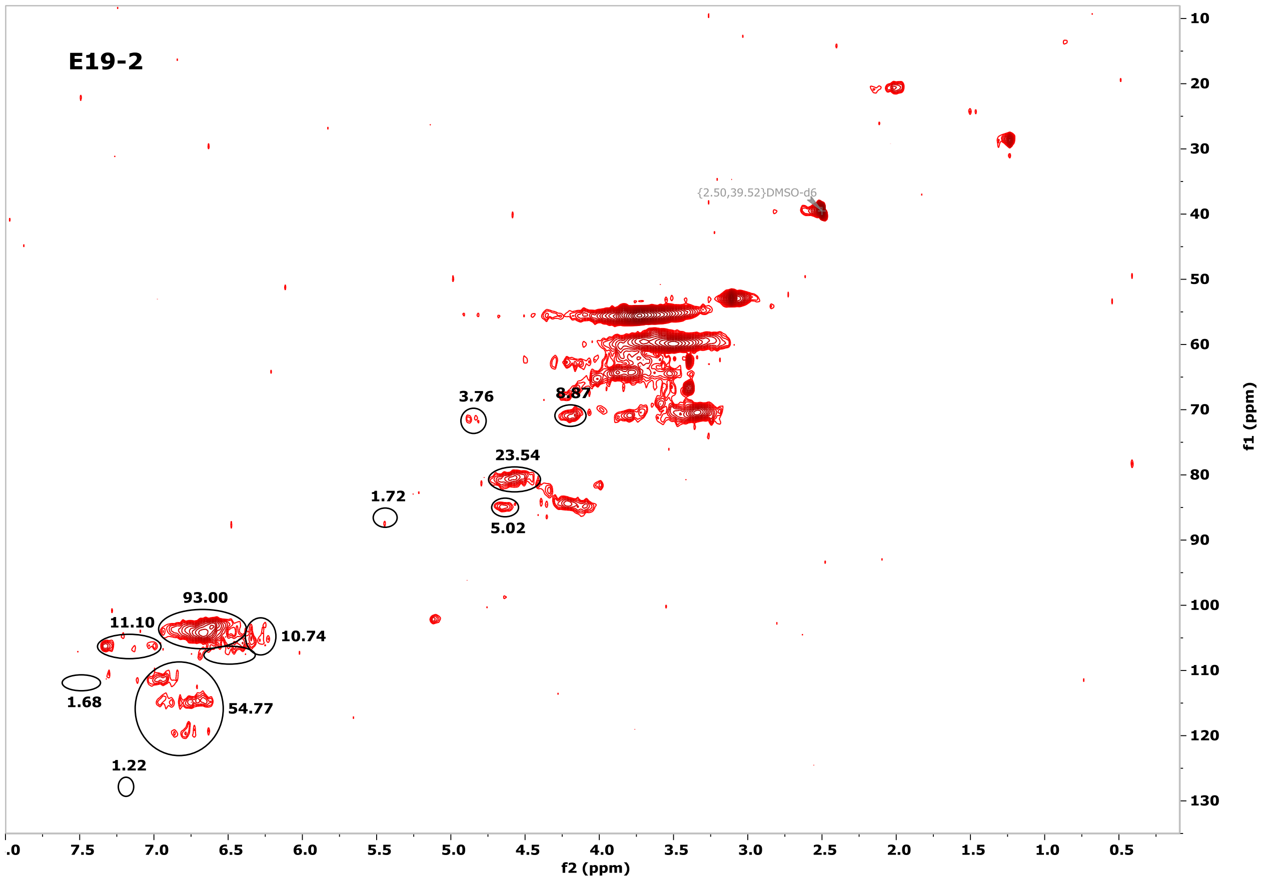

Fig. S31. 2D HSQC NMR (300 Hz, DMSO-d_6) and signal integration of lignin obtained from treating birch in the ChCh/EG/OA at 120°C for 2h. Reaction condition: 4 g birch, 16.8 g ChCl, 3.6 g ethylene glycol, 3.12 g oxalic acid, 120°C, 12 h (See detailed reaction condition and calculating data in Table S8, E19_2).

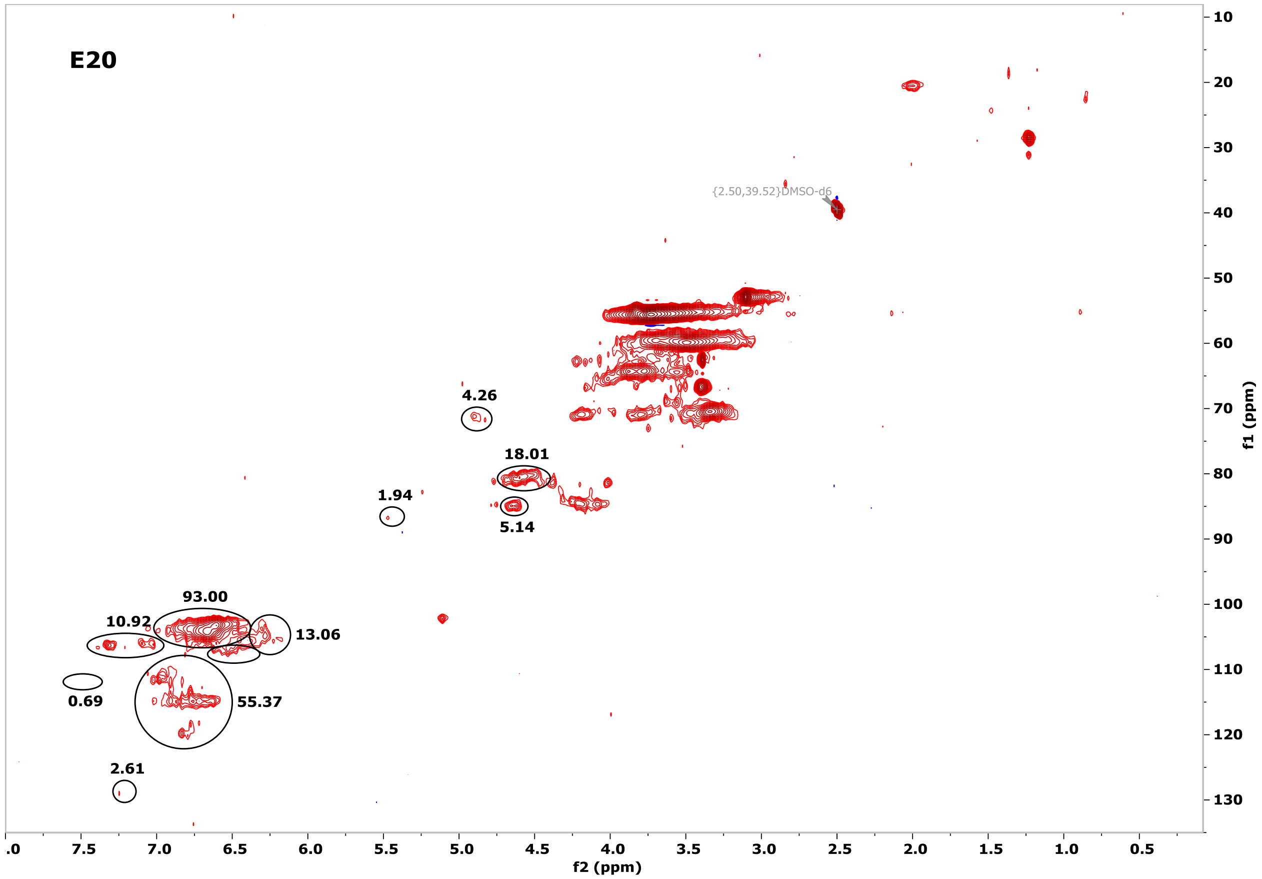

Fig. S32. 2D HSQC NMR (300 Hz, DMSO-d_6) and signal integration of lignin obtained from treating birch in the ChCh/EG/OA at 140°C for 6 h. Reaction condition: 4 g birch, 16.8 g ChCl, 3.6 g ethylene glycol, 3.12 g oxalic acid, 140°C, 6 h (See detailed reaction condition and calculating data in Table S8, E20).

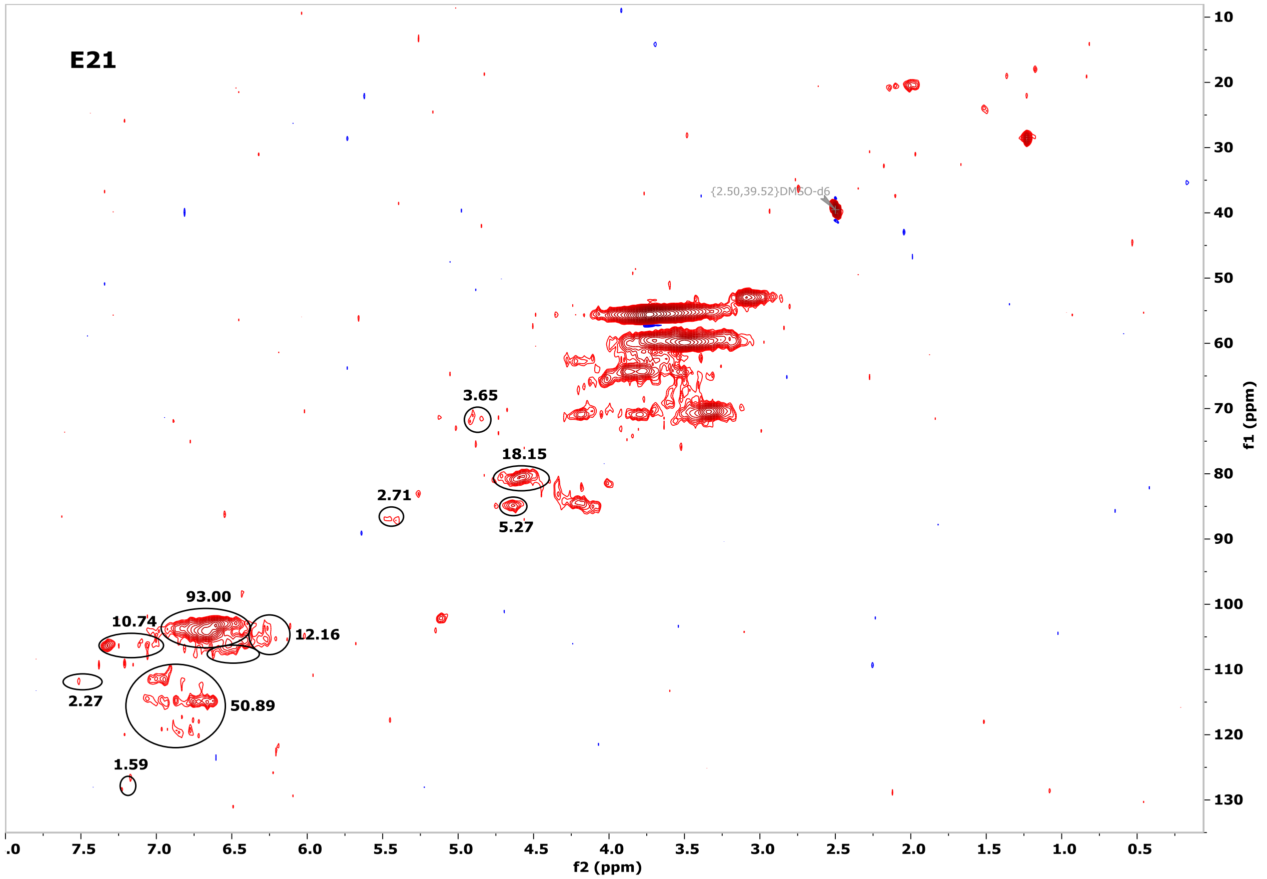

Fig. S33. 2D HSQC NMR (300 Hz, DMSO-d_6) and signal integration of lignin obtained from treating birch in the ChCh/EG/OA at 140°C for 12 h. Reaction condition: 4 g birch, 16.8 g ChCl, 3.6 g ethylene glycol, 3.12 g oxalic acid, 140°C, 12 h (See detailed reaction condition and calculating data in Table S8, E21).

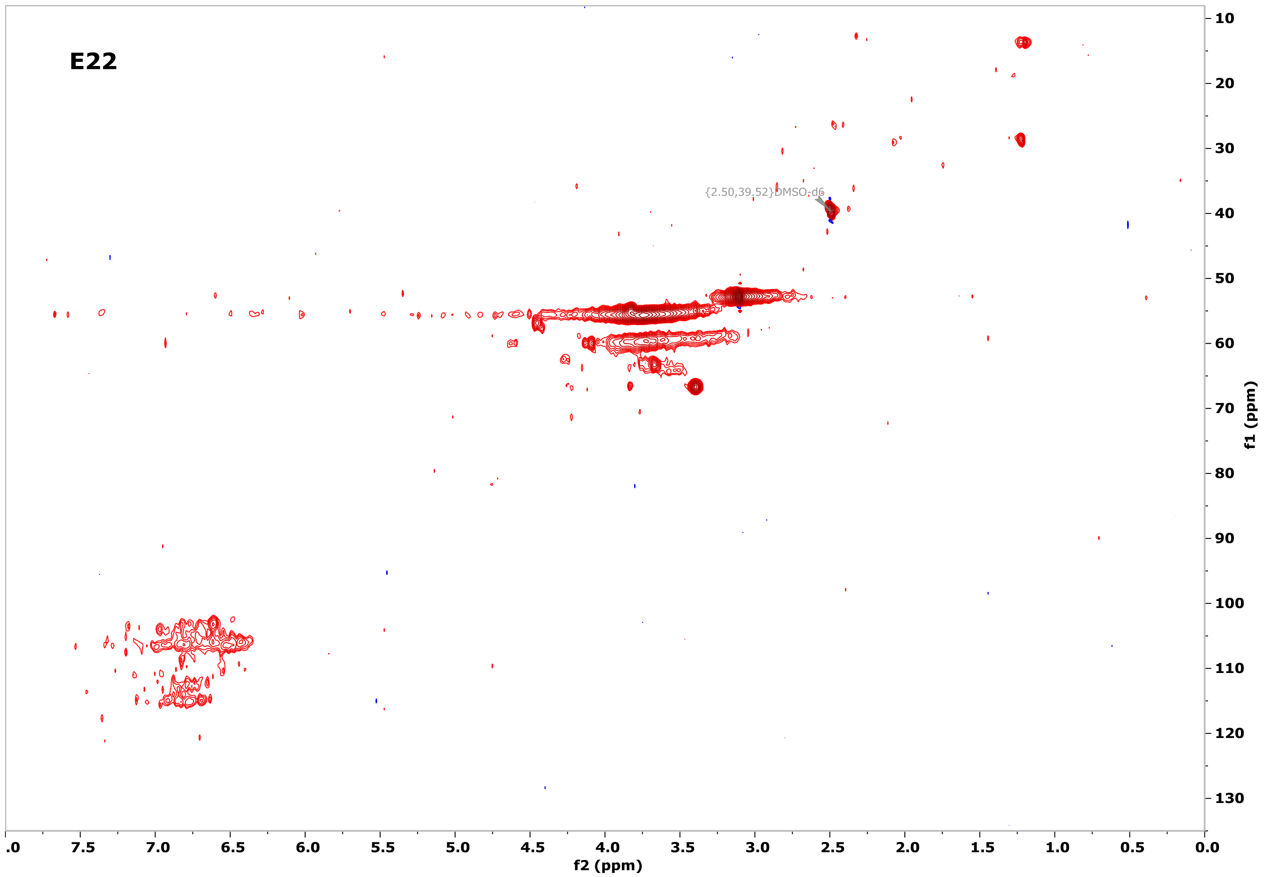

Fig. S34. 2D HSQC NMR (300 Hz, DMSO-d_6) and signal integration of lignin obtained from treating birch in the ChCh/EG/OA at 120°C for 4 h. Reaction condition: 4 g birch, 20.85 g ChCl, 0 g ethylene glycol, 13.48 g oxalic acid, 120°C, 4 h (See detailed reaction condition and calculating data in Table S10, E22)

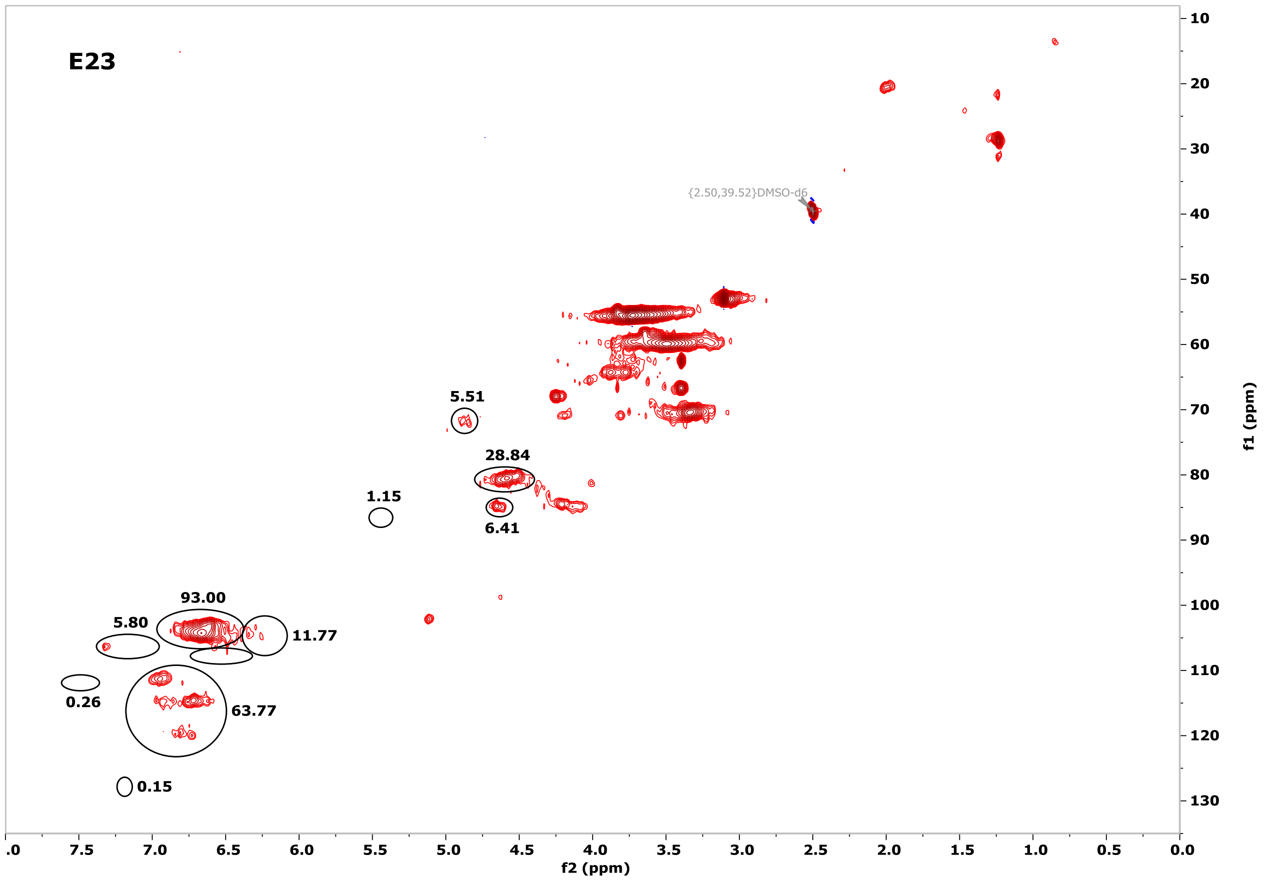

Fig. S35. 2D HSQC NMR (300 Hz, DMSO-d_6) and signal integration of lignin obtained from treating birch in the ChCh/EG/OA at 120°C for 4 h. Reaction condition: 4 g birch, 11.23 g ChCl, 19.86 g ethylene glycol, 3.12 g oxalic acid, 120°C, 4 h (See detailed reaction condition and calculating data in Table S10, E23).

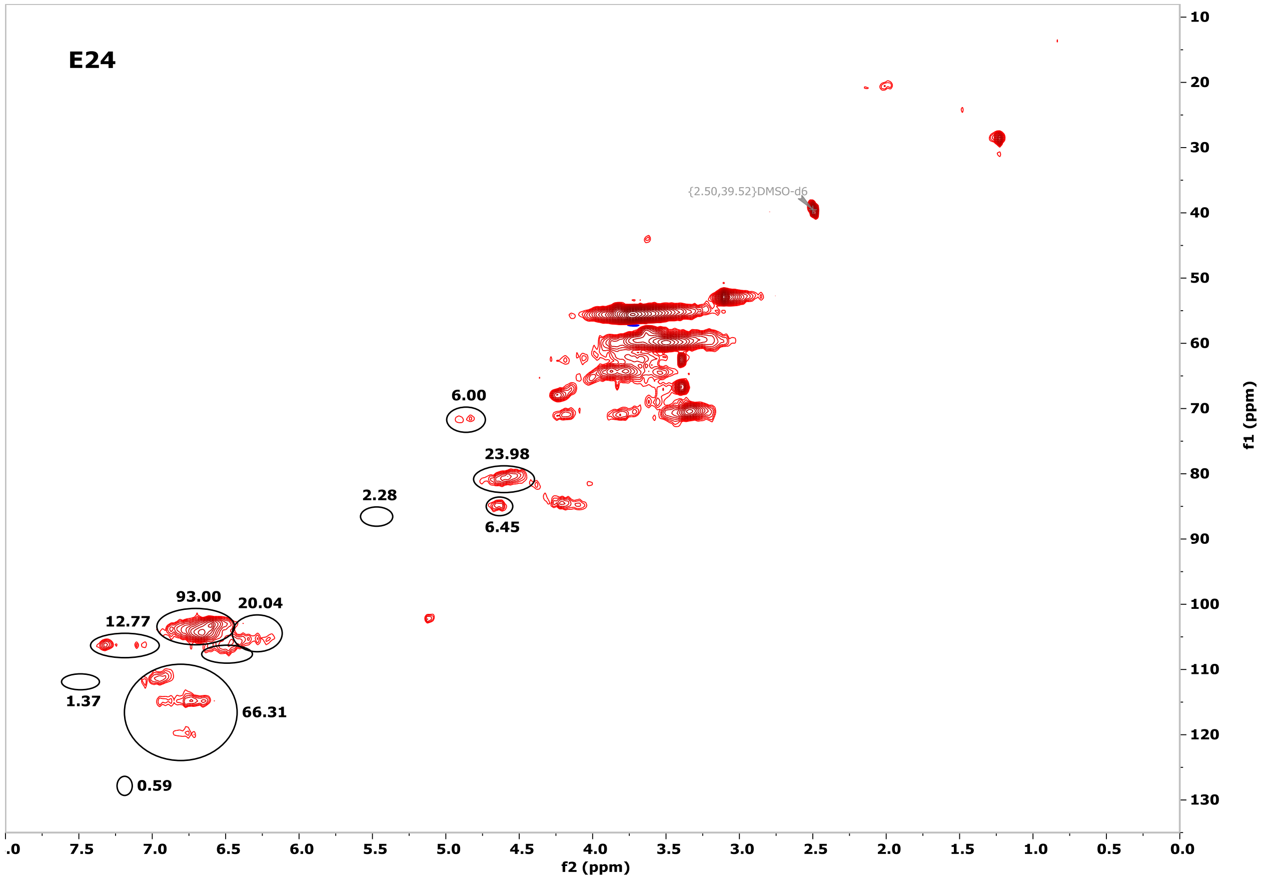

Fig. S36. 2D HSQC NMR (300 Hz, DMSO-d_6) and signal integration of lignin obtained from treating birch in the ChCh/EG/OA at 120°C for 4 h. Reaction condition: 4 g birch, 15.1 g ChCl, 13.4 g ethylene glycol, 5.7 g oxalic acid, 120°C, 4 h (See detailed reaction condition and calculating data in Table S10).

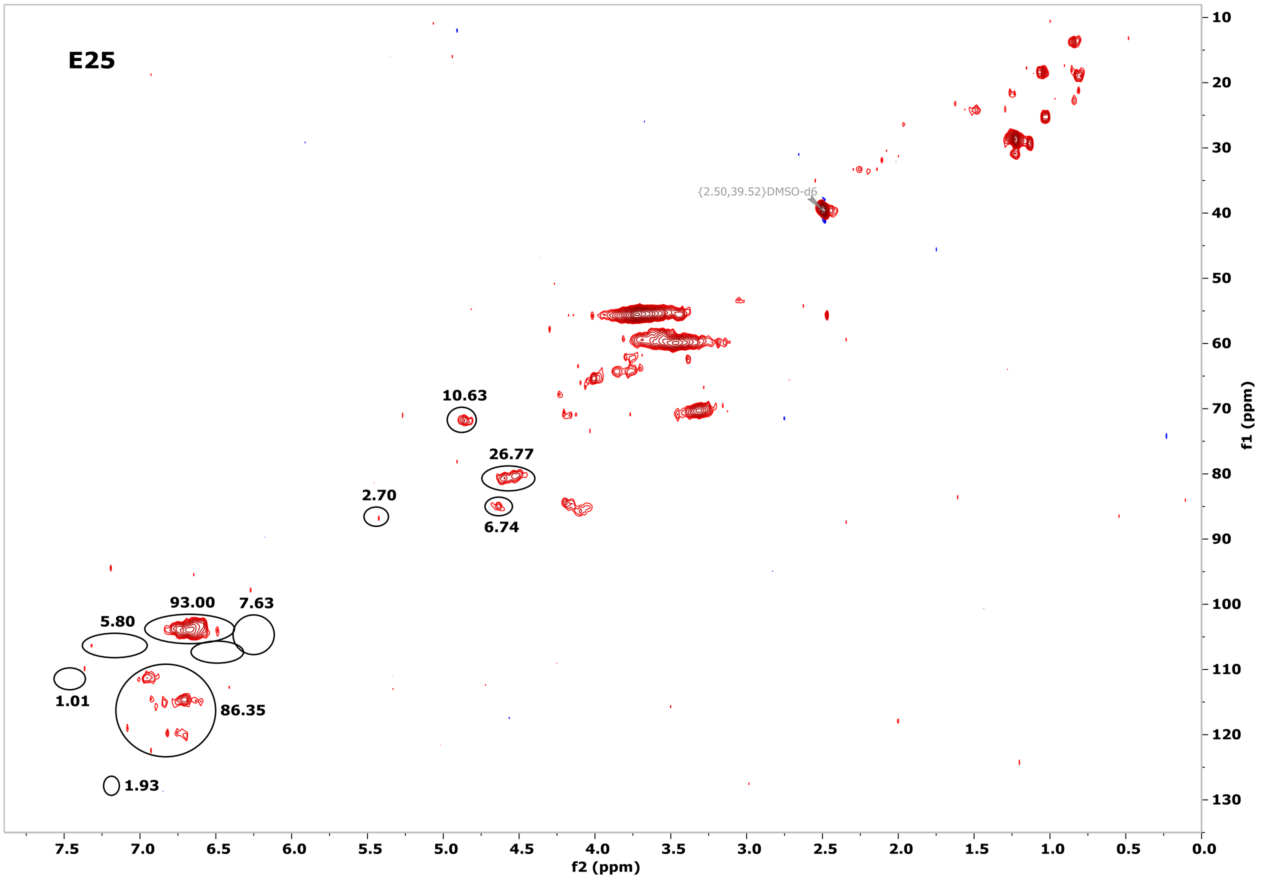

Fig. S37. 2D HSQC NMR (300 Hz, DMSO-d_6) and signal integration of lignin obtained from treating birch in the ChCh/EG/OA at 120°C for 4 h. Reaction condition: 4 g birch, 0 g ChCl, 31.2 g ethylene glycol, 3.12 g oxalic acid, 120°C, 4 h (See detailed reaction condition and calculating data in Table S10, E25).

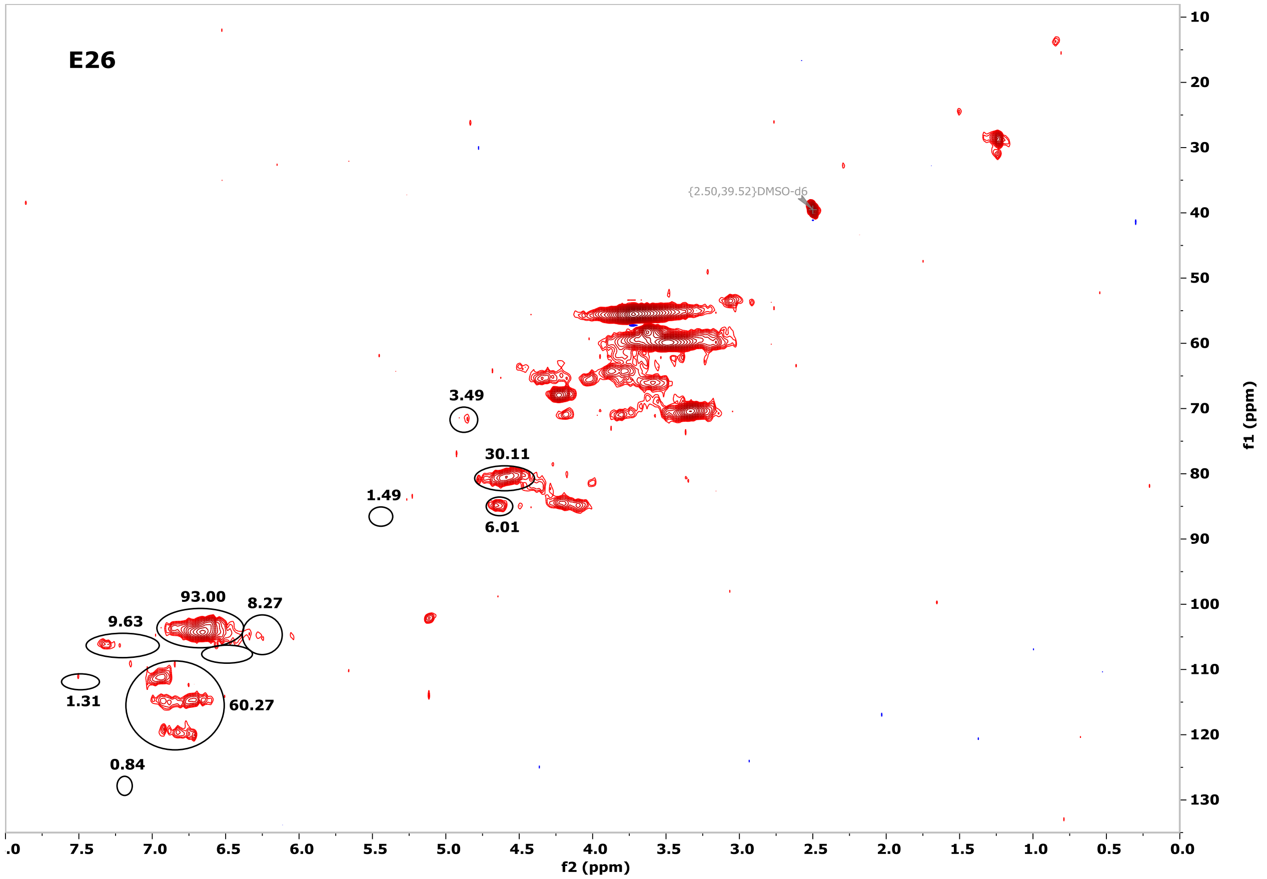

Fig. S38. 2D HSQC NMR (300 Hz, DMSO-d_6) and signal integration of lignin obtained from treating birch in the ChCh/EG/OA at 100°C for 12 h. Reaction condition: 4 g birch, 0 g ChCl, 22.88 g ethylene glycol, 11.44 g oxalic acid, 100°C, 12 h (See detailed reaction condition and calculating data in Table S10, E26).

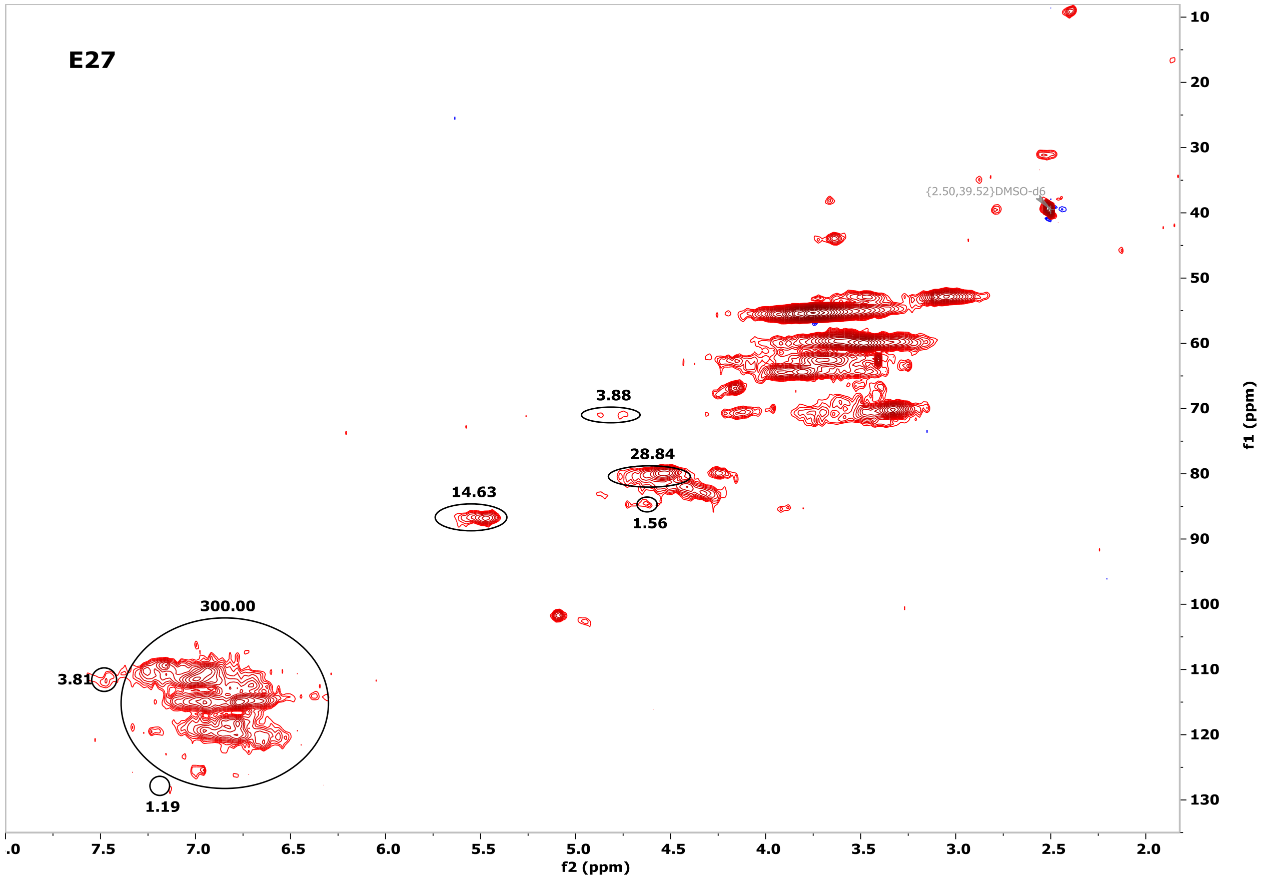

Fig. S39. 2D HSQC NMR (300 Hz, DMSO-d_6) and signal integration of lignin obtained from treating pine in the ChCh/EG/OA at 140°C for 2 h. Reaction condition: 4 g pine, 16.8 g ChCl, 14.4 g ethylene glycol, 3.12 g oxalic acid, 140°C, 2 h (See detailed reaction condition and calculating data in Table S12, E27).

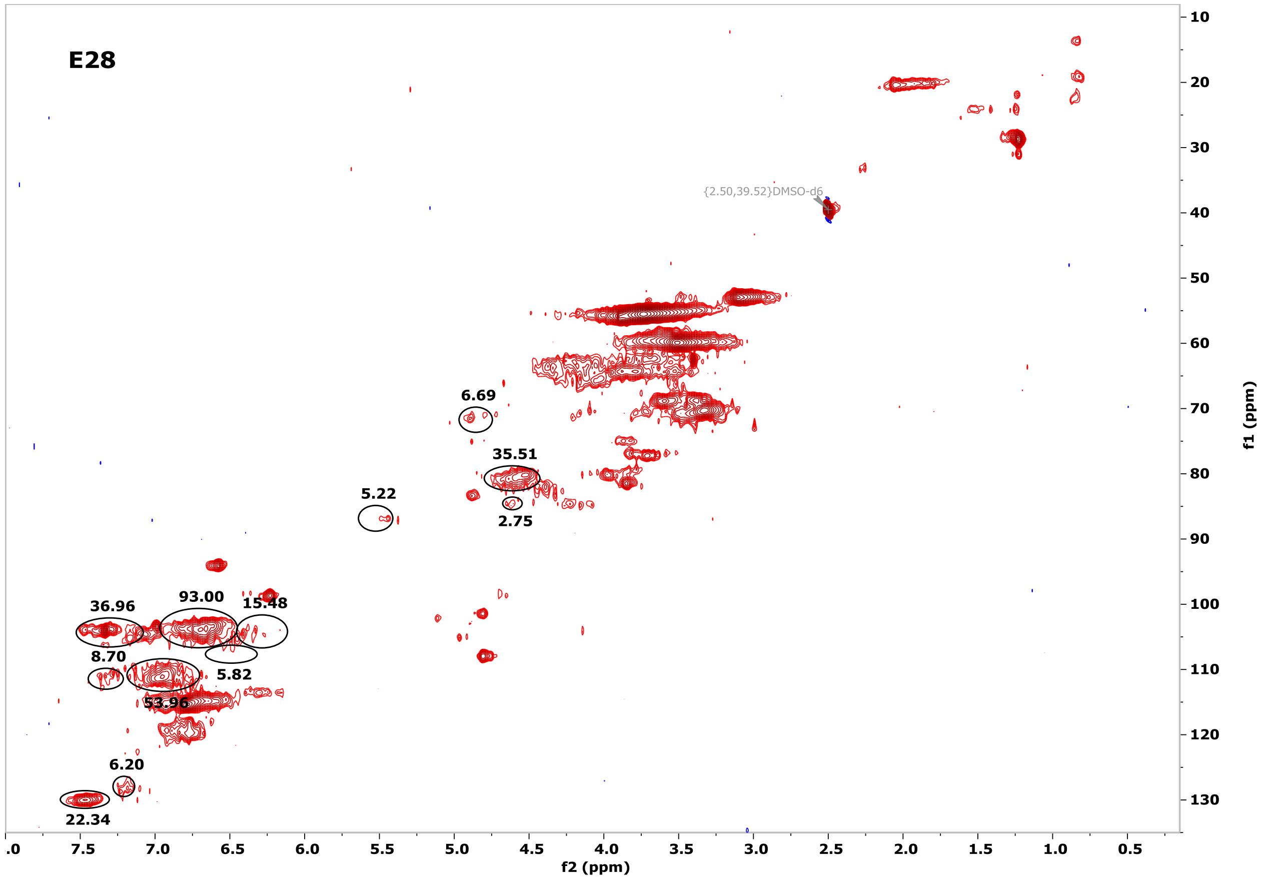

Fig. S40. 2D HSQC NMR (300 Hz, DMSO-d_6) and signal integration of lignin obtained from treating wheat in the ChCh/EG/OA at 140°C for 2 h. Reaction condition: 4 g wheat, 16.8 g ChCl, 14.4 g ethylene glycol, 3.12 g oxalic acid, 140°C, 2 h (See detailed reaction condition and calculating data in Table S12, E28).

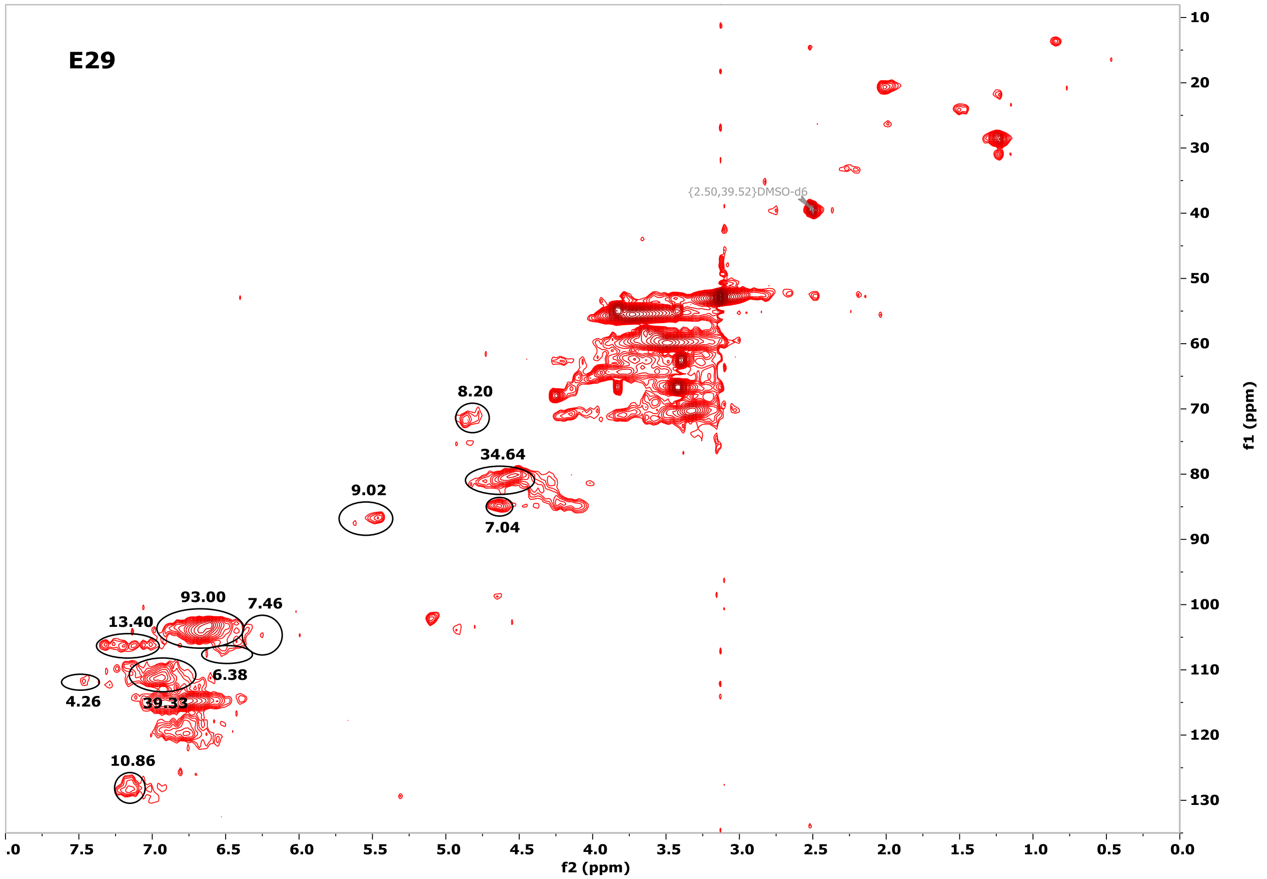

Fig. S41. 2D HSQC NMR (300 Hz, DMSO-*d*$_6$) and signal integration of lignin obtained from treating walnut shell in the ChCh/EG/OA at 140°C for 2 h. Reaction condition: 4 g sugarcane, 16.8 g ChCl, 14.4 g ethylene glycol, 3.12 g oxalic acid, 140°C, 2 h (See detailed reaction condition and calculating data in Table S12, E29).

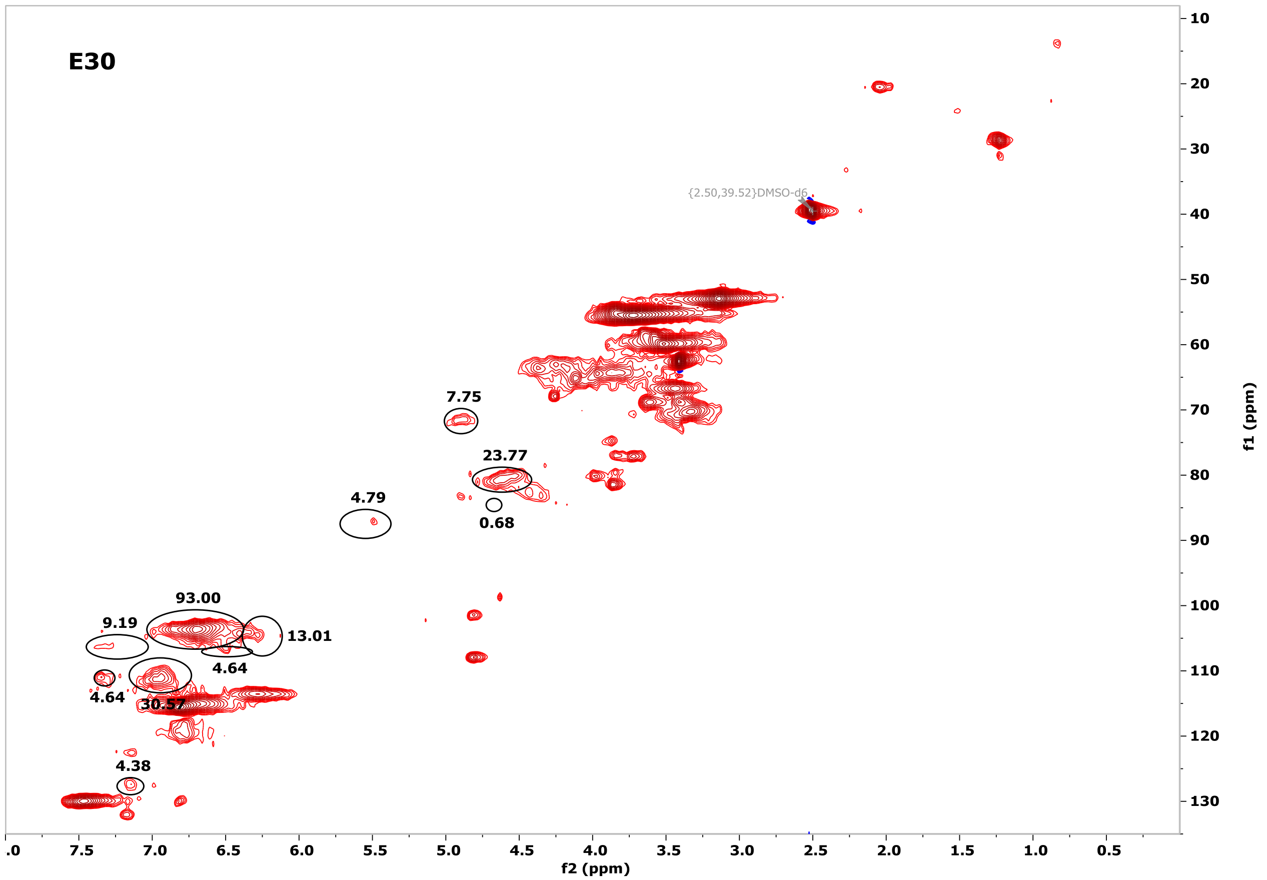

Fig. S42. 2D HSQC NMR (300 Hz, DMSO-d_6) and signal integration of lignin obtained from treating sugarcabe bagaase in the ChCh/EG/OA at 140°C for 2 h. Reaction condition: 4 g walnut, 16.8 g ChCl, 14.4 g ethylene glycol, 3.12 g oxalic acid, 140°C, 2 h (See detailed reaction condition and calculating data in Table S12, E30).

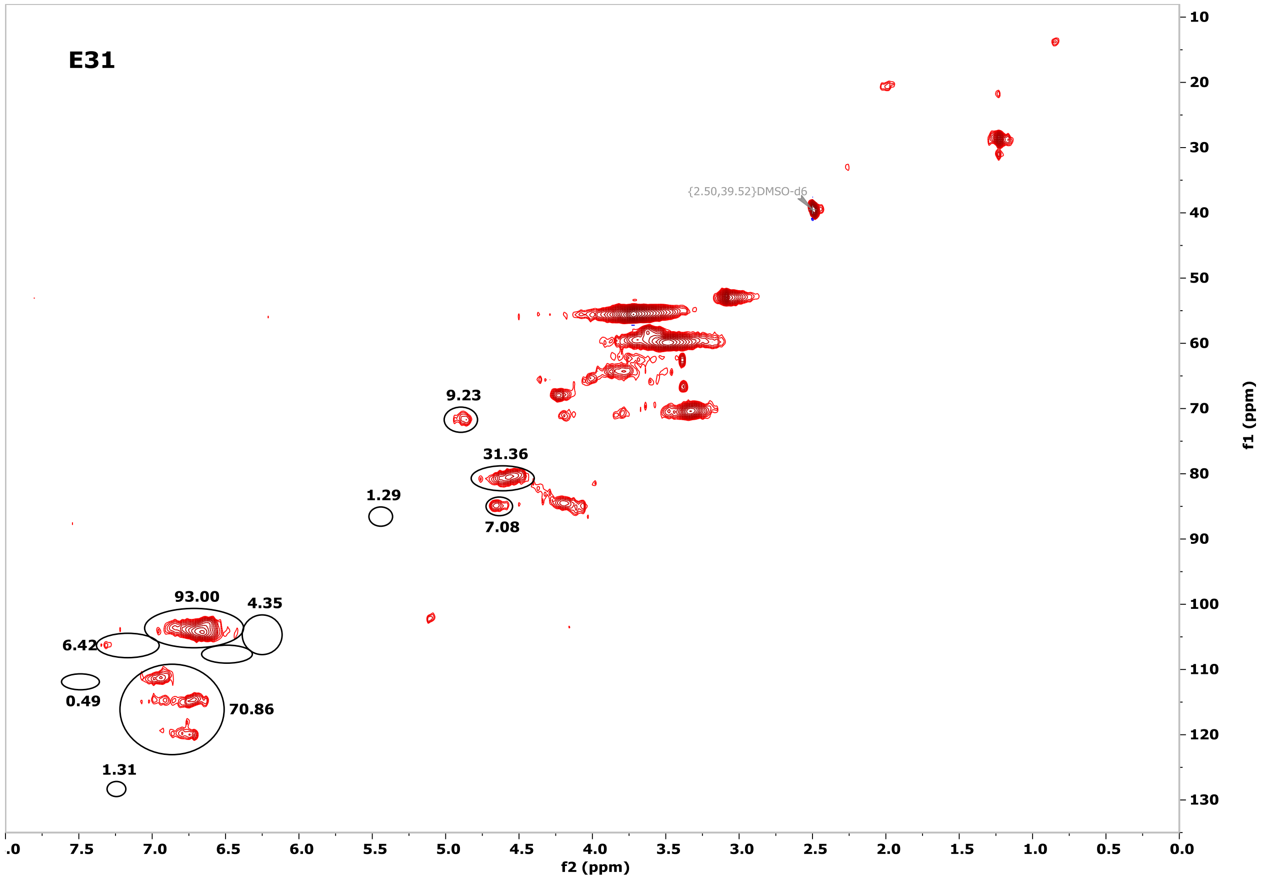

Fig. S43. 2D HSQC NMR (300 Hz, DMSO-d_6) and signal integration of lignin obtained from treating birch in the ChCh/EG/OA at 80°C for 24 h. Reaction condition: 4 g birch (ball milled), 16.8 g ChCl, 14.4 g ethylene glycol, 3.12 g oxalic acid, 80°C, 24 h (See detailed reaction condition and calculating data in Table S14, E31).

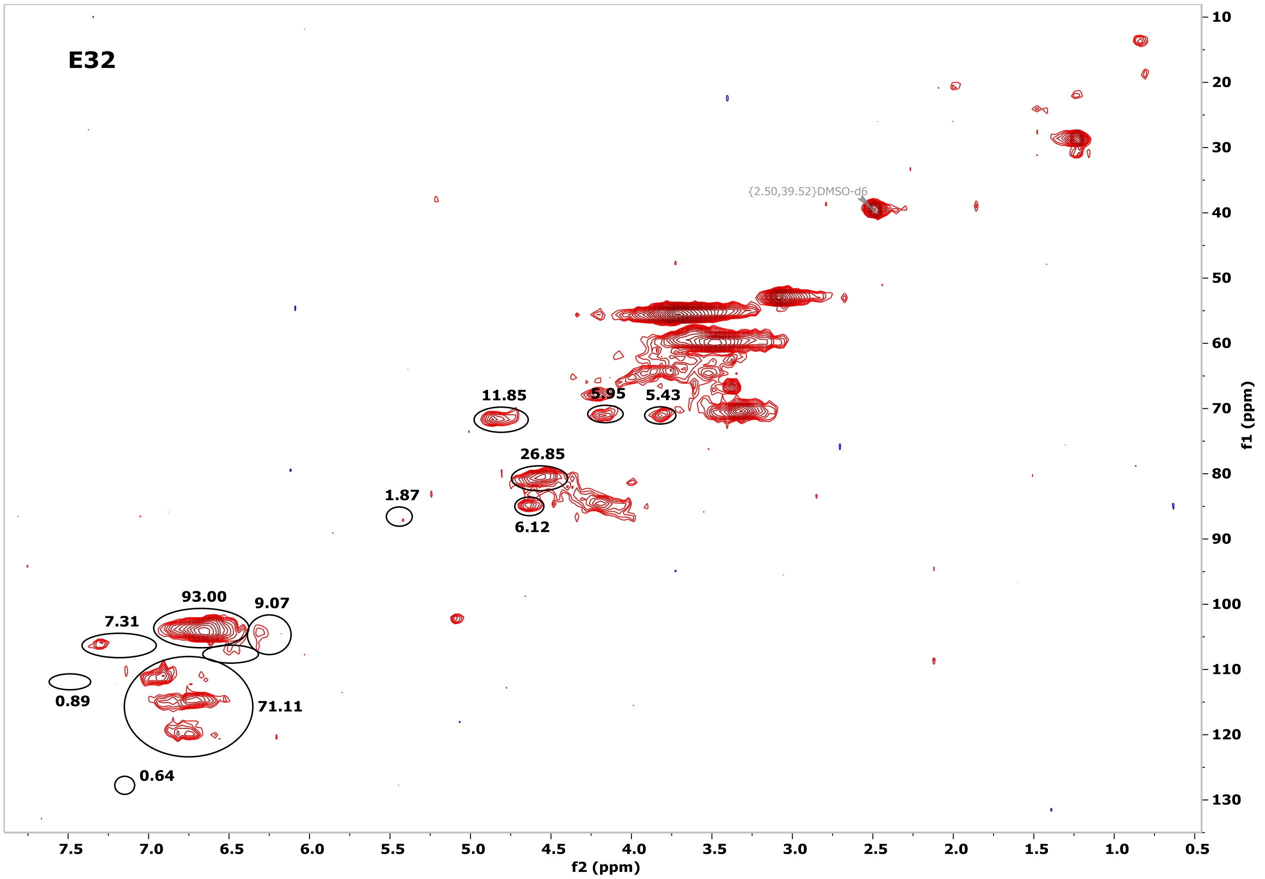

Fig. S44. 2D HSQC NMR (300 Hz, DMSO-*d*$_6$) and signal integration of lignin obtained from treating birch in the ChCh/EG/OA at 80°C for 24 h. Reaction condition: 4 g birch, 15.1 g ChCl, 13.46 g ethylene glycol, 2.86 g oxalic acid, 2.86 g water, 80°C, 4 h (See detailed reaction condition and calculating data in Table S14, E32).

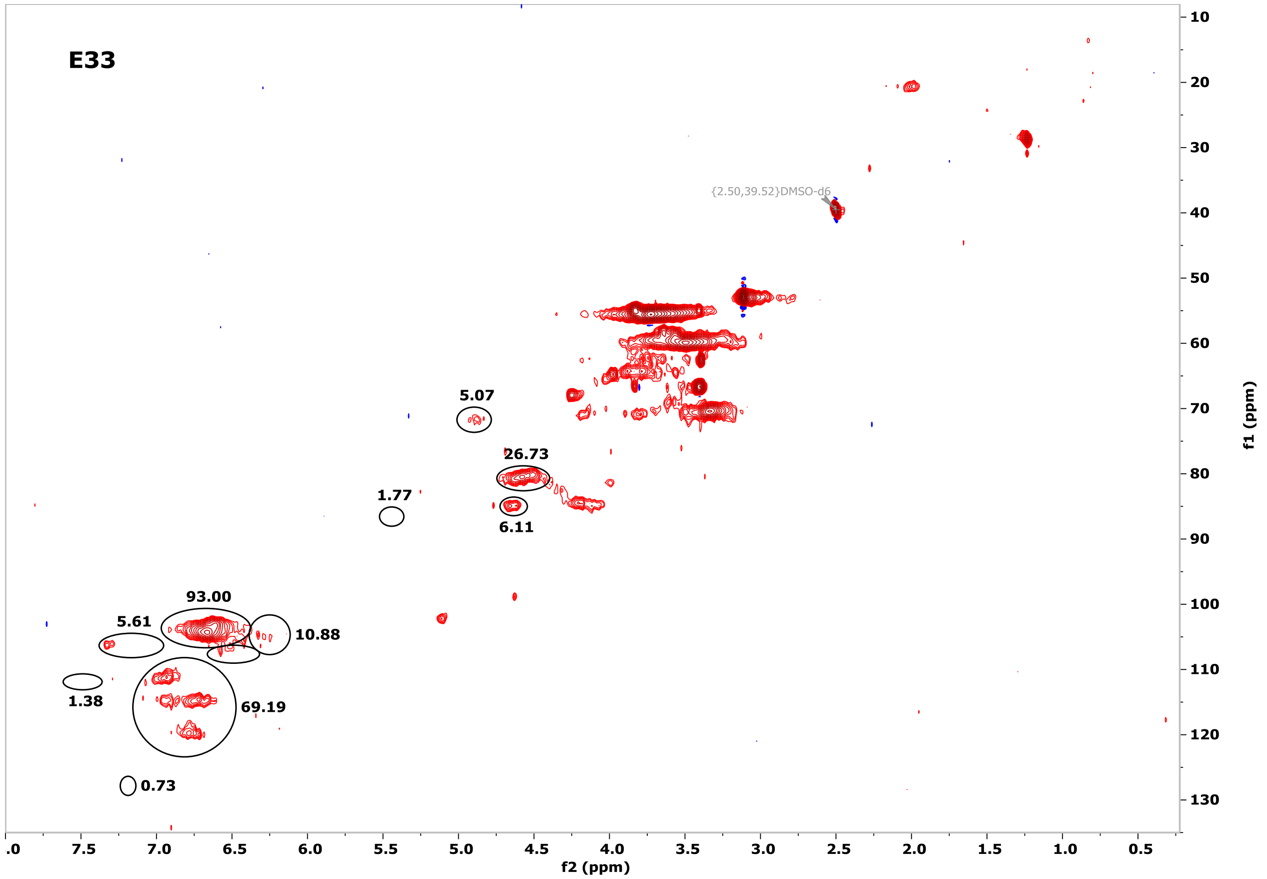

Fig. S45. 2D HSQC NMR (300 Hz, DMSO-d_6) and signal integration of lignin obtained from treating birch in the ChCh/EG/OA at 100°C for 6 h. Reaction condition: 4 g birch, 16.8 g ChCl, 14.4 g ethylene glycol, 3.12 g oxalic acid, 100°C, 6 h (See detailed reaction condition and calculating data in Table S14, E33).

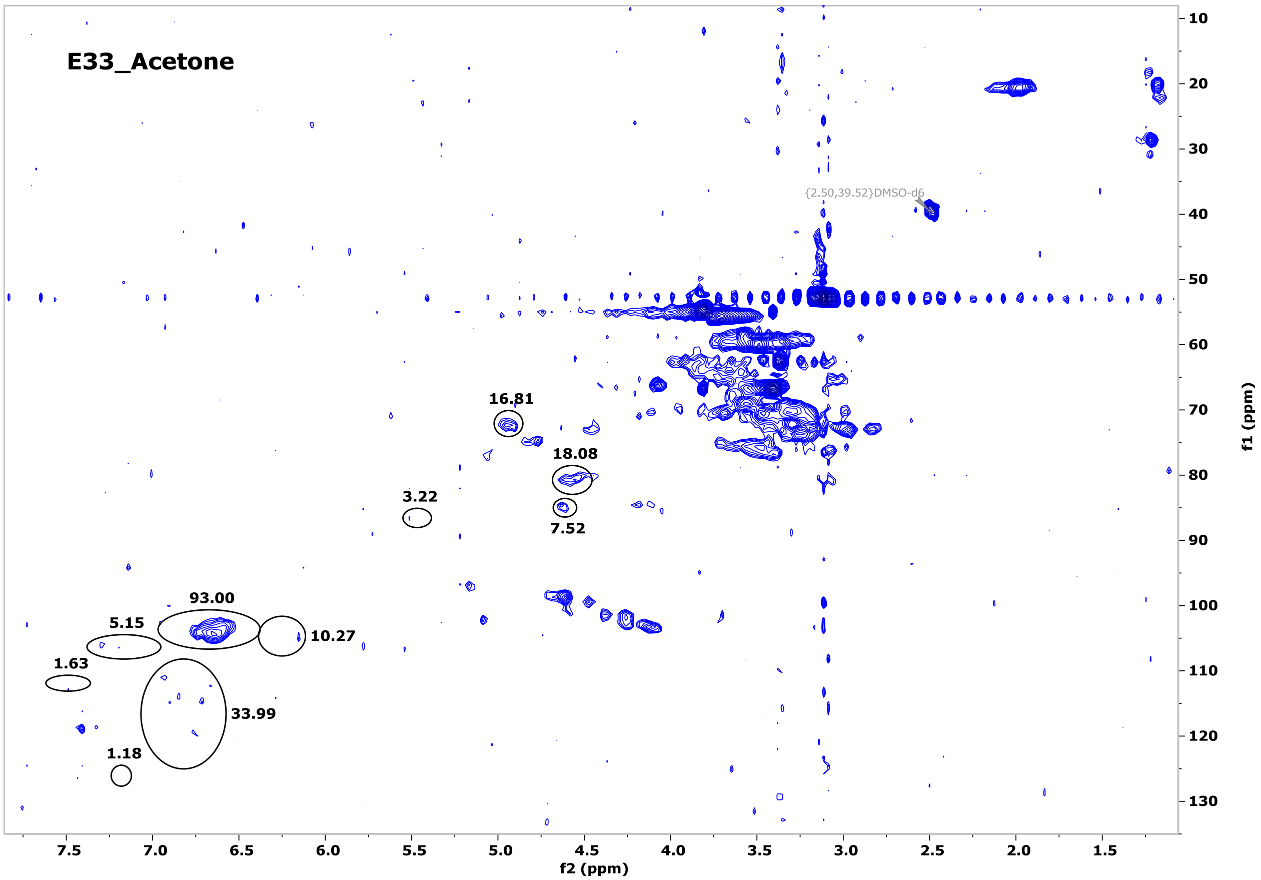

Fig. S46. 2D HSQC NMR (300 Hz, DMSO-d_6) and signal integration of extract obtained from stirring the polysaccaride residue (E30) in the aqueous 80% acetone for 2h. Reaction condition: 4 g birch, 16.8 g ChCl, 3.6 g ethylene glycol, 3.12 g oxalic acid, 100°C, 6 h (See detailed reaction condition and calculating data in Table S14, E33_A).

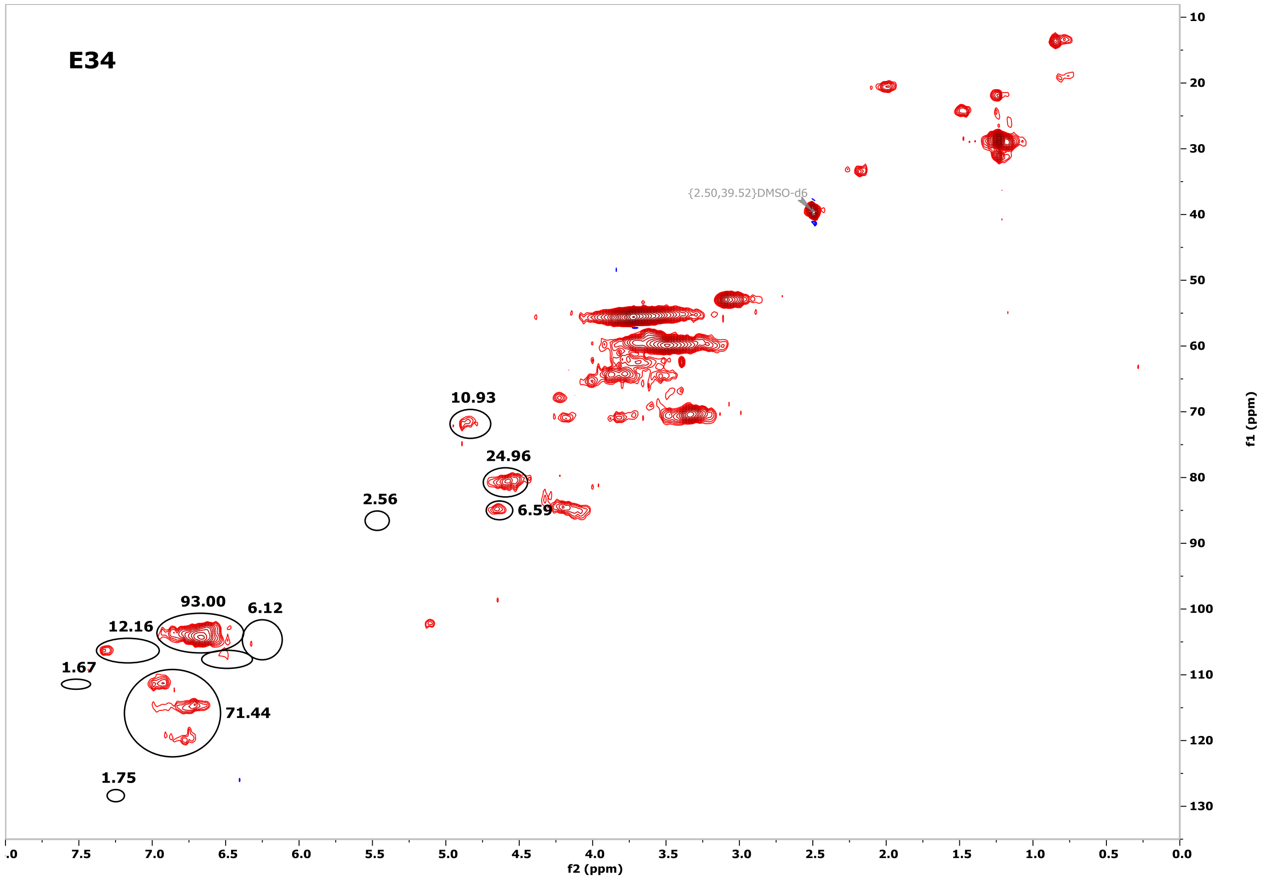

Fig. S47. 2D HSQC NMR (300 Hz, DMSO-d_6) and signal integration of lignin obtained from treating birch (> 500 μm) in the ChCh/EG/OA at 100°C for 6 h. Reaction condition: 4 g birch (> 500um), 16.8 g ChCl, 14.4 g ethylene glycol, 3.12 g oxalic acid, 100°C, 6 h (See detailed reaction condition and calculating data in Table S14, E34).

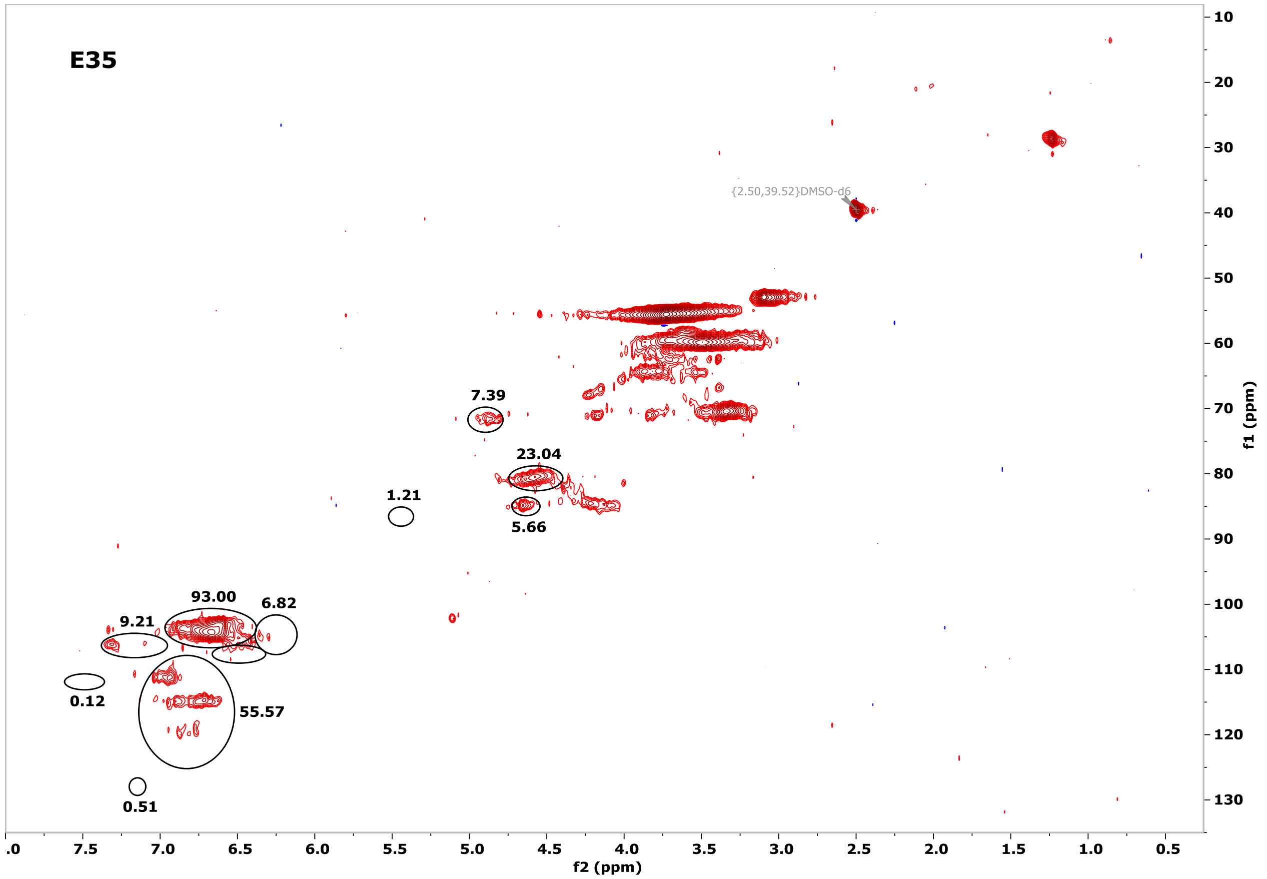

Fig. S48. 2D HSQC NMR (300 Hz, DMSO-d_6) and signal integration of lignin obtained from treating birch in the ChCh/EG/OA at 100°C for 6 h. Reaction condition: 4 g birch, 15.1 g ChCl, 13.46 g ethylene glycol, 2.86 g oxalic acid, 2.86 g water, 100°C, 6 h (See detailed reaction condition and calculating data in Table S14, E35).

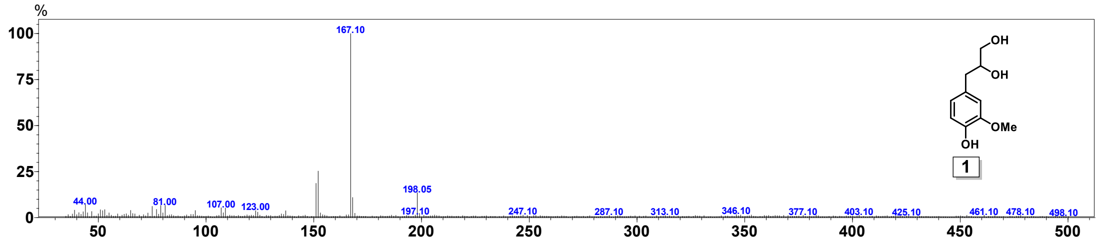
%
167.10
44.00
81.00
107.00
123.00
198.05
197.10
247.10
287.10
313.10
346.10
377.10
403.10
425.10
461.10
478.10
498.10
OH
OH
OMe
OH
1

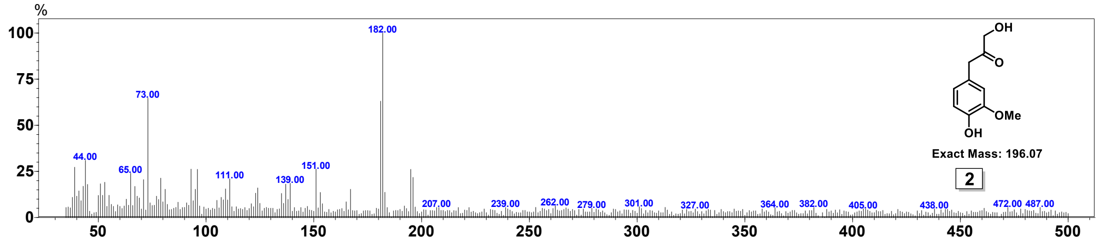
%
182.00
73.00
44.00
65.00
111.00
139.00
151.00
207.00
239.00
262.00
279.00
301.00
327.00
364.00
382.00
405.00
438.00
472.00
487.00
OH
O
OMe
OH
Exact Mass: 196.07
2

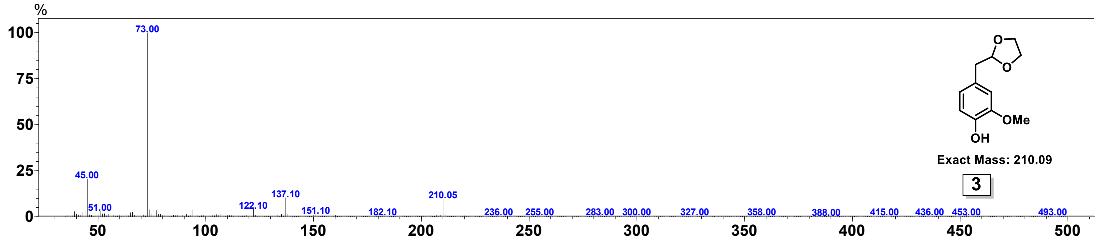
%
73.00
45.00
51.00
122.10
137.10
151.10
182.10
210.05
236.00
255.00
283.00
300.00
327.00
358.00
388.00
415.00
436.00
453.00
493.00
O
O
OMe
OH
Exact Mass: 210.09
3

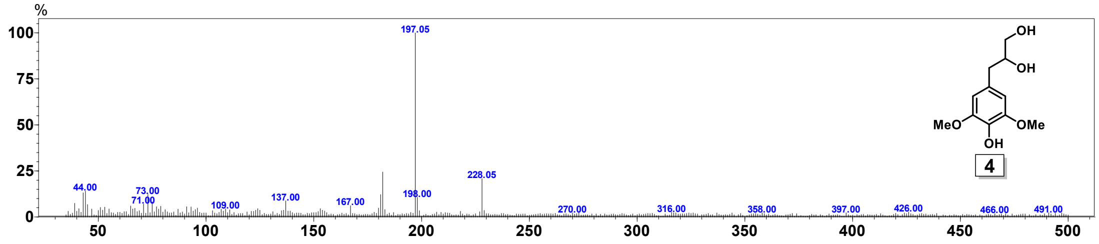
%
197.05
44.00
73.00
71.00
109.00
137.00
167.00
198.00
228.05
270.00
316.00
358.00
397.00
426.00
466.00
491.00
OH
OH
MeO
OMe
OH
4

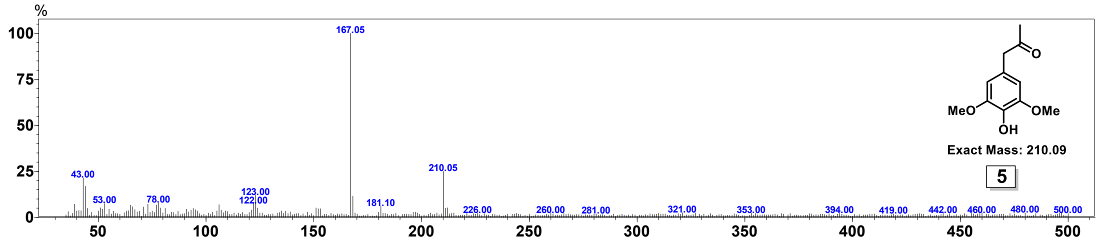
%
167.05
43.00
53.00
78.00
123.00
122.00
181.10
210.05
226.00
260.00
281.00
321.00
353.00
394.00
419.00
442.00
460.00
480.00
500.00
O
MeO
OMe
OH
Exact Mass: 210.09
5

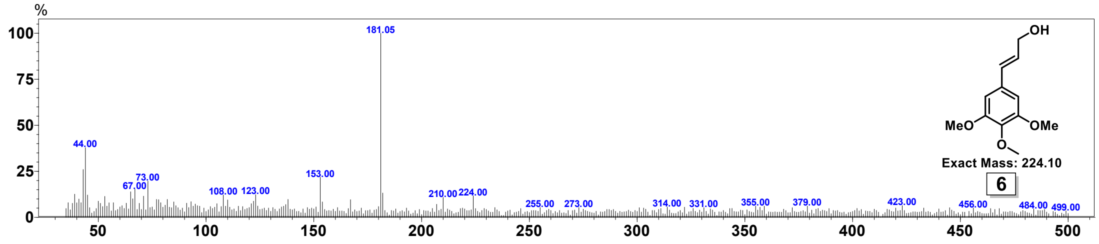
%
181.05
44.00
67.00
73.00
108.00
123.00
153.00
210.00
224.00
255.00
273.00
314.00
331.00
355.00
379.00
423.00
456.00
484.00
499.00
OH
MeO
OMe
O
Exact Mass: 224.10
6

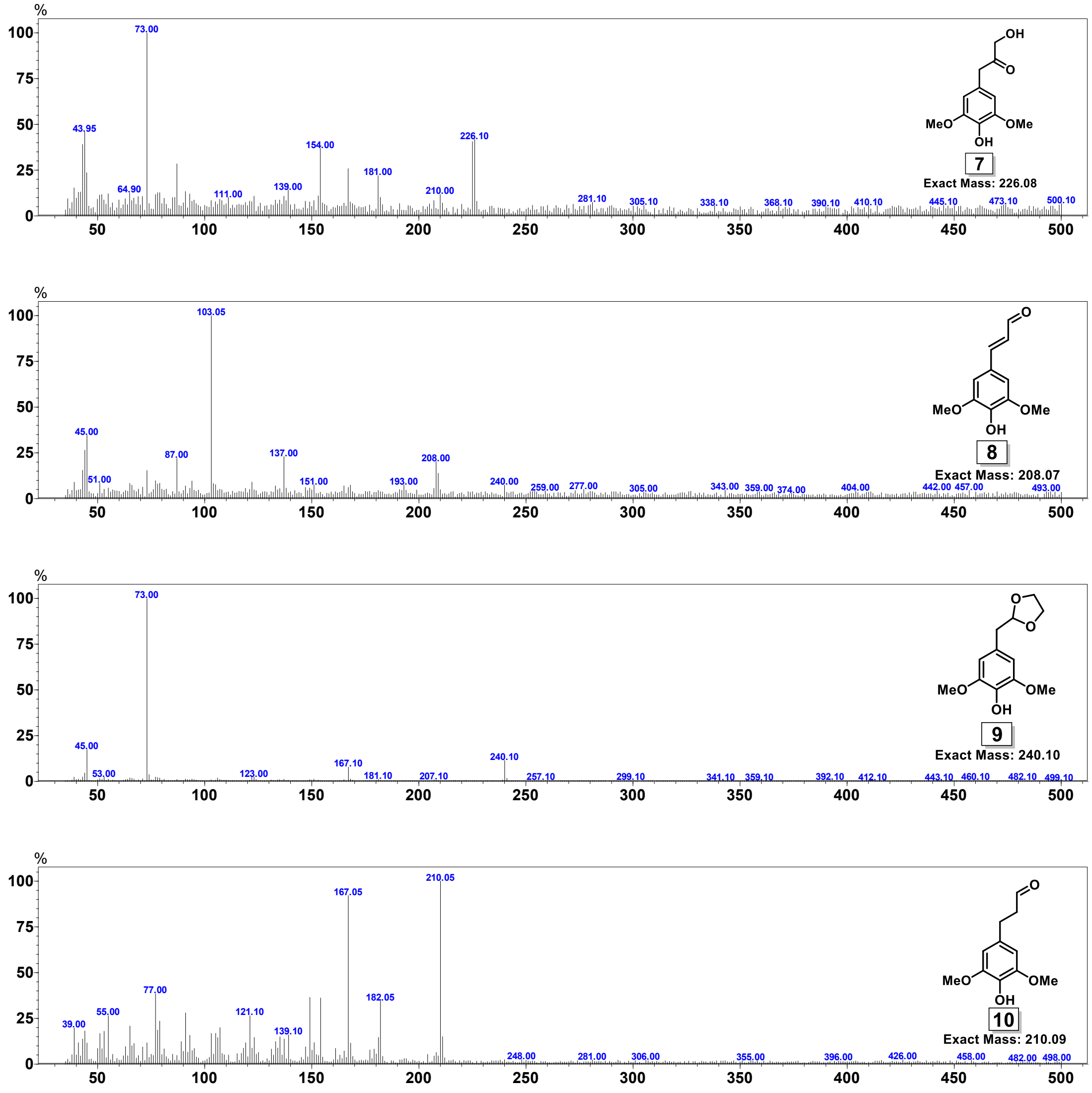

Fig. S49. The GC-MS spectra of the extracts obtained from the THF-extraction step (Step 4)

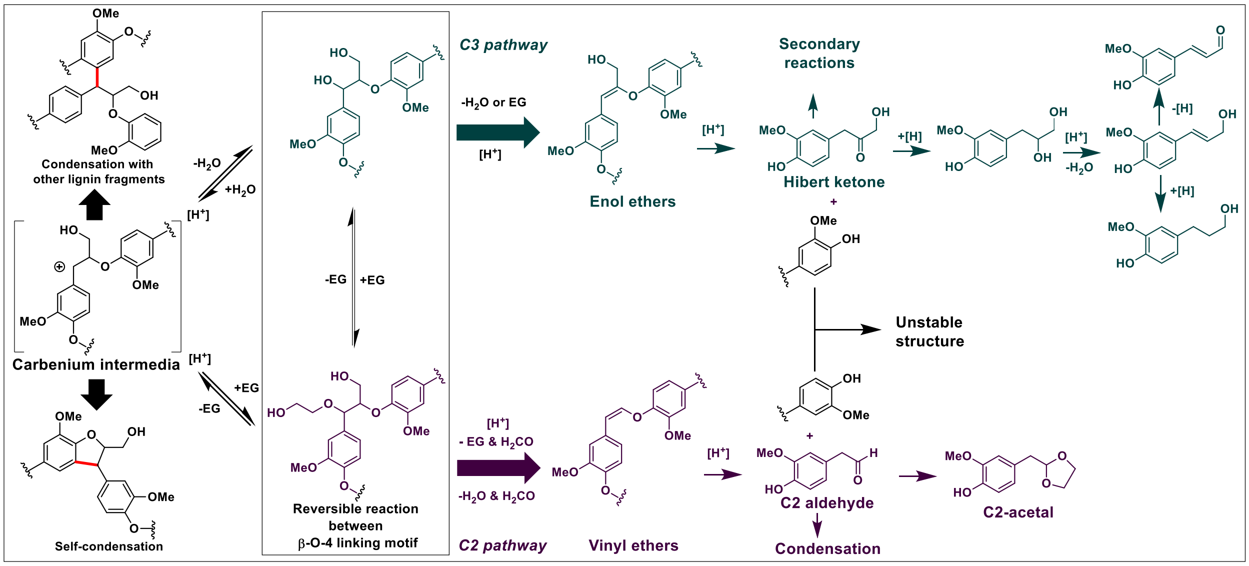

Scheme S1. Proposed mechanism for lignin degradation occurred from the biomass DES fractionation

Supplementary tables

Table S1

Compostion analysis of biomass samples used in this work

Biomass	Cellulsoe (%)	Hemicellose (%)	[a]ASL	[a]AISL
Birch	43.0	25.7	0.3	17.9
Pine	44.4	22.3	0.3	25.7
Wheat	21.4	22.4	0.3	14.1
Walnut	31.3	25.0	0.6	35.9
Bagasse	39.8	25.5	0.3	29.8

[a]ASL, acid soluble lignin, AISL, acid insoluble lignin

Table S2

The reaction conditions, lignin and residue yield and mass loss of starting material for the isolations from different water precipitation.

[a]Samples	[a]ChCl (g)	[a]EG (g)	[a]OA (g)	Birch (g)	[b]Tem (°C)	Time (h)	[b]WL (mg)	[b]YL (%)	[b]YLS (%, BS)	[b]WCR (g)	[b]RY (%)	[b]ML(%)
E1	16.8	14.4	3.12 (AH)	4	120	2	129	16.67	3.05	2.89	72.25	27.75
E2	16.8	14.4	3.12 (AH)	4	120	2	118	15.18	2.78	2.78	69.5	30.5
E3	16.8	14.4	3.12 (AH)	4	100	12	109	13.96	2.55	2.89	72.15	27.85
E4	16.8	14.4	3.12 (AH)	4	100	12	96.1	12.32	2.25	2.83	70.75	29.25

[a] **ChCl**: choline cloride, **EG**: ethylene glycol, **OA**: oxalic acid, **AH**: anhydrous oxalic acid;

[b] **Tem**: temperature, **WL**: weight of the recovered lignin, **YL**: lignin yield calculated by the weight of Klason lignin, **YLS**: lignin yield calculated by the weight of starting material, **WCR**: weight of the cellulose residue, **RY**: residue yield, **ML**: mass loss of the starting material.

Table S3

The unit percentages, linkages, EG incorporation ratio, S/G ratio for lignin obtained from different water precipitation.

Samples	[a]S	[a]S'	[a]G	[a]G'	[a]H	[b]Aa	[b]Aa'	[b]Ba	[b]Ca	[b]TA	[b]TL	[a]Sc	[a]Aa'/Aa	[a]S/G
E1	52.4	4.2	23.9	1.0	0.6	11.0	27.2	4.0	2.0	38.2	44.2	17.9	2.5	3.1
E2	54.1	4.5	23.2	1.1	0.4	8.5	29.5	3.6	1.4	38.0	43.0	16.6	3.5	3.2
E3	53.2	3.9	24.9	0.4	0.1	6.7	31.8	3.4	1.9	38.4	43.7	17.6	4.8	3.0
E4	54.7	6.6	25.2	2.3	0.6	6.8	31.0	3.4	1.7	37.8	42.9	10.7	4.6	2.9

[a] **S:** syringyl units, **S':** syringyl units with a α-ketone structures, **G:** guaiacyl structure, **G':** guaiacyl units with a α-ketone structures, **H:** *p*-hydroxylphenyl units. Percentages of lignin units were calculated from the integration of ^{13}C-^{1}H cross-signals in the aromatic region of the HSQC spectrum, relative to the total unit integration. S unit = (integration of $S_{2,6}$/2)/total units integration, S' unit = (integration of $S'_{2,6}$/2)/total units integration, G unit = integration of G_2/total units integration, G' unit = integration of G'_2/total units integration, H unit = (integration of $H_{2,6}$/2)/total units integration, **n.a:** not applicable. **Sc:** condensed syrinyl units

[b] Molar percentages of the linkage was calculated from the integration of corresponding α position divided by the integration of the total integration of aromatic units; **Aa**: aryl ether [β-O-4- α(OH)], **Aa'**: aryl ether with benzylic alkoxylation of α hydroxyl [β-O-4 α(OEG)], **Ba:** resinols (β- β), **Ca**: phenylcoumaran (β-5), **TA** (Total aryl ether linkages) = Aa+Aa', **TL** (Total linkages) = Aa + Aa' + Ca + Ba, **Aa'/Aa**: [β-O-4- α(OH)]/[β-O-4 α(OEG)], **S/G:** syringyl/guaiacyl ratio obtained by ($S_{2,6}$+$S'_{2,6}$+Sc)/(G_2+G'_2).

Table S4
The fractionation conditions, lignin yields, residues yields and mass loss of starting material after DES treatment at 80°C and different times.

Samples	[a]ChCl	[a]EG	[a]OA	Birch (g)	[b]Tem (°C)	Time (h)	[b]WL (mg)	[b]YL (%)	[b]YLS	[b]WCR (g)	[b]RY (%)	[b]ML
E5	16.8	14.4	3.12 (AH)	4	80	2	8.6	[c]1.2	0.2	3.7	91.5	8.5
E6	16.8	14.4	3.12 (AH)	4	80	6	18.6	[c]2.5	0.5	3.5	86.6	13.4
E7	16.8	14.4	3.12 (AH)	4	80	12	32.3	[c]4.4	0.8	3.2	80.1	19.9
E8[d]	16.8	14.4	3.12 (AH)	4	80	24	61.7	7.2	1.3	3.1	76.2	23.8
E9	16.8	14.4	3.12 (AH)	4	80	72	68.4	8.7	1.6	2.7	66.6	33.4

[a] **ChCl**: choline cloride, **EG**: ethylene glycol, **OA**: oxalic acid, **AH**: anhydrous oxalic acid;
[b] **Tem**: temperature, **WL**: weight of the recovered lignin, **YL**: lignin yield calculated by the weight of Klason lignin, **YLS**: lignin yield calculated by the weight of starting material, **WCR**: weight of the cellulose residue, **RY**: residue yield, **ML**: mass loss of the starting material.
[c] The yield is not corrected by subtracting the EG incorporation.
[d] Birch was milled for 6 h according to procedures of S3.1.

Table S5
The unit percentages, linkages, EG incorporation ratio, S/G ratio for lignin obtained from 80°C and 100°C at different retention times.

Samples	[a]S	[a]S'	[a]G	[a]G'	[a]H	[b]Aa	[b]Aa'	[b]Ba	[b]Ca	[b]TA	[b]TL	[a]Sc	[a]Aa'/Aa	[a]S/G
E8	58.2	4.1	27.3	0.6	0.3	10.1	37.9	4.5	1.7	48.0	54.1	9.5	3.8	2.6
E9	51.7	5.6	26.6	1.4	0.7	10.6	31.0	3.7	3.2	41.6	48.5	14.1	2.9	2.7
EMAL	71.0	2.1	19.6	0.2	0.6	64.7	0.0	3.9	1.6	64.7	70.2	6.4	0.0	4.1

[a] **S:** syringyl units, **S':** syringyl units with a α-ketone structures, **G:** guaiacyl structure, **G':** guaiacyl units with a α-ketone structures, **H:** *p*-hydroxylphenyl units. Percentages of lignin units were calculated from the integration of ^{13}C-^{1}H cross-signals in the aromatic region of the HSQC spectrum, relative to the total unit integration. S unit = (integration of $S_{2,6}$/2)/total units integration, S' unit = (integration of $S'_{2,6}$/2)/total units integration, G unit = integration of G_2/total units integration, G' unit = integration of G'_2/total units integration, H unit = (integration of $H_{2,6}$/2)/total units integration, **n.a:** not applicable. **Sc:** condensed syrinyl units

[b] Molar percentages of the linkage was calculated from the integration of corresponding α position divided by the integration of the total integration of aromatic units; **Aa**: aryl ether [β-O-4- α(OH)], **Aa'**: aryl ether with benzylic alkoxylation of α hydroxyl [β-O-4 α(OEG)], **Ba:** resinols (β- β), **Ca**: phenylcoumaran (β-5), **TA** (Total aryl ether linkages) = Aa+Aa', **TL** (Total linkages) = Aa + Aa' + Ca + Ba, **Aa'/Aa**: [β-O-4- α(OH)]/[β-O-4 α(OEG)], **S/G:** syringyl/guaiacyl ratio obtained by ($S_{2,6}$+$S'_{2,6}$+Sc)/(G_2+G'_2).

Table S6

The influence of fractionation temperature and time: reaction conditions, lignin and residue yield and mass loss of starting material.

[a]Samples	[a]ChCl (g)	[a]EG (g)	[a]OA (g)	Birch (g)	[b]Tem (°C)	Time (h)	[b]WL (mg)	[b]YL (%)	[b]YLS (%, BS)	[b]WCR (g)	[b]RY (%)	[b]ML(%)
E8	16.8	14.4	3.12 (AH)	4	80	24	57.1	7.2	1.3	3.1	78.1	21.9
E10	16.8	14.4	3.12 (AH)	4	100	2	43	5.5	1.0	3.3	82.5	17.5
E11	16.8	14.4	3.12 (AH)	4	120	2	120.5±2.5	15.5±0.35	2.9±0.054	2.8±0.025	70.15±0.65	29.9±0.60
E12	16.8	14.4	3.12 (AH)	4	140	2	229	30.0	5.5	2.7	67.5	32.5
E13	16.8	14.4	3.12 (AH)	4	160	2	343	45.2	8.3	2.3	58.3	41.7
E14	16.8	14.4	3.12 (AH)	4	180	2	495	65.4	12.0	2.2	54.5	45.5
E15	16.8	14.4	3.12 (AH)	4	200	1	493.0	65.5	12.0	2.2	55.4	44.6

[a] **ChCl**: choline cloride, **EG**: ethylene glycol, **OA**: oxalic acid, **AH**: anhydrous oxalic acid;

[b] **Tem**: temperature, **WL**: weight of the recovered lignin, **YL**: lignin yield calculated by the weight of Klason lignin, **YLS**: lignin yield calculated by the weight of starting material, **WCR**: weight of the cellulose residue, **RY**: residue yield, **ML**: mass loss of the starting material.

Table S7

The influence of fractionation temperature and time: unit percentages, linkages, EG incorporation ratio, S/G ratio, yield of lignin and mass loss of starting material.

Samples	[a]S	[a]S'	[a]G	[a]G'	[a]H	[b]Aa	[b]Aa'	[b]Ba	[b]Ca	[b]TA	[b]TL	[a]Sc	[a]Aa'/Aa	[a]S/G
E8	58.2	4.1	27.3	0.6	0.3	10.1	37.9	4.5	1.7	48.0	54.1	9.5	3.8	2.6
E10	54.6	3.8	27.6	1.2	0.5	12.8	31.4	4.5	1.9	44.2	50.5	12.2	2.5	2.6
E11	54.0±0.10	4.4±0.16	24.1±0.88	1.4±0.29	0.4±0.07	8.0±0.48	28.4±1.18	3.6±0.02	1.9±0.51	36.3±1.66	41.8±1.17	15.8±0.83	3.5±0.07	3.1±0.16
E12	48.5	5.5	20.8	0.8	0.7	4.6	20.4	2.8	2.5	25.0	30.3	23.7	4.5	3.7
E13	48.8	6.5	19.6	0.4	0.6	3.9	17.9	2.9	2.5	21.9	27.3	24.0	4.6	4.0
E14	46.3	6.5	18.9	1.1	0.7	4.4	16.6	2.0	2.2	21.1	25.3	26.4	3.8	4.2
E15	47.2	4.7	19.4	0.3	0.2	4.1	13.3	2.1	1.4	17.5	20.9	28.2	3.2	4.1

[a] **S:** syringyl units, **S':** syringyl units with a α-ketone structures, **G:** guaiacyl structure, **G':** guaiacyl units with a α-ketone structures, **H:** *p*-hydroxylphenyl units. Percentages of lignin units were calculated from the integration of ^{13}C-^{1}H cross-signals in the aromatic region of the HSQC spectrum, relative to the total unit integration. S unit = (integration of $S_{2,6}$/2)/total units integration, S' unit = (integration of $S'_{2,6}$/2)/total units integration, G unit = integration of G_2/total units integration, G' unit = integration of G'_2/total units integration, H unit = (integration of $H_{2,6}$/2)/total units integration, **n.a:** not applicable. **Sc:** condensed syrinyl units

[b] Molar percentages of the linkage was calculated from the integration of corresponding α position divided by the integration of the total integration of aromatic units; **Aa**: aryl ether [β-O-4- α(OH)], **Aa'**: aryl ether

with benzylic alkoxylation of α hydroxyl [β-O-4 α(OEG)], **Ba:** resinols (β- β), **Ca**: phenylcoumaran (β-5), **TA** (Total aryl ether linkages) = Aa+Aa', **TL** (Total linkages) = Aa + Aa' + Ca + Ba, **Aa'/Aa**: [β-O-4- α(OH)]/[β-O-4 α(OEG)], **S/G:** syringyl/guaiacyl ratio obtained by $(S_{2,6}+S'_{2,6}+Sc)/(G_2+G'_2)$.

Table S8

The influence of fractionation temperature and time: reaction conditions, lignin and residue yield and mass loss of starting material.

Samples	[a]ChCl	[a]EG	[a]OA	Birch (g)	[b]Tem (°C)	Time (h)	[b]WL (mg)	[b]YL (%)	[b]YLS (BS)	[b]WCR (g)	[b]RY (%)	[b]ML
E16	16.8	14.4	3.12 (AH)	4	100	6	80.65±2.65	10.33±0.34	1.89±0.07	3.13±0.09	78.31±2.14	21.69±2.14
E11	16.8	14.4	3.12 (AH)	4	120	2	123	15.9	2.9	2.8	70.8	29.3
E17	16.8	14.4	3.12 (AH)	4	120	4	147.5±20.5	19.01±2.69	3.5±0.47	2.73±0.035	68.15±0.85	31.9±0.9
E18	16.8	14.4	3.12 (AH)	4	120	6	173	22.4	4.1	2.8	70.5	29.5
E19	16.8	14.4	3.12 (AH)	4	120	12	181	23.5	4.3	2.6	64.3	35.8
E20	16.8	14.4	3.12 (AH)	4	140	6	280.0	36.8	6.7	3.0	75.4	24.6
E21	16.8	14.4	3.12 (AH)	4	140	12	241.0	31.6	5.8	2.5	61.4	38.6

[a] **ChCl**: choline cloride, **EG**: ethylene glycol, **OA**: oxalic acid, **AH**: anhydrous oxalic acid;

[b] **Tem**: temperature, **WL**: weight of the recovered lignin, **YL**: lignin yield calculated by the weight of Klason lignin, **YLS**: lignin yield calculated by the weight of starting material, **WCR**: weight of the cellulose residue, **RY**: residue yield, **ML**: mass loss of the starting material.

[c] The yield is not corrected by subtracting the EG incorporation.

[d] Birch was milled for 6 h according to procedures of S3.1.

Table S9

The influence of fractionation temperature and time: unit percentages, linkages, EG incorporation ratio, S/G ratio.

Samples	[a]S	[a]S'	[a]G	[a]G'	[a]H	[b]Aa	[b]Aa'	[b]Ba	[b]Ca	[b]TA	[b]TL	[a]Sc	[a]Aa'/Aa	[a]S/G
E16	54.6	3.8	27.6	1.2	0.5	12.8	31.4	4.5	1.9	44.2	50.5	12.2	2.5	2.6
E11	54.0±0.10	4.4±0.16	24.1±0.88	1.4±0.29	0.4±0.07	8.0±0.48	28.4±1.18	3.6±0.02	1.9±0.51	36.3±1.66	41.8±1.17	15.8±0.83	3.5±0.07	3.1±0.16
E17	53.8±0.85	7.1±0.40	21.7±0.48	2.1±0.97	0.3±0.27	7.3±0.68	27.7±1.05	3.4±0.23	2.2±0.07	35.1±0.37	40.7±0.52	14.9±0.31	3.8±0.50	3.5±0.04
E18	49.4±0.66	6.6±0.62	20.7±0.22	1.2±0.17	0.3±0.11	5.7±0.87	25.6±0.23	3.3±0.03	1.4±0.29	31.3±0.64	36.0±0.90	20.7±0.22	4.6±0.74	3.5±0.04
E19	51.3±0.02	6.4±0.32	19.5±0.25	1.9±0.08	0.8±0.14	3.8±0.03	25.4±0.57	2.9±0.11	1.8±0.07	29.2±0.59	33.9±0.55	20.0±0.27	6.7±0.10	4.0±0.05
E20	50.6	5.9	19.1	0.8	1.4	4.6	19.6	2.8	2.1	24.2	29.2	22.1	4.2	4.1
E21	50.9	5.9	18.0	2.5	0.9	4.0	19.9	2.9	3.0	23.8	29.7	21.9	5.0	4.4

[a] **S:** syringyl units, **S':** syringyl units with a α-ketone structures, **G:** guaiacyl structure, **G':** guaiacyl units with a α-ketone structures, **H:** *p*-hydroxylphenyl units. Percentages of lignin units were calculated from the integration of ^{13}C-^{1}H cross-signals in the aromatic region of the HSQC spectrum, relative to the total unit integration. S unit = (integration of $S_{2,6}$/2)/total units integration, S' unit = (integration of $S'_{2,6}$/2)/total units integration, G unit = integration of G_2/total units integration, G' unit = integration of G'_2/total units integration, H unit = (integration of $H_{2,6}$/2)/total units integration, **n.a:** not applicable. **Sc:** condensed syrinyl units

[b] Molar percentages of the linkage was calculated from the integration of corresponding α position divided by the integration of the total integration of aromatic units; **Aa**: aryl ether [β-O-4- α(OH)], **Aa'**: aryl ether with benzylic alkoxylation of α hydroxyl [β-O-4 α(OEG)], **Ba:** resinols (β- β), **Ca**: phenylcoumaran (β-5), **TA** (Total aryl ether linkages) = Aa+Aa', **TL** (Total linkages) = Aa + Aa' + Ca + Ba, **Aa'/Aa**: [β-O-4- α(OH)]/[β-O-4 α(OEG)], **S/G:** syringyl/guaiacyl ratio obtained by ($S_{2,6}$+$S'_{2,6}$+Sc)/(G_2+G'_2).

Table S10

The influence of tunable DES constituents: reaction conditions, lignin and residue yield and mass loss of starting material.

[a]Samples	[a]ChCl (g)	[a]EG (g)	[a]OA (g)	Birch (g)	[b]Tem (°C)	Time (h)	[b]WL (mg)	[b]YL (%)	[b]YLS (%, BS)	[b]WCR (g)	[b]RY (%)	[b]ML(%)
E22	20.85	0	13.48	4	120	4	507	69.3	12.7	2.3	58.4	41.6
E17	16.8	14.4	3.12 (AH)	4	120	4	147.5±20.5	19.01±2.69	3.5±0.47	2.73±0.035	68.15±0.85	31.9±0.9
E23	11.23	19.86	3.12	4	120	4	142	18.2	3.4	2.8	70.2	29.8
E24	15.1	13.4	5.7	4	120	4	226.7	29.6	5.4	2.4	60.9	39.1
E25	0	31.2	3.12	4	120	4	25.5	3.3	0.6	3.9	98.1	1.9
E26	0	22.88	11.44	4	100	12	158.7	20.1	3.7	2.9	71.5	28.5

[a] **ChCl**: choline cloride, **EG**: ethylene glycol, **OA**: oxalic acid, **AH**: anhydrous oxalic acid;

[b] **Tem**: temperature, **WL**: weight of the recovered lignin, **YL**: lignin yield calculated by the weight of Klason lignin, **YLS**: lignin yield calculated by the weight of starting material, **WCR**: weight of the cellulose residue, **RY**: residue yield, **ML**: mass loss of the starting material.

Table S11

The influence of tunable DES constituents: unit percentages, linkages, EG incorporation ratio, S/G ratio, yield of lignin and mass loss of starting material.

Samples	[a]S	[a]S'	[a]G	[a]G'	[a]H	[b]Aa	[b]Aa'	[b]Ba	[b]Ca	[b]TA	[b]TL	[a]Sc	[a]Aa'/Aa	[a]S/G
E22	n.a	n.a	n.a	n.a	n.a	n.a	n.a	n.a	n.a	n.a	n.a	n.a	n.a	n.a
E17	53.8±0.85	7.1±0.40	21.7±0.48	2.1±0.97	0.3±0.27	7.3±0.68	27.7±1.05	3.4±0.23	2.2±0.07	35.1±0.37	40.7±0.52	14.9±0.31	3.8±0.50	3.5±0.04
E23	52.5	3.3	23.9	0.3	0.1	6.2	31.4	3.6	1.3	37.6	42.6	17.7	5.1	3.2
E24	49.7	6.1	20.9	1.3	0.3	5.7	22.9	3.1	2.2	28.6	33.9	20.3	4.0	3.7
E25	52.6	3.3	28.1	1.1	1.1	12.0	PC30.3	3.7	2.2	42.3	48.2	12.2	2.5	2.5
E26	56.3	5.8	24.0	1.6	0.5	4.2	36.5	3.6	1.8	40.7	46.1	9.7	8.6	3.1

[a] **S:** syringyl units, **S':** syringyl units with a α-ketone structures, **G:** guaiacyl structure, **G':** guaiacyl units with a α-ketone structures, **H:** *p*-hydroxylphenyl units. Percentages of lignin units were calculated from the integration of ^{13}C-^{1}H cross-signals in the aromatic region of the HSQC spectrum, relative to the total unit integration. S unit = (integration of $S_{2,6}$/2)/total units integration, S' unit = (integration of $S'_{2,6}$/2)/total units integration, G unit = integration of G_2/total units integration, G' unit = integration of G'_2/total units integration, H unit = (integration of $H_{2,6}$/2)/total units integration, **n.a:** not applicable. **Sc:** condensed syrinyl units

[b] Molar percentages of the linkage was calculated from the integration of corresponding α position divided by the integration of the total integration of aromatic units; **Aa**: aryl ether [β-O-4- α(OH)], **Aa'**: aryl ether with benzylic alkoxylation of α hydroxyl [β-O-4 α(OEG)], **Ba:** resinols (β- β), **Ca**: phenylcoumaran (β-5), **TA** (Total aryl ether linkages) = Aa+Aa', **TL** (Total linkages) = Aa + Aa' + Ca + Ba, **Aa'/Aa**: [β-O-4- α(OH)]/[β-O-4 α(OEG)], **S/G:** syringyl/guaiacyl ratio obtained by ($S_{2,6}$+$S'_{2,6}$+Sc)/(G_2+G'_2).

Table S12

DES extraction to various lignocellulose species: reaction conditions, lignin and residue yield and mass loss of starting material.

[a]Samples	[a]ChCl (g)	[a]EG (g)	[a]OA (g)	Birch (g)	[b]Tem (°C)	Time (h)	[b]WL (mg)	[b]YL (%)	[b]YLS (%, BS)	[b]WCR (g)	[b]RY (%)	[b]ML (%)
E12	16.8	14.4	3.12 (AH)	4 (birch)	140	2	229.3	30.2	5.5	2.7	67.5	32.5
E27	16.8	14.4	3.12 (AH)	4 g (pine)	140	2	233.3	21.0	5.4	2.8	69.6	30.4
E28	16.8	14.4	3.12 (AH)	4 g (wheat)	140	2	270.8	44.8	6.5	2.3	58.7	41.3
E29	16.8	14.4	3.12 (AH)	4 g (walnut)	140	2	612	40.0	14.6	2.5	62.8	37.2
E30	16.8	14.4	3.12 (AH)	4 g(bagasse)	140	2	276.6	21.6	6.5	2.9	72.4	27.6

[a] **ChCl**: choline cloride, **EG**: ethylene glycol, **OA**: oxalic acid, **AH**: anhydrous oxalic acid;

[b] **Tem**: temperature, **WL**: weight of the recovered lignin, **YL**: lignin yield calculated by the weight of Klason lignin, **YLS**: lignin yield calculated by the weight of starting material, **WCR**: weight of the cellulose residue, **RY**: residue yield, **ML**: mass loss of the starting material.

Table S13

DES extraction to various lignocellulose species: unit percentages, linkages, EG incorporation ratio, S/G ratio, yield of lignin and mass loss of starting material.

Samples	[a]S	[a]S'	[a]G	[a]G'	[a]H	[b]Aa	[b]Aa'	[b]Ba	[b]Ca	[b]TA	[b]TL	[a]Sc	[a]Aa'/Aa	[a]S/G
E12	48.5	5.5	20.8	0.8	0.7	4.6	20.4	2.8	2.5	25.0	30.3	23.7	4.5	3.7
E27	0.0	0.0	95.8	3.7	0.6	3.7	27.7	0.7	14.1	31.5	46.3	0.0	7.4	0.0
E28	30.6	12.2	35.5	5.7	2.0	4.4	23.4	0.9	3.4	27.8	32.1	14.0	5.3	1.6
E29	43.8	4.3	28.8	4.4	2.1	7.3	22.4	0.3	4.5	29.7	34.5	16.6	3.1	2.2
E30	40.1	5.8	33.9	3.7	4.7	7.1	29.8	3.0	7.8	36.9	47.7	11.9	4.2	1.7

[a] **S:** syringyl units, **S':** syringyl units with a α-ketone structures, **G:** guaiacyl structure, **G':** guaiacyl units with a α-ketone structures, **H:** *p*-hydroxylphenyl units. Percentages of lignin units were calculated from the integration of ^{13}C-^{1}H cross-signals in the aromatic region of the HSQC spectrum, relative to the total unit integration. S unit = (integration of $S_{2,6}/2$)/total units integration, S' unit = (integration of $S'_{2,6}/2$)/total units integration, G unit = integration of G_2/total units integration, G' unit = integration of G'_2/total units integration, H unit = (integration of $H_{2,6}/2$)/total units integration, **n.a:** not applicable. **Sc:** condensed syrinyl units

[b] Molar percentages of the linkage was calculated from the integration of corresponding α position divided by the integration of the total integration of aromatic units; **Aa**: aryl ether [β-O-4- α(OH)], **Aa'**: aryl ether with benzylic alkoxylation of α hydroxyl [β-O-4 α(OEG)], **Ba:** resinols (β- β), **Ca**: phenylcoumaran (β-5), **TA** (Total aryl ether linkages) = Aa+Aa', **TL** (Total linkages) = Aa + Aa' + Ca + Ba, **Aa'/Aa**: [β-O-4- α(OH)]/[β-O-4 α(OEG)], **S/G:** syringyl/guaiacyl ratio obtained by $(S_{2,6}+S'_{2,6}+Sc)/(G_2+G'_2)$.

Table S14

The influence of water addition and particle size**:** reaction conditions, lignin and residue yield and mass loss of starting material.

Sample	[a]ChCl	[a]EG	[a]OA	Water (g)	Birch (g)	[b]Tem (°C)	[b]Time (h)	[b]WL (mg)	[b]YL (%)	[b]YLS (%, BS)	[b]WCR (g)	[b]RY (%)	[b]ML (%)
E31	16.8	14.4	3.12 (AH)	0	4 g (ball milled)	80	24	127	16.0	3.0	3.1	76.8	23.2
E32	15.1	13.46	2.86	2.86	4	80	24	61.7	7.9	1.4	3.0	76.2	23.8
E33	16.8	14.4	3.12 (AH)	0	4	100	6	158	20.3	3.8	2.9	72.4	27.6
E33_A	16.8	14.4	3.12 (AH)	0	4	100	6	60	7.8	1.4	n.a	n.a	n.a
E34	16.8	14.4	3.12 (AH)	0	4 (> 500um)	100	6	83.3	10.8	2.0	2.3	76.2	23.8
E35	15.1	13.46	2.86 (AH)	2.86	4	100	6	142	18.3	3.4	2.6	65.8	34.3

[a] **ChCl**: choline cloride, **EG**: ethylene glycol, **OA**: oxalic acid, **AH**: anhydrous oxalic acid;
[b] **Tem**: temperature, **WL**: weight of the recovered lignin, **YL**: lignin yield calculated by the weight of Klason lignin, **YLS**: lignin yield calculated by the weight of starting material, **WCR**: weight of the cellulose residue, **RY**: residue yield, **ML**: mass loss of the starting material.

Table S15

The influence of water addition and particle size: unit percentages, linkages, EG incorporation ratio, S/G ratio.

Samples	[a]S	[a]S'	[a]G	[a]G'	[a]H	[b]Aa	[b]Aa'	[b]Ba	[b]Ca	[b]TA	[b]TL	[a]Sc	[a]Aa'/Aa	[a]S/G
E31	56.5	3.9	28.5	0.6	1.6	11.2	38.1	4.3	1.6	49.3	55.2	9.0	3.4	2.4
E32	53.2	4.2	26.9	1.0	0.4	13.6	30.7	3.5	2.1	44.3	50.0	14.3	2.3	2.7
E33	51.0	3.1	25.0	1.5	0.4	5.6	29.3	3.4	1.9	34.9	40.2	18.9	5.3	2.9
E33_A	64.2	3.6	15.2	2.3	0.7	23.2	25.0	5.2	4.4	48.2	57.8	14.1	1.1	5.4
E34	51.1	6.7	25.5	1.8	1.0	12.0	27.4	3.6	2.8	39.4	45.9	13.9	2.3	2.8
E35	56.3	5.6	22.2	0.1	0.3	8.9	27.9	3.4	2.2	36.8	42.5	15.4	3.1	3.5

[a] **S:** syringyl units, **S':** syringyl units with a α-ketone structures, **G:** guaiacyl structure, **G':** guaiacyl units with a α-ketone structures, **H:** *p*-hydroxylphenyl units. Percentages of lignin units were calculated from the integration of ^{13}C-^{1}H cross-signals in the aromatic region of the HSQC spectrum, relative to the total unit integration. S unit = (integration of $S_{2,6}$/2)/total units integration, S' unit = (integration of $S'_{2,6}$/2)/total units integration, G unit = integration of G_2/total units integration, G' unit = integration of G'_2/total units integration, H unit = (integration of $H_{2,6}$/2)/total units integration, **n.a:** not applicable. **Sc:** condensed syrinyl units

[b] Molar percentages of the linkage was calculated from the integration of corresponding α position divided by the integration of the total integration of aromatic units; **Aa**: aryl ether [β-O-4- α(OH)], **Aa'**: aryl ether with benzylic alkoxylation of α hydroxyl [β-O-4 α(OEG)], **Ba:** resinols (β- β), **Ca**: phenylcoumaran (β-5), **TA** (Total aryl ether linkages) = Aa+Aa', **TL** (Total linkages), **Aa'/Aa**: [β-O-4- α(OH)]/[β-O-4 α(OEG)], **S/G:** syringyl/guaiacyl ratio obtained by ($S_{2,6}$+$S'_{2,6}$+Sc)/(G_2+G'_2).

Table S16

Organic extracts from THF extraction of aqueous fractions: reaction temperature, time, unit percentages, linkages, EG incorporation ratio, S/G ratio of the oil obtained from THF extraction of the liquid fractionation

Lignin oils	[a]Tem (°C)	Time (h)	[a]S	[a]S'	[a]Sc	[a]G	[a]G'	[a]H	[b]Aa	[b]Aa'	[b]TA	[b]Aa'/Aa	[b]S/G
E11_O	120	2	74.8	9.3	10.1	2.2	1.4	2.1	3.4	18.1	21.4	5.3	43.7
E17_O	120	4	69.1	7.0	14.4	6.3	2.0	1.3	5.3	15.4	20.7	2.9	14.4
E18_O	120	6	46.6	9.1	18.7	20.6	2.8	2.3	4.6	15.0	19.6	3.3	3.6
E19_O	120	12	39.9	10.1	26.5	20.0	2.0	1.5	4.1	12.8	17.0	3.1	3.8
E13_O	160	2	42.4	8.9	20.2	26.3	1.5	0.7	5.9	6.4	12.3	1.1	2.7
E15_O	200	1	30.9	7.6	21.1	38.8	1.3	0.3	2.6	6.7	9.3	2.5	1.5

[a] **S:** syringyl units, **S':** syringyl units with a α-ketone structures, **G:** guaiacyl structure, **G':** guaiacyl units with a α-ketone structures, **H:** *p*-hydroxylphenyl units. Percentages of lignin units were calculated from the integration of ^{13}C-^{1}H cross-signals in the aromatic region of the HSQC spectrum, relative to the total unit integration. S unit = (integration of $S_{2,6}/2$)/total units integration, S' unit = (integration of $S'_{2,6}/2$)/total units integration, G unit = integration of G_2/total units integration, G' unit = integration of G'_2/total units integration, H unit = (integration of $H_{2,6}/2$)/total units integration, **n.a:** not applicable. **Sc:** condensed syrinyl units

[b] Molar percentages of the linkage was calculated from the integration of corresponding α position divided by the integration of the total integration of aromatic units; **Aa**: aryl ether [β-O-4- α(OH)], **Aa'**: aryl ether with benzylic alkoxylation of α hydroxyl [β-O-4 α(OEG)], **TA** (Total aryl ether linkages) = Aa+Aa', **Aa'/Aa**: [β-O-4- α(OH)]/[β-O-4 α(OEG)], **S/G:** syringyl/guaiacyl ratio obtained by ($S_{2,6}$+$S'_{2,6}$+Sc)/(G_2+G'_2).

References

[1] H. Sadeghifar, T. Wells, R.K. Le, F. Sadeghifar, J.S. Yuan, A. Jonas Ragauskas, Fractionation of Organosolv Lignin Using Acetone:Water and Properties of the Obtained Fractions, ACS Sustain. Chem. Eng. 5 (2017) 580–587. https://doi.org/10.1021/acssuschemeng.6b01955.

[2] J. Sameni, S. Krigstin, M. Sain, Solubility of Lignin and Acetylated Lignin in Organic Solvents, BioResources. 12 (2017) 1548–1565. https://doi.org/10.15376/biores.12.1.1548-1565.

[3] Q. Xia, Y. Liu, J. Meng, W. Cheng, W. Chen, S. Liu, Y. Liu, J. Li, H. Yu, Multiple hydrogen bond coordination in three-constituent deep eutectic solvents enhances lignin fractionation from biomass, Green Chem. 20 (2018) 2711–2721.

[4] O.S. Hammond, D.T. Bowron, K.J. Edler, The effect of water upon deep eutectic solvent nanostructure: an unusual transition from ionic mixture to aqueous solution, Angew. Chemie. 129 (2017) 9914–9917.

[5] J.R. Brusas, E.M.B. Dela Pena, Hygroscopicity of 1: 2 Choline chloride: Ethylene glycol deep eutectic solvent: A hindrance to its electroplating industry adoption, J. Electrochem. Sci. Technol. 12 (2021) 387–397.

[6] Z. Wang, X. Zhu, P.J. Deuss, The effect of ball milling on birch, pine, reed, walnut shell enzymatic hydrolysis recalcitrance and the structure of the isolated residual enzyme lignin, Ind. Crops Prod. 167 (2021) 113493.

[7] Hong, S.; Shen, X.; Pang, B.; Xue, Z.; Cao, X:; Wen,J.; Sun, Z.; Lam, S.; Yuan, T.; Sun, R. In-depth interpretation of the structural changes of lignin and formation of diketones during acidic deep eutectic solvent pretreatment. Green Chem. 22, (2020), 1851–1858.

[8] Yokoyama, T.; Matsumoto, Y. Revisiting the mechanism of β-O-4 bond cleavage during acidolysis of lignin. Part 1: Kinetics of the formation of enol ether from non-phenolic C6-C2 type model compounds. Holzforschung 62, (2008), 164–168.

[9] Kulka, M.; Hibbert, H. Studies on Lignin and Related Compounds. LXVII. Isolation and Identification of 1-(4-Hydroxy-3,5-dimethoxyphenyl)-2-propanone and 1-(4-Hydroxy-3-methoxyphenyl)-2-propanone from Maple Wood Ethanolysis Products. Metabolic Changes in Lower and Higher Plants. J. Am. Chem. Soc. 65, (1943), 1180–1185. https://doi.org/10.1021/ja01246a046.

[10] Lundquist, K.; Lundgren, R.; Danielsen, J.; Haaland, A.; Svensson, S. Acid degradation of lignin. Acta Chem Scand 26, (1972), 2005–2023.

[11] Li, S.; Lundquist, K.; Westermark, U. Cleavage of arylglycerol ß-aryl ethers under neutral and acid conditions. Nord. Pulp Pap. Res. J. 15, (2000), 292–299.

[12] Ito, H.; Imai, T.; Lundquist, K.; Yokoyama, T.; Matsumoto, Y. Revisiting the mechanism of β-O-4 bond cleavage during acidolysis of lignin. Part 3: Search for the rate-determining step of a non-phenolic C6-C3 type model compound. J. Wood Chem. Technol. 31, (2011), 172–182.

[13] Deuss, P.J.; Kugge, C. "Lignin-first" catalytic valorization for generating higher value from lignin. Chem Catal. (1), 2021, 8–11.

[14] De Santi, A.; Galkin, M. V.; Lahive, C.W.; Deuss, P.J.; Barta, K. Lignin-first fractionation of

softwood lignocellulose using a mild dimethyl carbonate and ethylene glycol organosolv process. ChemSusChem (13), 2020, 4468–4477.
